# Supplementary material for: Synthesis, Antifungal Activity, 3D-QSAR and Controlled Release on Hydrotalcite Study of Longifolene-Derived Diphenyl Ether Carboxylic Acid Compounds
Source: Molecules. 2023 Feb 16;28(4):1911. doi: 10.3390/molecules28041911 (PMC9968029; doi:10.3390/molecules28041911)
Supplement: Supplementary file 1 [file molecules-28-01911-s001.zip › molecules-2217728-supplementary.pdf]

Supporting Information

Synthesis, Antifungal Activity, 3D-QSAR and Controlled Release on Hydrotalcite Study of Longifolene-Derived Diphenyl Ether Carboxylic Acid Compounds

Xiaocui Wu[1], Guishan Lin[1,*], Wengui Duan[1,*], Baoyu Li[1], Yucheng Cui[1], Bo Cen[1], Fuhou Lei[2]

[1]School of Chemistry and Chemical Engineering, Guangxi University, Nanning 530004, Guangxi, China

[2]Guangxi Key Laboratory of Chemistry and Engineering of Forest Products, Guangxi Collaborative Innovation Center for Chemistry and Engineering of Forest Products, Guangxi University for Nationalities, Nanning 530004, Guangxi, China

* Corresponding Authors: gslin@gxu.edu.cn (Guishan Lin),

wgduan@gxu.edu.cn (Wengui Duan).

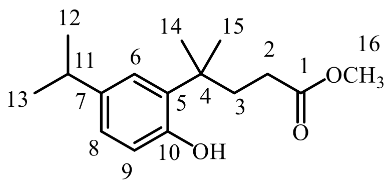

Intermediate **5**, white solid; yield 97.5%; m. p. 104.3 – 105.2 °C; FT-IR (KBr, cm^{-1}): 3379 (O–H), 3055, 3026 (Ar–H), 2956, 2918, 2865 (C-H), 1716 (C=O), 1607, 1508, 1453, 1423, 1349, 1296, 1236, 1208, 1165 (Ar-C=C); ^{1}H NMR (500 MHz, $CDCl_3$) δ 7.01 (d, J = 2.1 Hz, 1H, C_6-H), 6.92 (dd, J = 8.1, 2.1 Hz, 1H, C_8-H), 6.60 (d, J = 8.1 Hz, 1H, C_9-H), 3.59 (s, 3H, C_{16}-H), 2.82 (dt, J = 13.8, 6.9 Hz, 1H, C_{11}-H), 2.26 – 2.21 (m, 2H, C_2-H), 2.10 – 2.05 (m, 2H, C_3-H), 1.39 (s, 6H, C_{14}-H, C_{15}-H), 1.21 (d, J = 6.9 Hz, 6H, C_{12}-H, C_{13}-H); ^{13}C NMR (126 MHz, $CDCl_3$) δ 175.33, 152.20, 140.59, 132.76, 126.37, 124.57, 116.39, 51.54, 37.49, 35.41, 33.52, 30.60, 27.90, 24.28; ESI-MS m/z: 263.1650 [M + H^{+}].

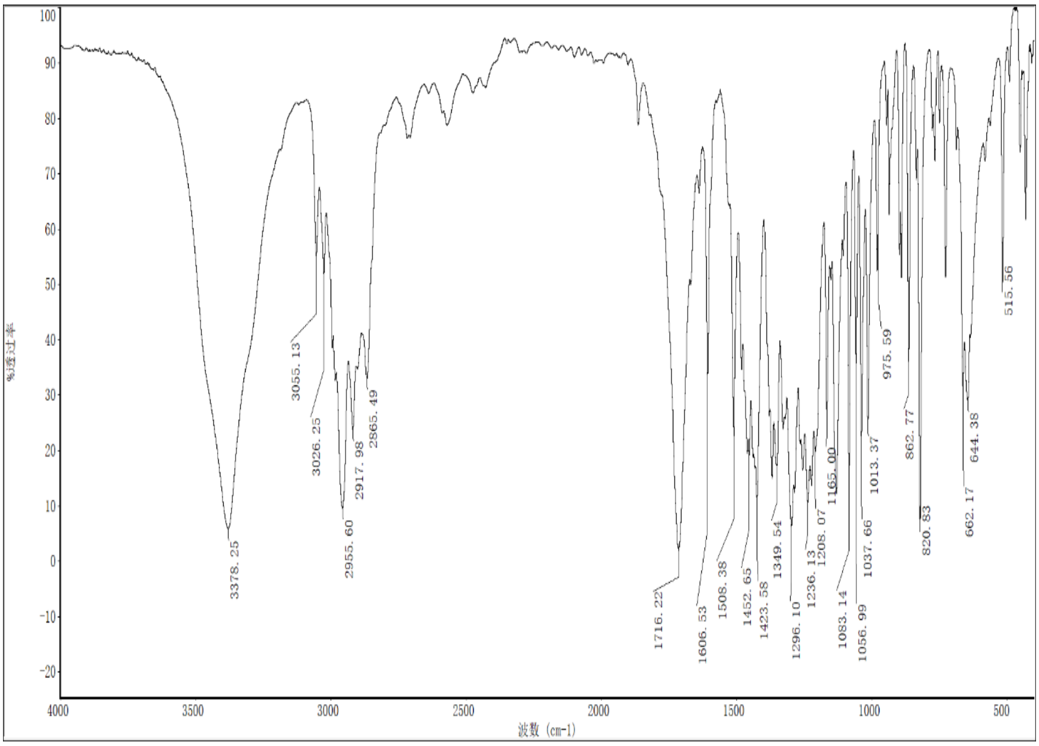

Figure S1. FT-IR spectrum of intermediate **5**.

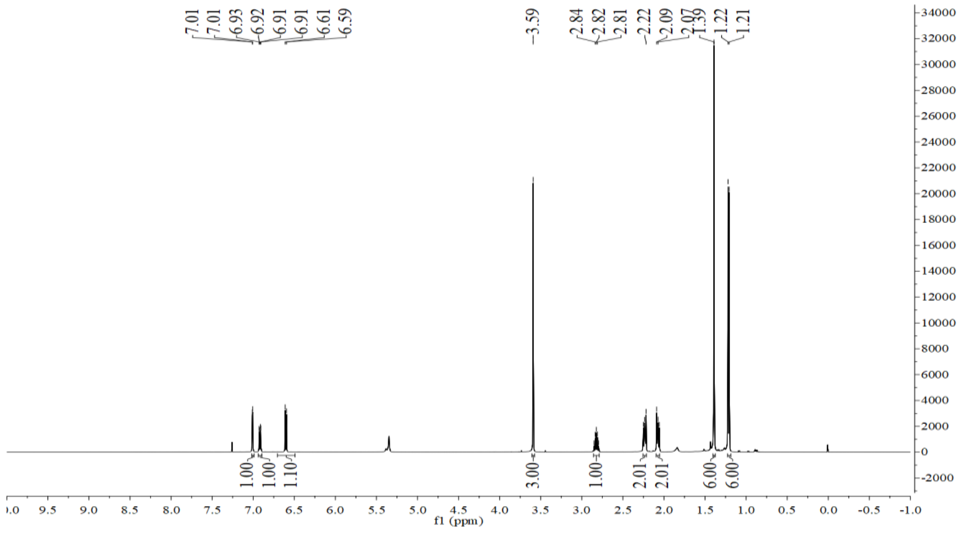

Figure S2. ^{1}H-NMR spectrum of intermediate **5**.

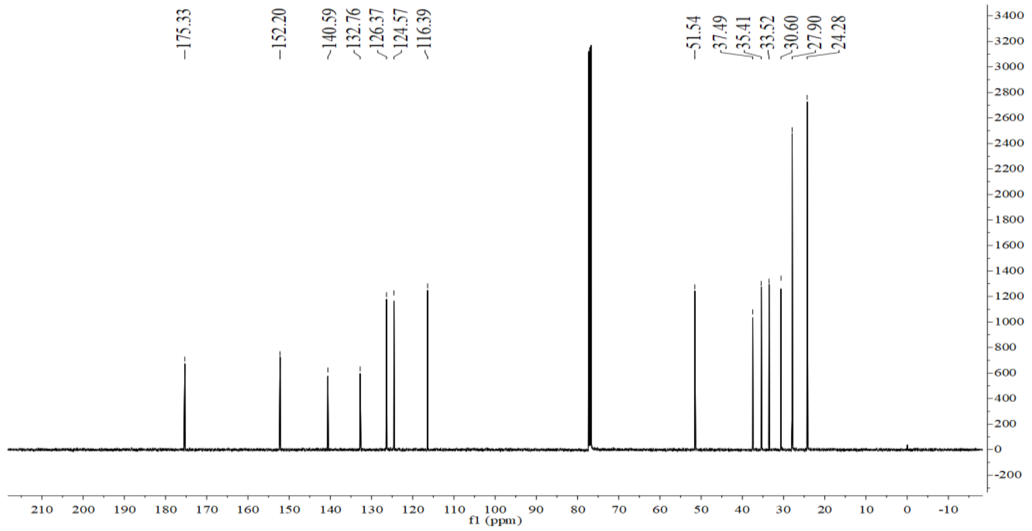

Figure S3. ^{13}C-NMR spectrum of intermediate **5**.

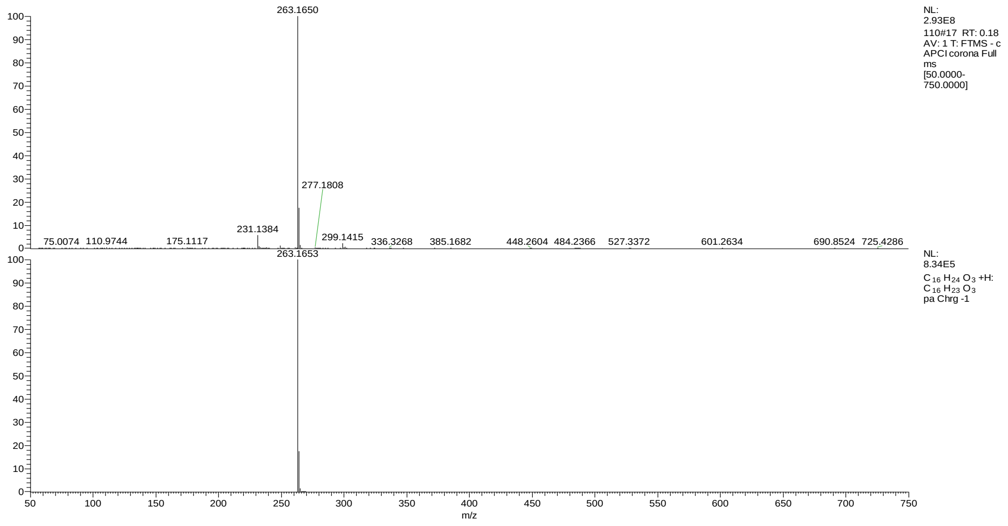

Figure S4. ESI-MS spectrum of intermediate **5**.

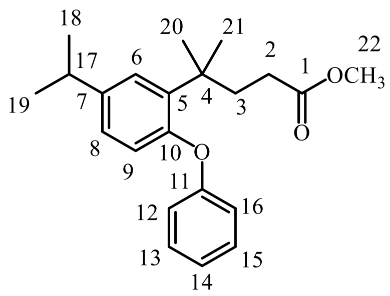

Intermediate **6a**, colorless oily liquid; yield 91.3%; FT-IR (KBr, cm^{-1}): 2958, 2926, 2872 (C-H), 1741 (C=O), 1589, 1487, 1454, 1385, 1228, 1163, 1082, 1023 (Ar-C=C); ^{1}H NMR (500 MHz, $CDCl_3$) δ 7.33 – 7.28 (m, 2H, C_{13}-H, C_{15}-H), 7.13 (d, J = 2.2 Hz, 1H, C_6-H), 7.05 (t, J = 7.9 Hz, 1H, C_{14}-H), 7.00 – 6.94 (m, 3H, C_8-H C_{12}-H, C_{16}-H), 6.73 (d, J = 8.3 Hz, 1H, C_9-H), 3.57 (s, 3H, C_{22}-H), 2.87 (dt, J = 13.8, 6.9 Hz, 1H, C_{17}-H), 2.19 (dd, J = 10.0, 7.0 Hz, 2H, C_2-H), 2.10 (dd, J = 11.1, 4.3 Hz, 2H, C_3-H), 1.40 (s, 6H, C_{20}-H, C_{21}-H), 1.24 (d, J = 6.9 Hz, 6H, C_{18}-H, C_{19}-H); ^{13}C NMR (126 MHz, $CDCl_3$) δ 174.54, 157.50, 153.42, 143.42, 137.22, 129.55, 126.50, 124.83, 122.49, 119.76, 118.50, 51.37, 37.68, 36.15, 33.70, 30.60, 28.35 , 24.16; ESI-MS m/z: 341.2103 [M + H^+].

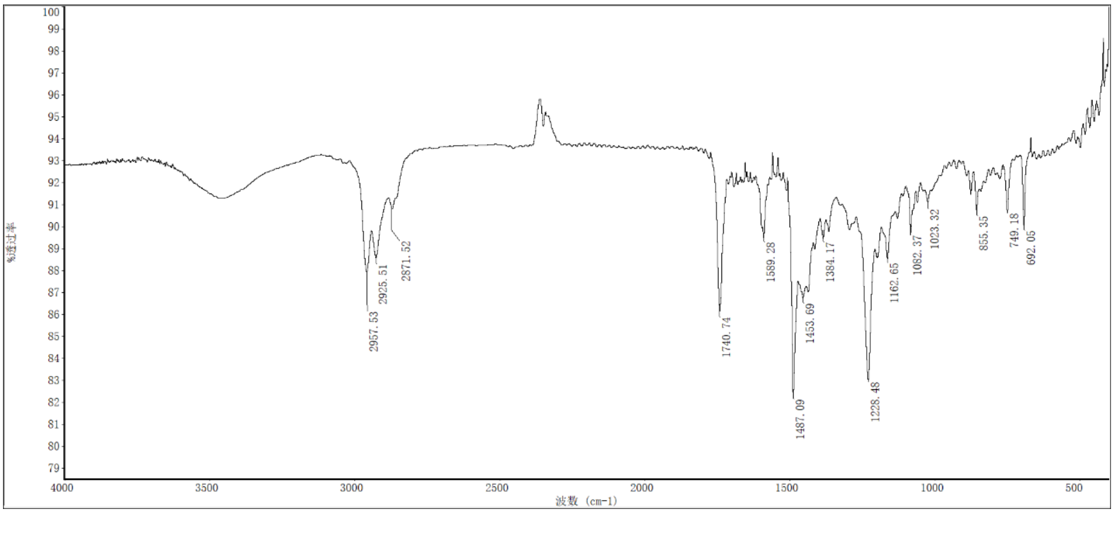

Figure S5. FT-IR spectrum of intermediate **6a**.

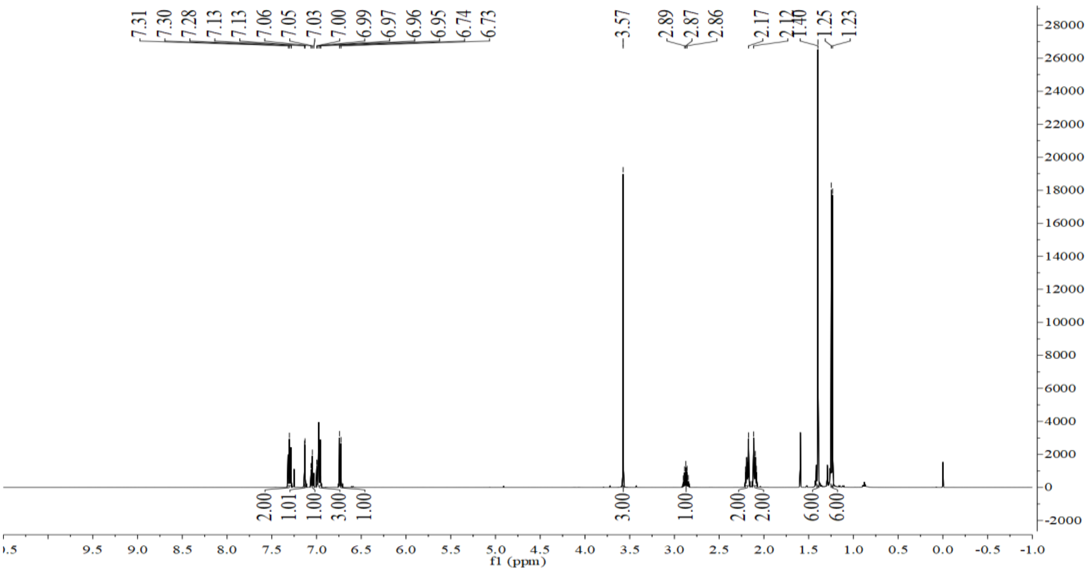

Figure S6. ^{1}H-NMR spectrum of intermediate **6a**.

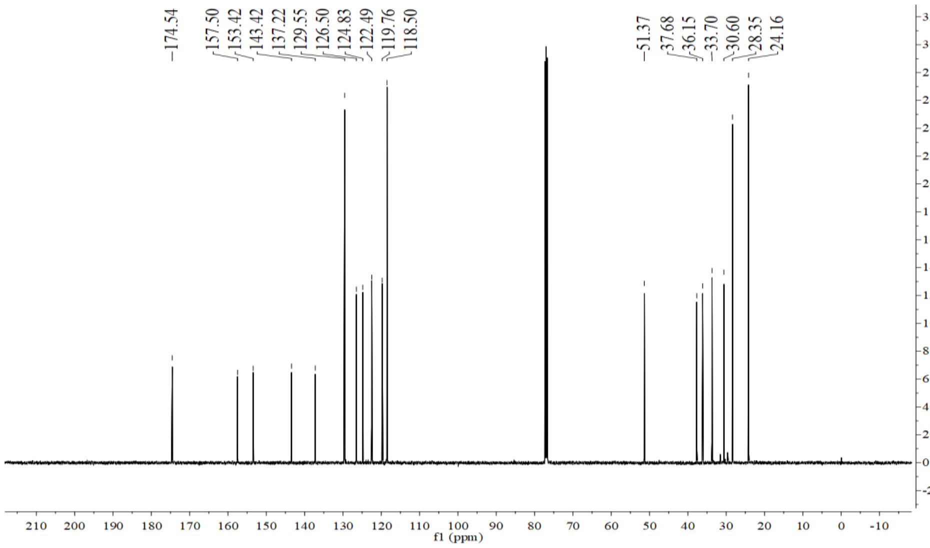

Figure S7. ^{13}C-NMR spectrum of intermediate **6a**.

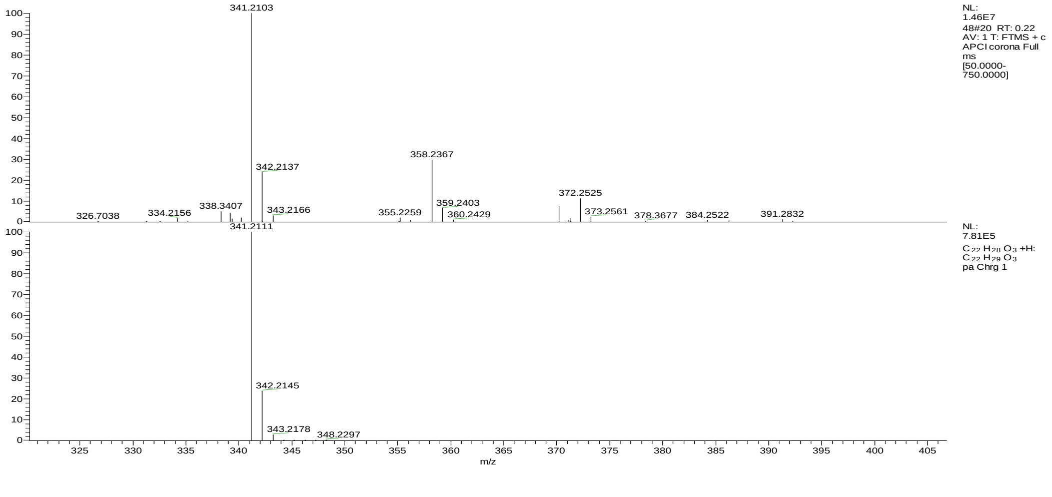

Figure S8. ESI-MS spectrum of intermediate **6a**.

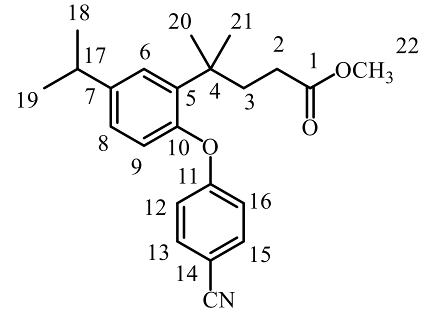

Intermediate **6b**, white solid; yield 90.5%; m. p. 76.8– 79.3 °C; FT-IR (KBr, cm^{-1}): 3451 (Ar–H), 2958, 2923, 2876 (C-H), 2223 (C≡N),1735 (C=O), 1600, 1504, 1487, 1457, 1436, 1294, 1241, 1196 (Ar-C=C); ^{1}H NMR (500 MHz, $CDCl_3$) δ 7.61 – 7.58 (m, 2H, C_{13}-H, C_{15}-H), 7.19 (d, J = 2.2 Hz, 1H, C_6-H), 7.08 (d, J = 10.4 Hz, 1H, C_8-H), 7.01 (d, J = 8.9 Hz, 2H, C_{12}-H, C_{16}-H), 6.79 (d, J = 8.2 Hz, 1H, C_9-H), 3.57 (s, 3H, C_{22}-H), 2.91 (dt, J = 13.8, 6.9 Hz, 1H, C_{17}-H), 2.09 (h, J = 5.4 Hz, 4H, C_2-H, C_3-H), 1.35 (s, 6H, C_{20}-H, C_{21}-H), 1.26 (d, J = 6.9 Hz, 6H, C_{18}-H, C_{19}-H); ^{13}C NMR (126 MHz, $CDCl_3$) δ 174.25, 161.63, 151.26, 145.46, 138.28, 134.10, 127.09, 125.37, 121.34, 118.93, 118.00, 105.43, 51.50, 37.71, 36.41, 33.85, 30.46, 28.47, 24.14; ESI-MS m/z: 364.1916 [M + H^+].

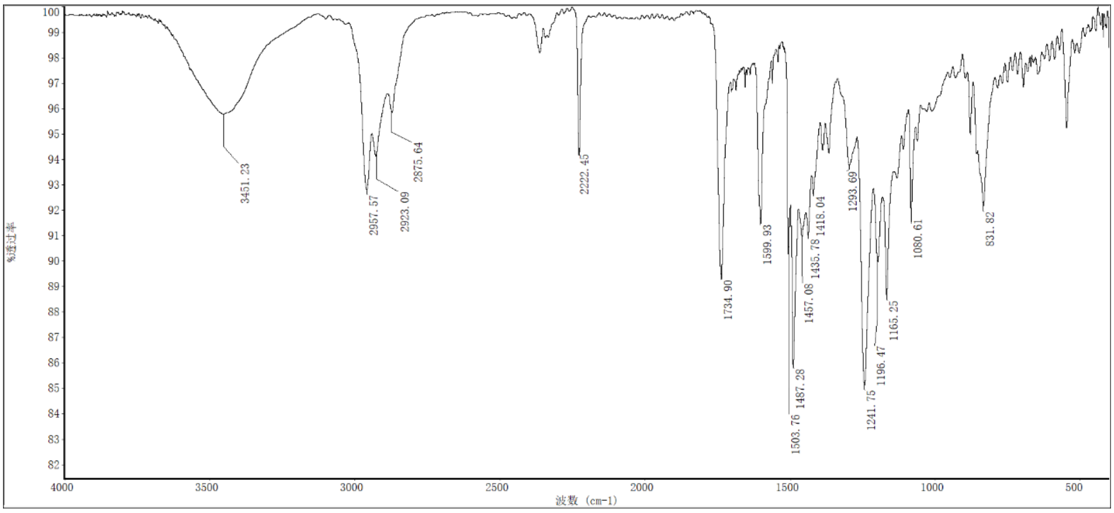

Figure S9. FT-IR spectrum of intermediate **6b**.

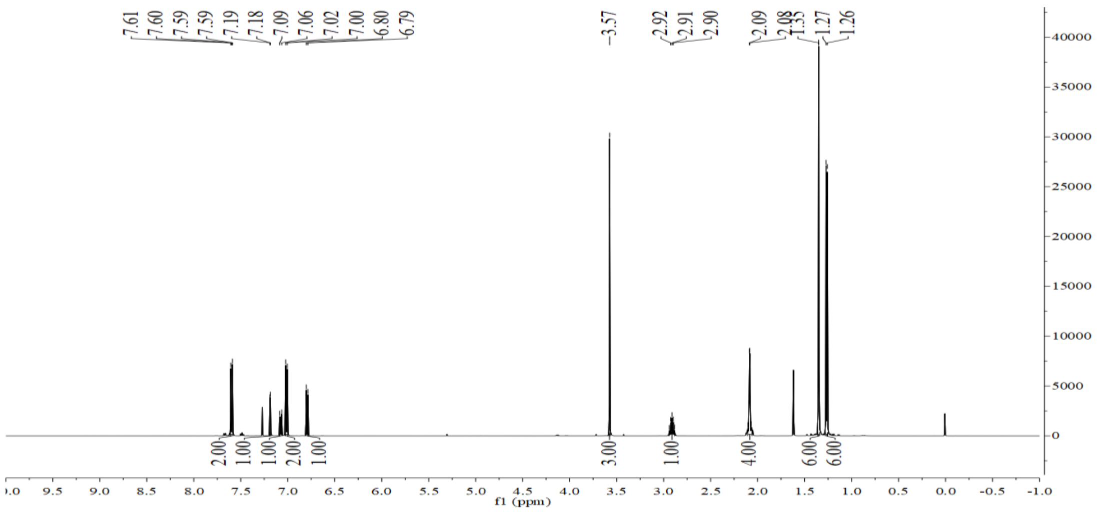

Figure S10. ^{1}H-NMR spectrum of intermediate **6b**.

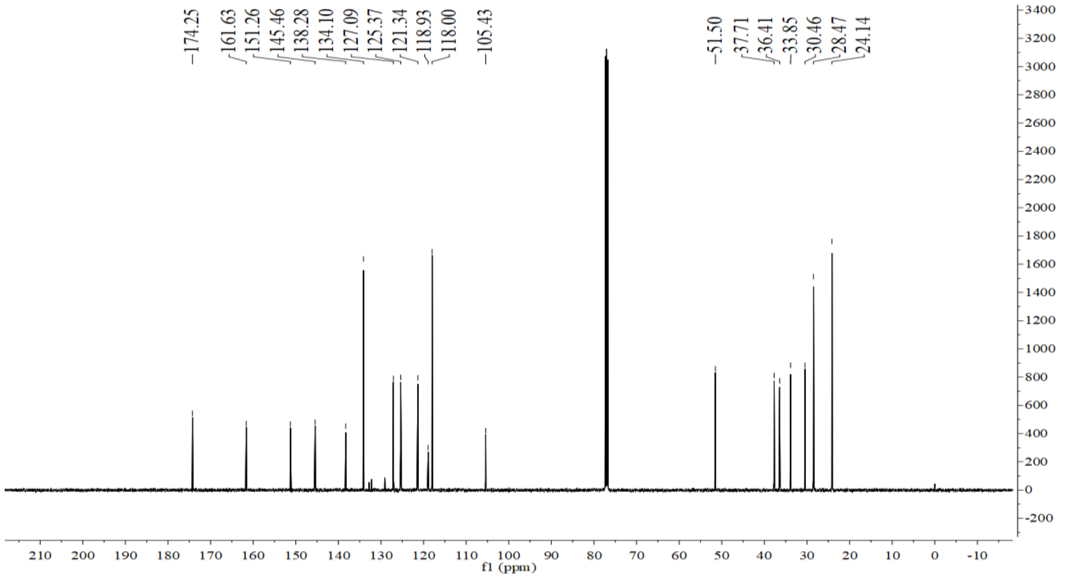

Figure S11. ^{13}C-NMR spectrum of intermediate **6b**.

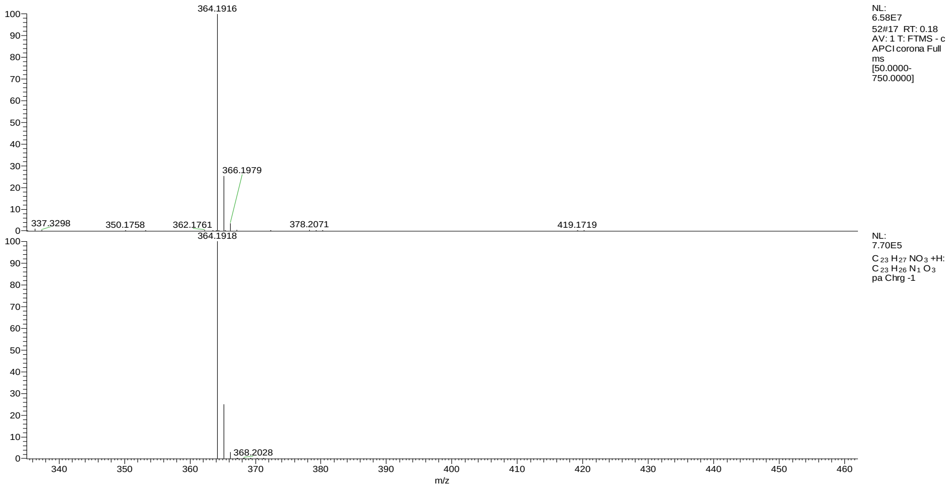

Figure S12. ESI-MS spectrum of intermediate **6b**.

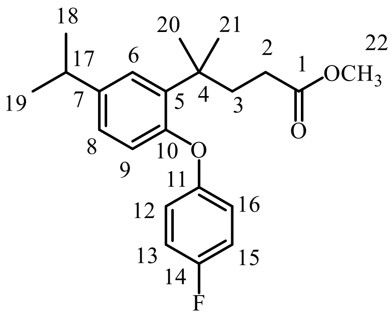

Intermediate **6c**, colorless oily liquid; yield 91.2%; FT-IR (KBr, cm^{-1}): 3452 (Ar–H), 2959, 2929, 2874 (C-H), 1739 (C=O), 1504, 1489, 1457, 1209 (Ar-C=C); ^{1}H NMR (500 MHz, $CDCl_3$) δ 7.13 (d, J = 2.2 Hz, 1H, C_6-H), 7.02 – 6.97 (m, 3H, C_8-H, C_{13}-H, C_{15}-H), 6.95 – 6.89 (m, 2H, C_{12}-H, C_{16}-H), 6.67 (d, J = 8.3 Hz, 1H, C_9-H), 3.58 (s, 3H, C_{22}-H), 2.87 (dt, J = 13.8, 6.9 Hz, 1H, C_{17}-H), 2.19 (dd, J = 10.3, 5.4 Hz, 2H, C_2-H), 2.11 – 2.07 (m, 2H, C_3-H), 1.40 (s, 6H, C_{20}-H, C_{21}-H), 1.23 (d, J = 6.9 Hz, 6H, C_{18}-H, C_{19}-H); ^{13}C NMR (126 MHz, $CDCl_3$) δ 174.55, 159.39, 157.48, 153.93, 153.29 (d, J = 2.4 Hz), 143.44, 136.96, 126.62, 124.94, 120.00 (d, J = 8.2 Hz), 119.09, 116.23, 116.05, 51.44, 37.74, 36.18, 33.73, 30.65, 28.35, 24.20; ESI-MS m/z: 359.2007 [M + H^+].

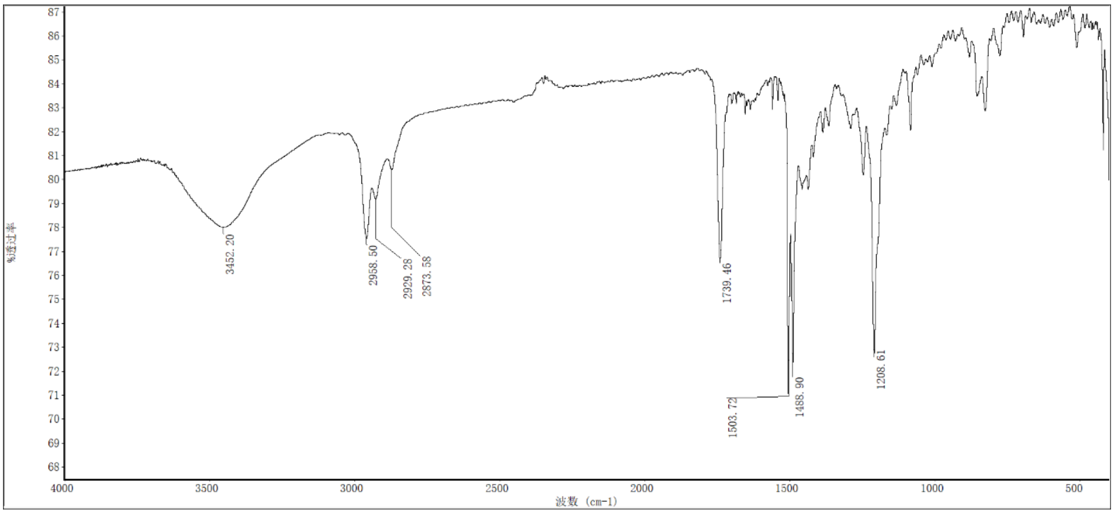

Figure S13. FT-IR spectrum of intermediate **6c**.

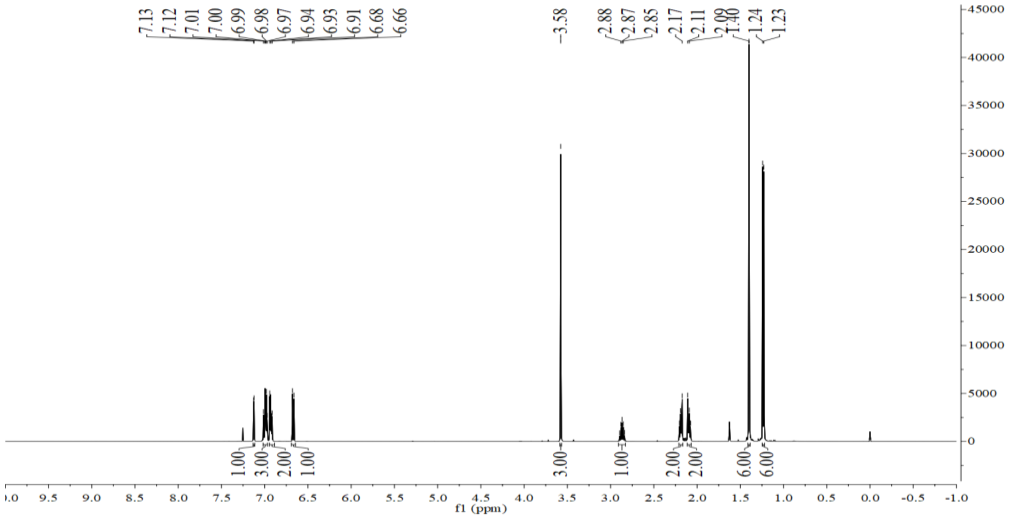

Figure S14. ^{1}H-NMR spectrum of intermediate **6c**.

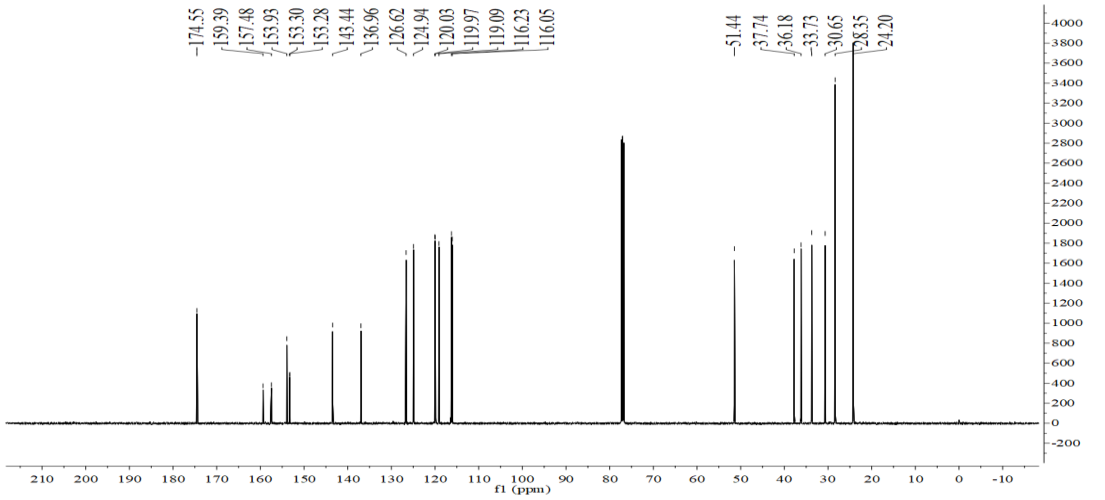

Figure S15. ^{13}C-NMR spectrum of intermediate **6c**.

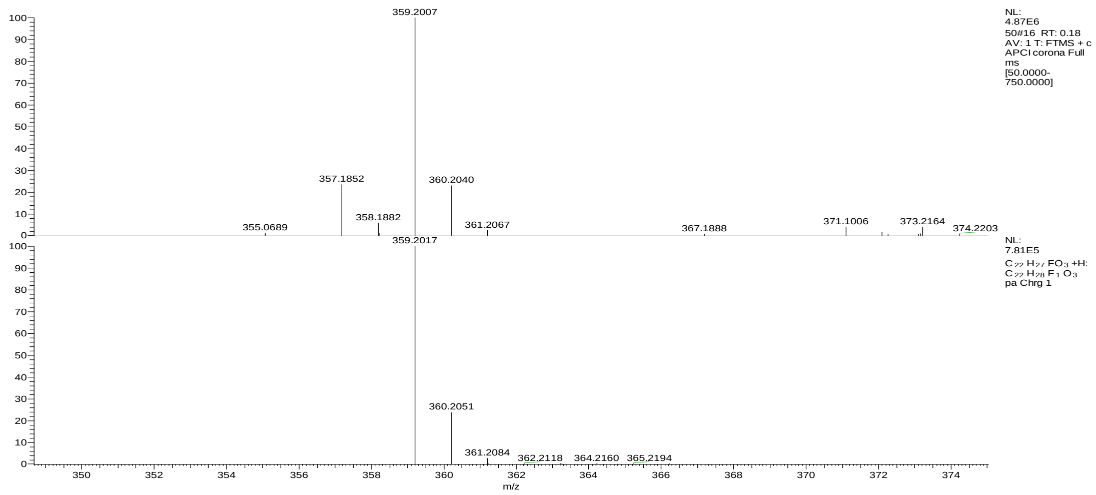

Figure S16. ESI-MS spectrum of intermediate **6c**.

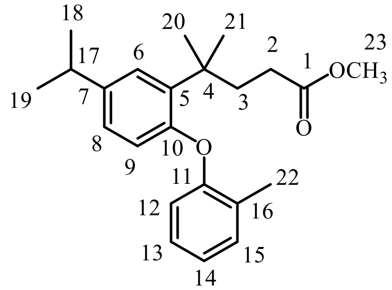

Intermediate **6d**, colorless oily liquid, white solid; yield 93.2%; FT-IR (KBr, cm^{-1}): 2957, 2927, 2867 (C-H), 1740 (C=O), 1486, 1456, 1385, 1234, 1206, 1184 (Ar-C=C); ^1H NMR (500 MHz, $CDCl_3$) δ 7.26 – 7.23 (m, 1H, C_6-H), 7.14 – 7.10 (m, 2H, C_{13}-H, C_{15}-H), 7.01 (td, J = 7.4, 1.0 Hz, 1H, C_{14}-H), 6.95 (dd, J = 8.3, 2.2 Hz, 1H, C_8-H), 6.81 (d, J = 7.5 Hz, 1H, C_9-H), 6.56 (d, J = 8.3 Hz, 1H, C_{12}-H), 3.58 (s, 3H, C_{23}-H), 2.87 (dt, J = 13.8, 6.9 Hz, 1H, C_{17}-H), 2.30 (s, 3H, C_{22}-H), 2.26 (dd, J = 10.4, 6.3 Hz, 2H, C_2-H), 2.13 (dd, J = 9.6, 7.3 Hz, 2H, C_3-H), 1.44 (s, 6H, C_{20}-H, C_{21}-H), 1.24 (d, J = 6.9 Hz, 6H, C_{18}-H, C_{19}-H); ^{13}C NMR (126 MHz, $CDCl_3$) δ 174.61, 154.93, 154.02, 142.60, 136.17, 131.18, 129.62, 127.03, 126.53, 124.76, 123.12, 118.59, 117.65, 51.38, 37.80, 36.18, 33.67, 30.75, 28.36, 24.22, 16.34; ESI-MS m/z: 355.2259 [M + H^+].

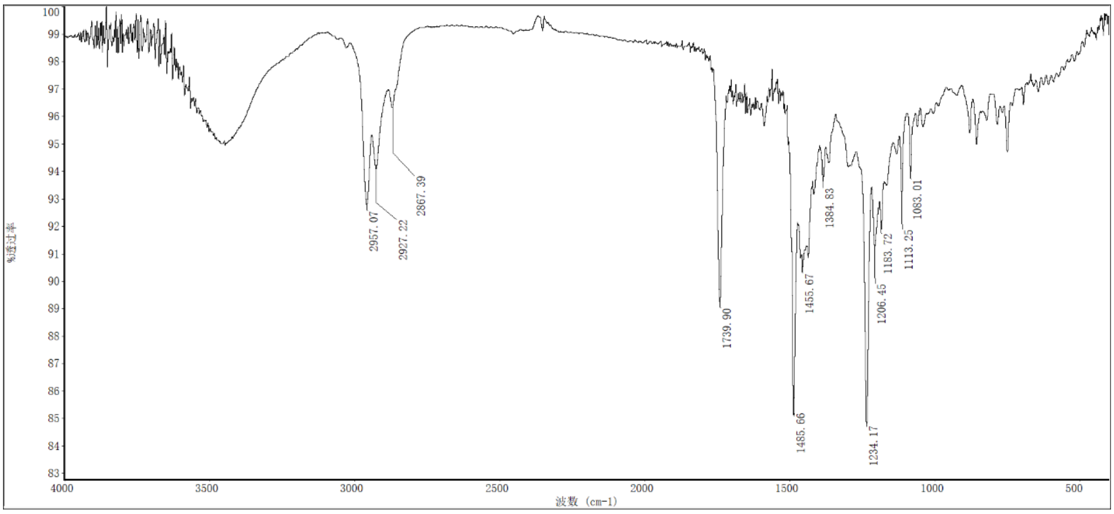

Figure S17. FT-IR spectrum of intermediate **6d**.

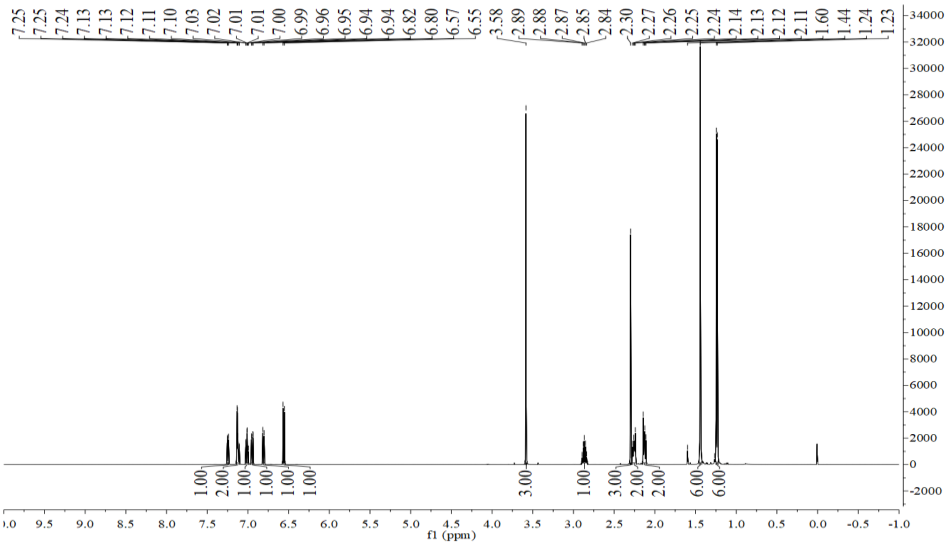

Figure S18. ^{1}H-NMR spectrum of intermediate **6d**.

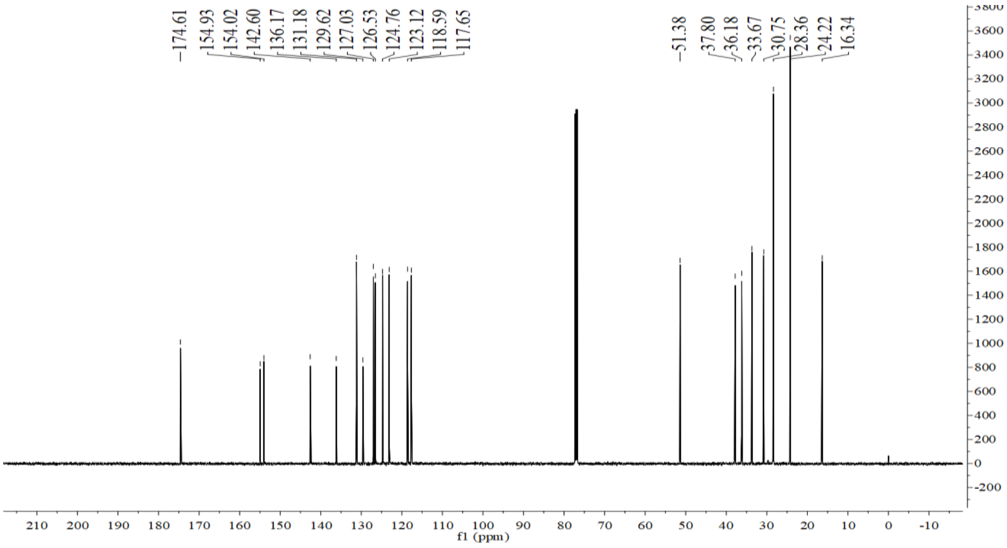

Figure S19. ^{13}C-NMR spectrum of intermediate **6d**.

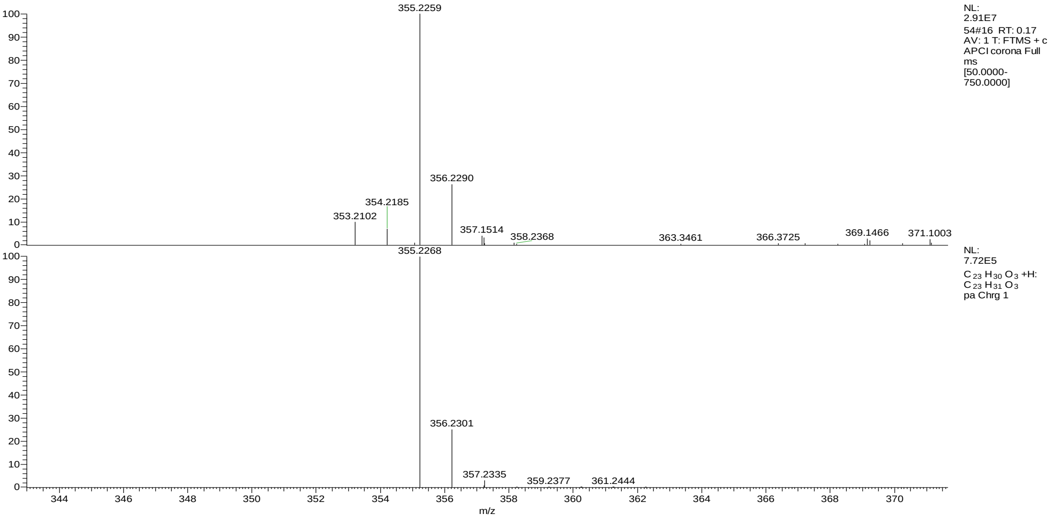

Figure S20. ESI-MS spectrum of intermediate **6d**.

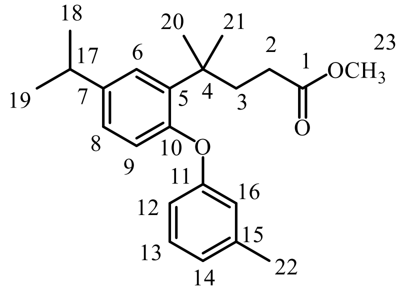

Intermediate **6e**, colorless oily liquid; yield 97.8s%;; FT-IR (KBr, cm^{-1}): 2958, 2925, 2865 (C-H), 1741 (C=O), 1609, 1582, 1485, 1458, 1435, 1255, 1212 (Ar-C=C); ^{1}H NMR (500 MHz, $CDCl_3$) δ 7.18 (t, *J* = 7.8 Hz, 1H, C_6-H), 7.13 (d, *J* = 2.2 Hz, 1H, C_8-H), 6.99 (dd, *J* = 8.3, 2.2 Hz, 1H, C_9-H), 6.87 (d, *J* = 7.5 Hz, 1H, C_{16}-H), 6.81 (s, 1H, C_6-H, C_{12}-H), 6.74 (t, *J* = 9.1 Hz, 2H, C_{13}-H, C_{14}-H), 3.58 (s, 3H, C_{23}-H), 2.87 (dd, *J* = 13.8, 6.9 Hz, 1H, C_{17}-H), 2.32 (s, 3H, C_{22}-H), 2.23 – 2.18 (m, 2H, C_2-H), 2.13 – 2.08 (m, 2H, C_3-H), 1.40 (s, 6H, C_{20}-H, C_{21}-H), 1.25 (d, *J* = 6.9 Hz, 6H, C_{18}-H, C_{19}-H); ^{13}C NMR (126 MHz, $CDCl_3$) δ 174.61, 157.47, 153.61, 143.30, 139.78, 137.15, 129.32, 126.51, 124.85, 123.43, 119.71, 119.34, 115.58, 51.41, 37.73, 36.19, 33.75, 30.68, 28.41, 24.23, 21.42; ESI-MS m/z: 355.2258 [M + H^+].

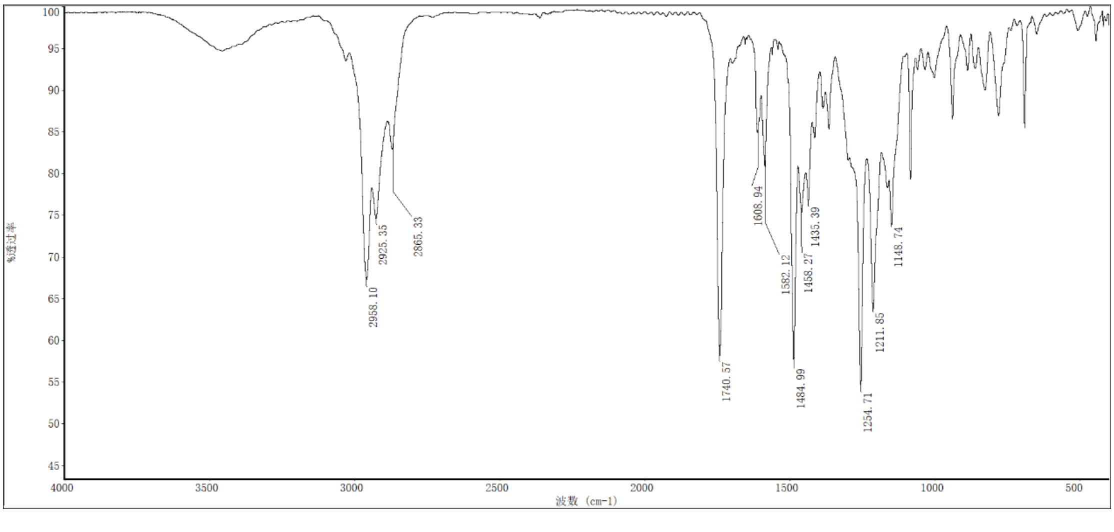

Figure S21. FT-IR spectrum of intermediate **6e**.

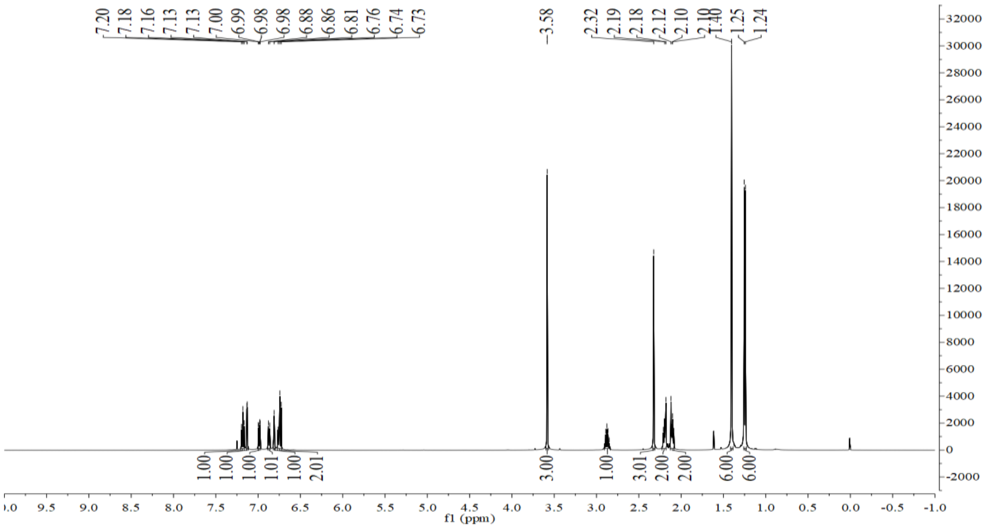

Figure S22. ^{1}H-NMR spectrum of intermediate **6e**.

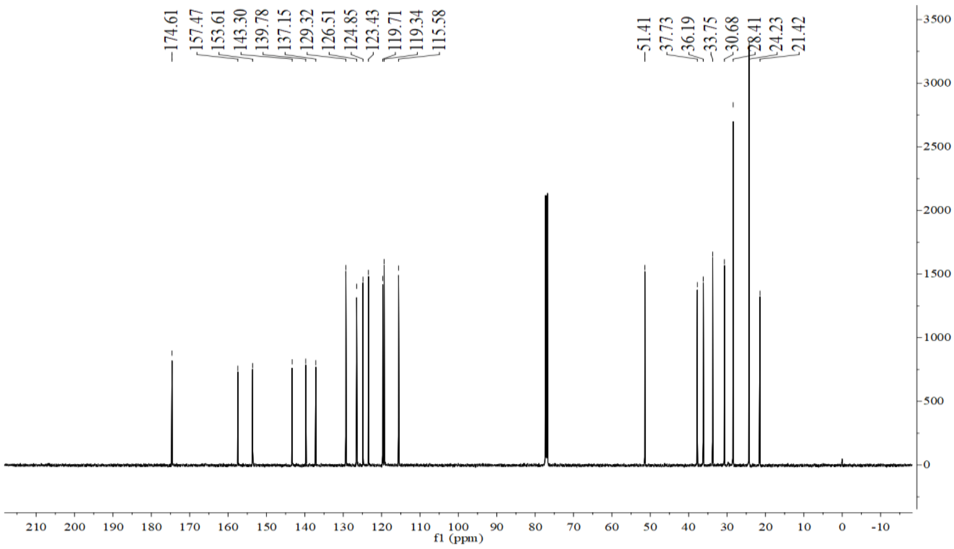

Figure S23. ^{13}C-NMR spectrum of intermediate **6e**.

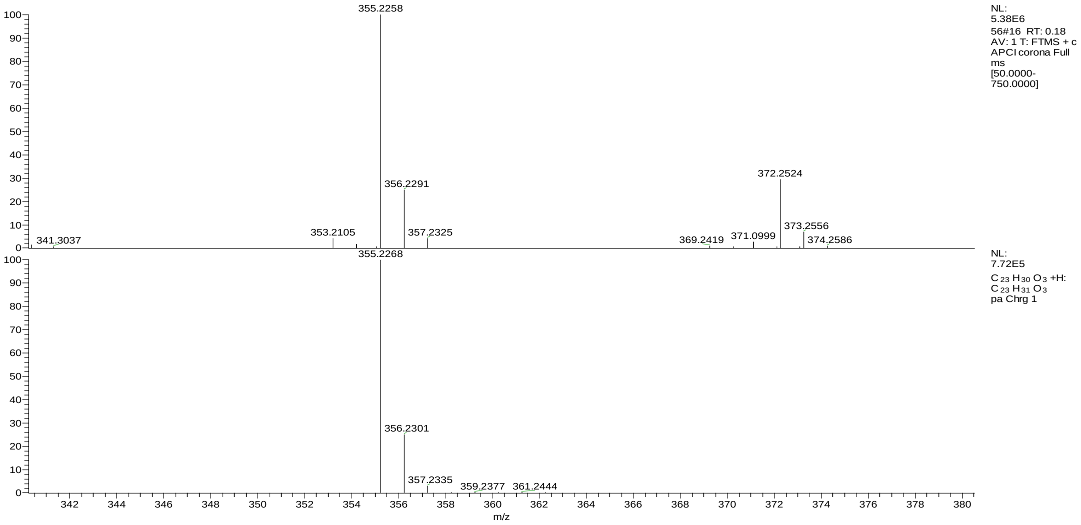

Figure S24. ESI-MS spectrum of intermediate **6e**.

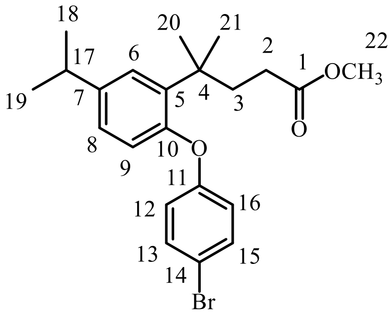

Intermediate **6f**, yellowish oily liquid; yield 95.4%; FT-IR (KBr, cm^{-1}): 2959, 2929, 2871(C-H), 1740(C=O), 1582, 1481, 1435, 1415, 1228, 1234, 1198, 1164, 1082 (Ar-C=C); ^{1}H NMR (500 MHz, $CDCl_3$) δ 7.43 – 7.36 (m, 2H, C_{13}-H, C_{15}-H), 7.14 (d, J = 2.2 Hz, 1H, C_6-H), 7.02 – 6.99 (m, 1H, C_8-H), 6.87 – 6.83 (m, 2H, C_{12}-H, C_{16}-H), 6.73 (d, J = 8.3 Hz, 1H, C_9-H), 3.57 (s, 3H, C_{22}-H), 2.88 (dd, J = 13.8, 6.9 Hz, 1H, C_{17}-H), 2.17 – 2.12 (m, 2H, C_2-H), 2.10 – 2.06 (m, 2H, C_3-H), 1.37 (s, 6H, C_{20}-H, C_{21}-H), 1.24 (d, J = 6.9 Hz, 6H, C_{18}-H, C_{19}-H); ^{13}C NMR (126 MHz, $CDCl_3$) δ 174.44, 156.86, 152.89, 144.09, 137.52, 132.54, 126.72, 125.05, 120.03 (d, J = 8.1 Hz), 114.84, 51.45, 37.71, 36.25, 33.76, 30.59, 28.40, 24.17; ESI-MS m/z: 419.1205 [M + H^+].

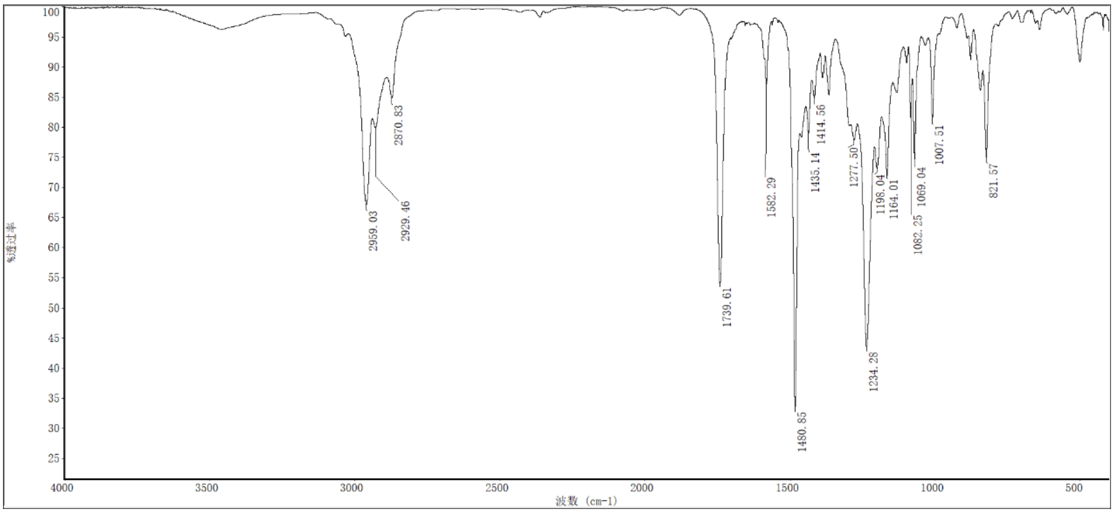

Figure S25. FT-IR spectrum of intermediate **6f**.

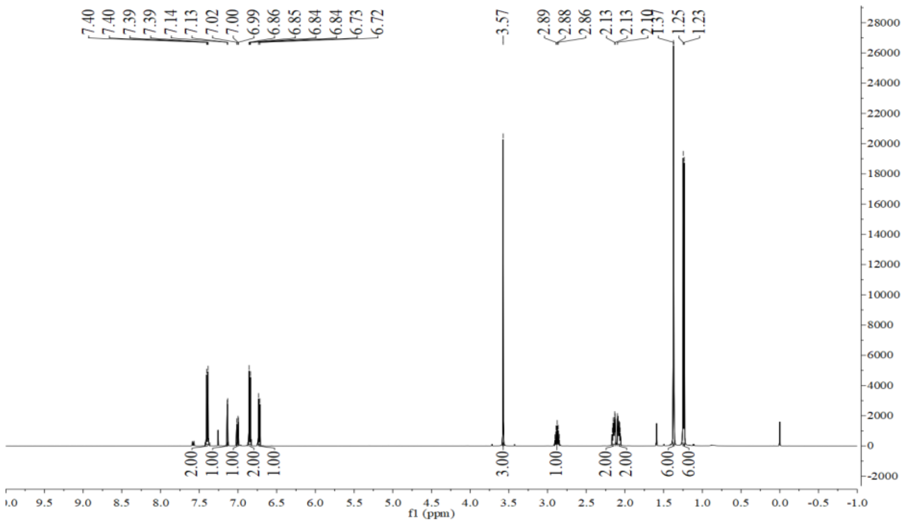

Figure S26. ^{1}H-NMR spectrum of intermediate **6f**.

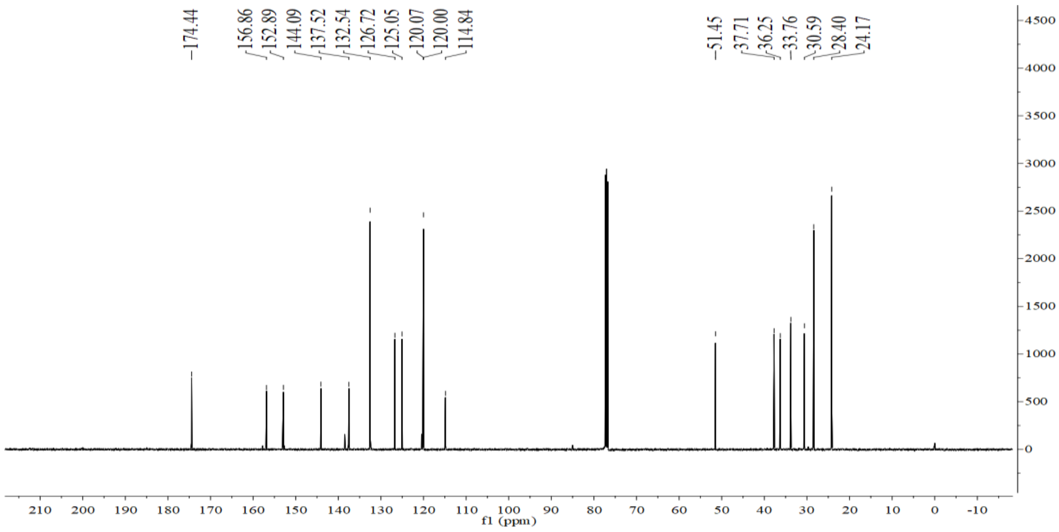

Figure S27. ^{13}C-NMR spectrum of intermediate **6f**.

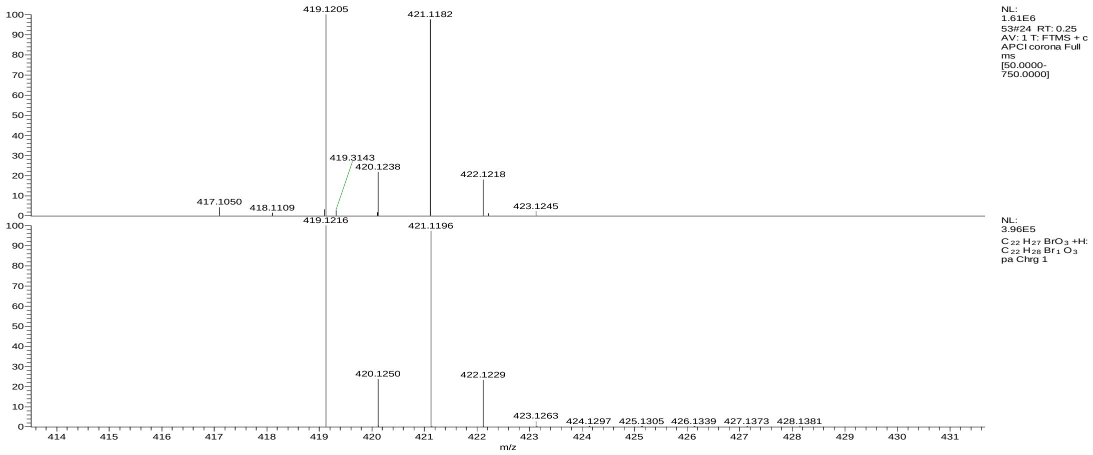

Figure S28. ESI-MS spectrum of intermediate **6f**.

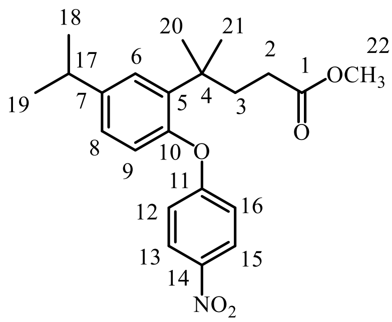

Intermediate **6g**, white solid; yield 96.1%; m. p. 83.8– 86.5 °C; FT-IR (KBr, cm^{-1}): 2958, 2926, 2870 (C-H), 1738 (C=O), 1610, 1589, 1519, 1486, 1365, 1343, 1246, 1196, 1165, 1111 (Ar-C=C); ^{1}H NMR (500 MHz, $CDCl_3$) δ 8.20 (d, *J* = 7.3 Hz, 2H, C_{13}-H, C_{15}-H), 7.20 (d, *J* = 2.0 Hz, 1H, C_6-H), 7.09 (dd, *J* = 8.2, 2.0 Hz, 1H, C_8-H), 7.02 (d, *J* = 9.2 Hz, 2H, C_{12}-H, C_{16}-H), 6.82 (d, *J* = 8.2 Hz, 1H, C_9-H), 3.56 (s, 3H, C_{22}-H), 2.92 (dt, *J* = 13.8, 6.9 Hz, 1H, C_{17}-H), 2.09 (s, 4H, C_2-H, C_3-H), 1.35 (s, 6H, C_{20}-H, C_{21}-H), 1.27 (d, *J* = 6.9 Hz, 6H, C_{18}-H, C_{19}-H); ^{13}C NMR (126 MHz, $CDCl_3$) δ 174.21, 163.33, 151.21, 145.76, 142.44, 138.35, 127.17, 125.96, 125.48, 121.51, 117.20, 51.50, 37.72, 36.44, 33.87, 30.44, 28.49, 24.13; ESI-MS m/z: 385.1892 [M + H^+].

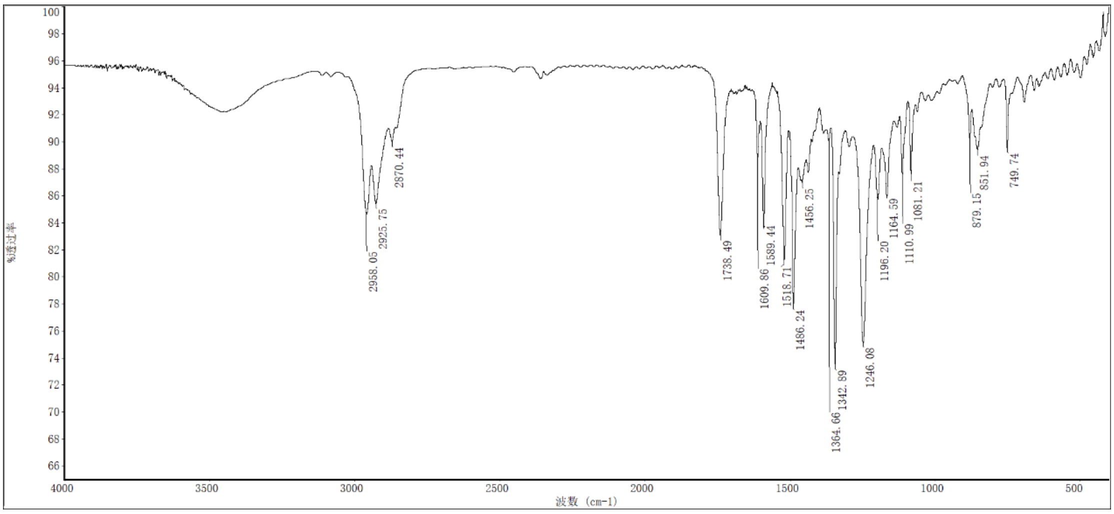

Figure S29. FT-IR spectrum of intermediate **6g**.

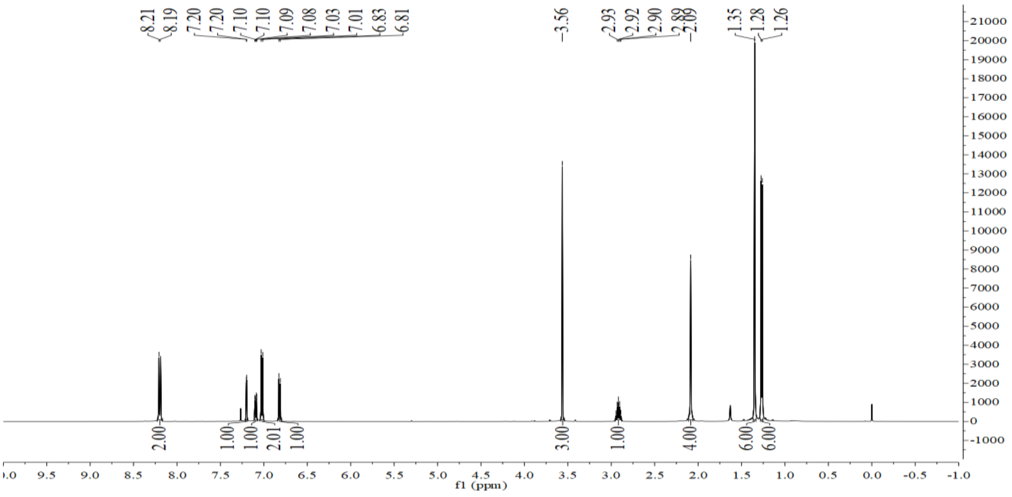

Figure S30. ^{1}H-NMR spectrum of intermediate **6g**.

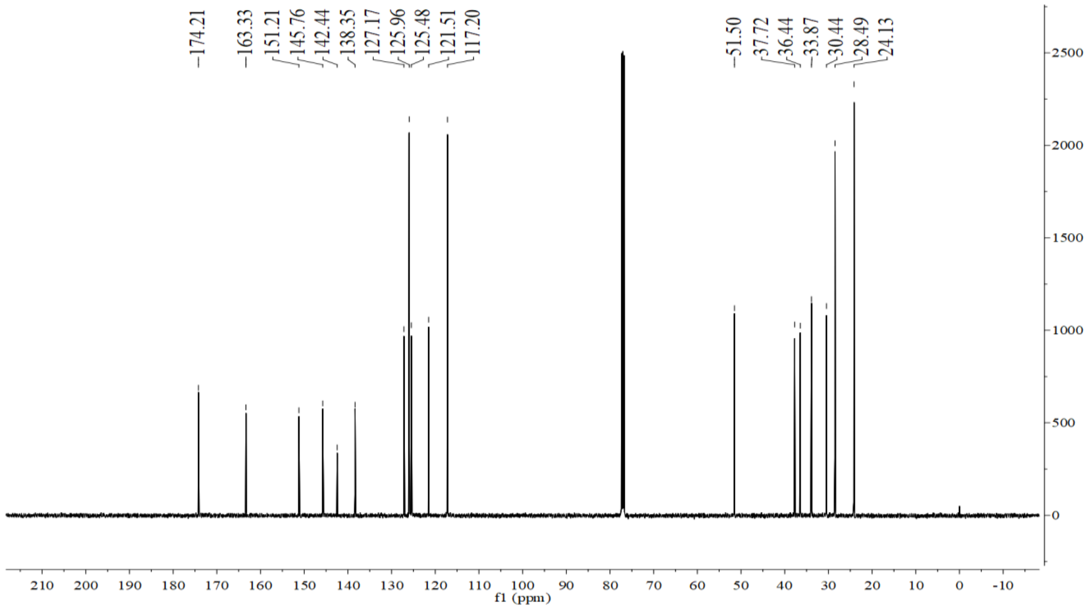

Figure S31. ^{13}C-NMR spectrum of intermediate **6g**.

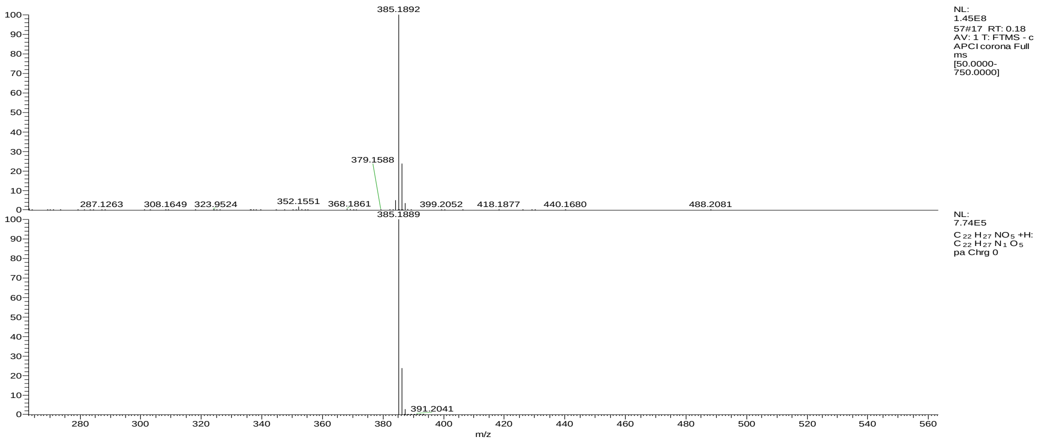

Figure S32. ESI-MS spectrum of intermediate **6g**.

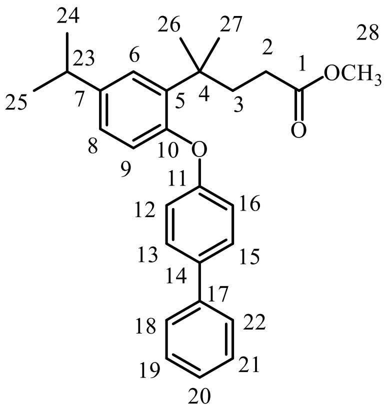

Intermediate **6h**, colorless oily liquid, white solid; yield 94.8%; FT-IR (KBr, cm^{-1}): 3445 (Ar–H), 2957, 2925, 2869 (C-H), 1738 (C=O), 1601, 1515, 1483, 1456, 1435, 1234, 1167, 1082 (Ar-C=C); ^{1}H NMR (500 MHz, $CDCl_3$) δ 7.54 (dd, J = 14.7, 8.6 Hz, 4H, C_{13}-H, C_{15}-H, C_{18}-H, C_{22}-H), 7.42 (t, J = 7.7 Hz, 2H, C_{19}-H, C_{21}-H), 7.31 (t, J = 7.4 Hz, 1H, C_{20}-H), 7.15 (d, J = 2.2 Hz, 1H, C_6-H), 7.02 (dd, J = 13.5, 5.4 Hz, 3H, C_8-H, C_{12}-H, C_{16}-H), 6.81 (d, J = 8.3 Hz, 1H, C_9-H), 3.57 (s, 3H, C_{28}-H), 2.89 (dt, J = 13.8, 6.9 Hz, 1H, C_{23}-H), 2.23 – 2.19 (m, 2H, C_2-H), 2.14 – 2.09 (m, 2H, C_3-H), 1.41 (s, 6H, C_{26}-H, C_{27}-H), 1.25 (d, J = 6.9 Hz, 6H, C_{24}-H, C_{25}-H); ^{13}C NMR (126 MHz, $CDCl_3$) δ 174.47, 157.06, 153.28, 143.56, 140.55, 137.28, 135.50, 128.64, 128.21, 126.76 (d, J = 6.5 Hz), 126.51, 124.85, 119.89, 118.61, 51.34, 37.65, 36.15, 33.67, 30.57, 28.33, 24.11; ESI-MS m/z: 417.2413 [M + H^+].

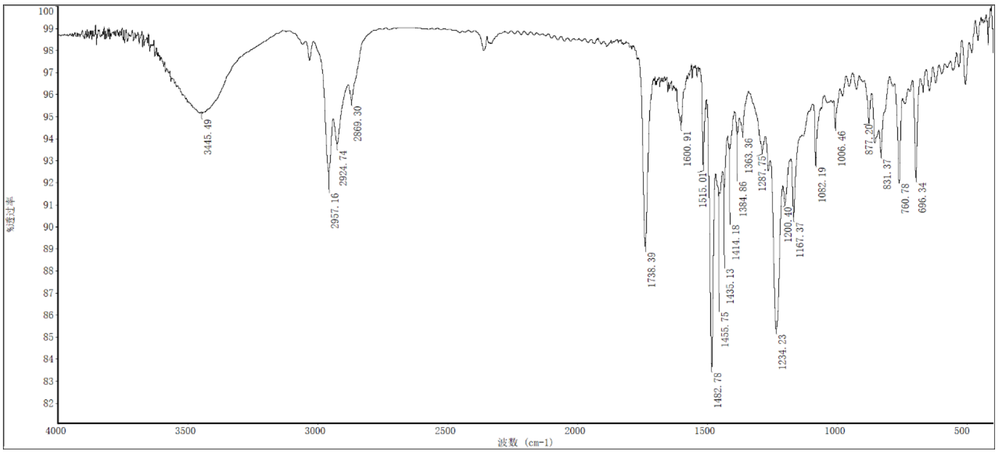

Figure S33. FT-IR spectrum of intermediate **6h**.

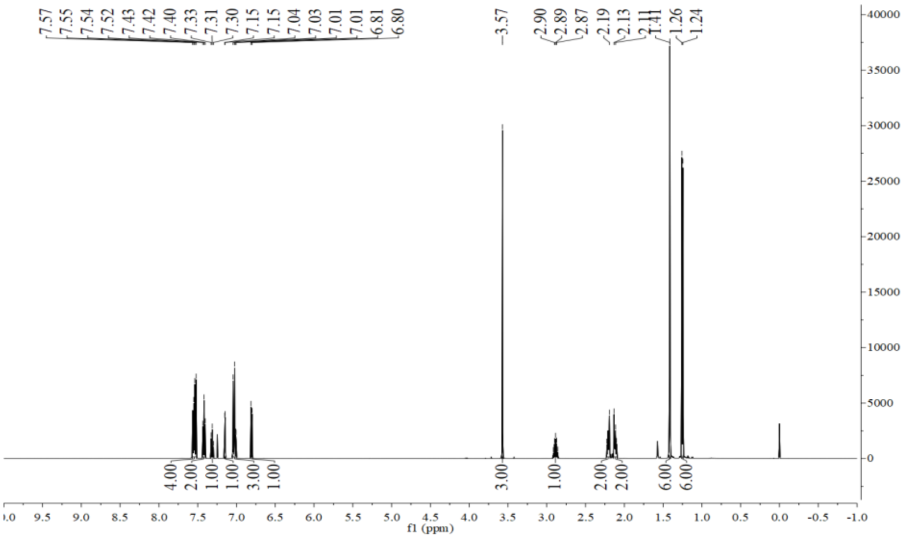

Figure S34. ^{1}H-NMR spectrum of intermediate **6h**.

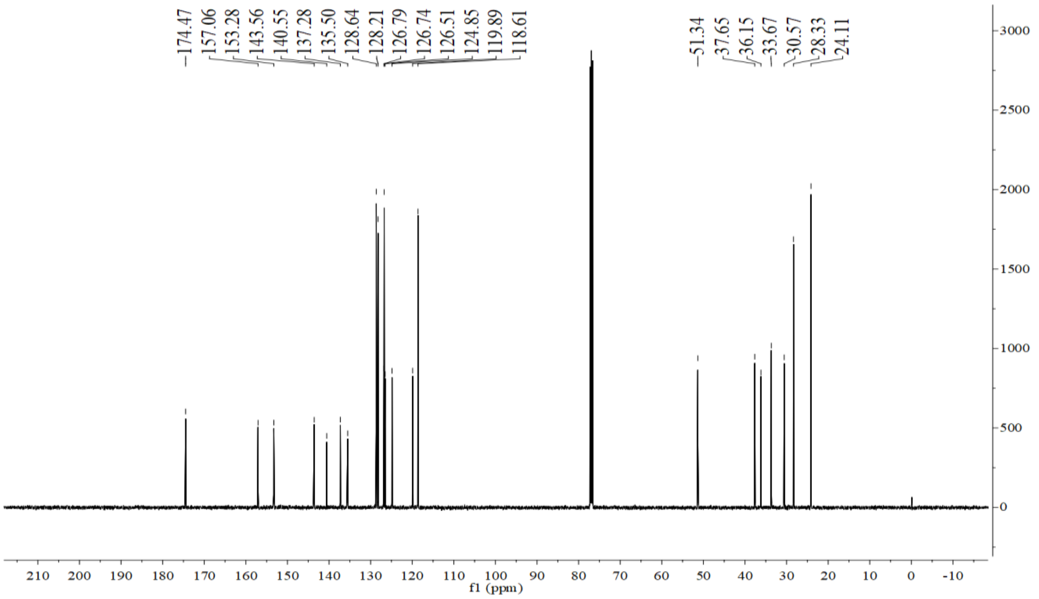

Figure S35. ^{13}C-NMR spectrum of intermediate **6h**.

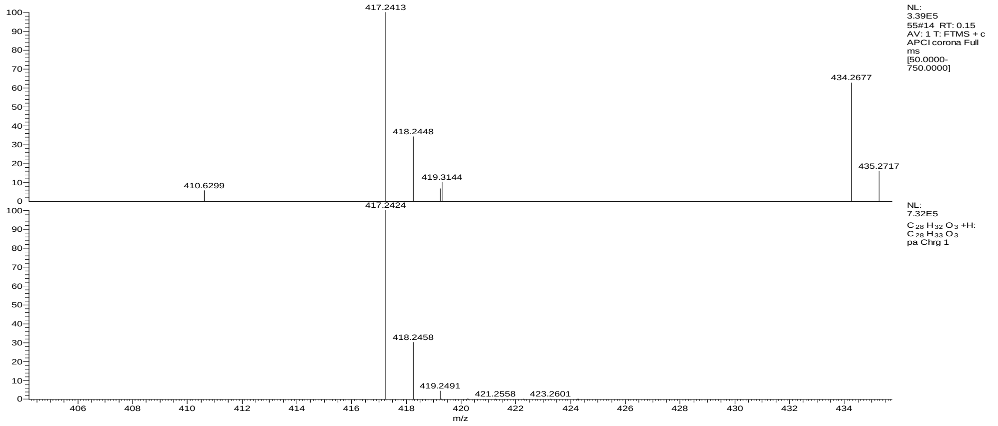

Figure S36. ESI-MS spectrum of intermediate **6h**.

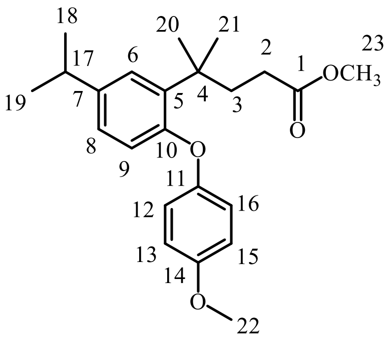

Intermediate **6i**, yellowish oily liquid; yield 87.9%; FT-IR (KBr, cm^{-1}): 3451 (Ar–H), 2957, 2926, 2872 (C-H), 1739 (C=O), 1506, 1489, 1440, 1296, 1221, 1180, 1083, 1037 (Ar-C=C); ^{1}H NMR (600 MHz, $CDCl_3$) δ 7.10 (s, 1H, C_6-H), 6.93 (dd, *J* = 14.8, 8.1 Hz, 3H, C_8-H, C_{13}-H, C_{15}-H), 6.86 (d, *J* = 9.1 Hz, 2H, C_{12}-H, C_{16}-H), 6.64 (d, *J* = 8.3 Hz, 1H, C_9-H), 3.79 (s, 3H, C_{22}-H), 3.58 (s, 3H, C_{23}-H), 2.85 (dt, *J* = 13.8, 6.9 Hz, 1H, C_{17}-H), 2.24 – 2.20 (m, 2H, C_2-H), 2.13 – 2.09 (m, 2H, C_3-H), 1.42 (s, 6H, C_{20}-H, C_{21}-H), 1.23 (d, *J* = 6.9 Hz, 6H, C_{18}-H, C_{19}-H); ^{13}C NMR (151 MHz, $CDCl_3$) δ 174.67, 155.39, 154.76, 150.71, 142.68, 136.43, 126.44, 124.76, 120.30, 118.29, 114.79, 55.65, 51.42, 37.74, 36.09, 33.68, 30.71, 28.32, 24.22; ESI-MS m/z: 371.2208 [M + H^+].

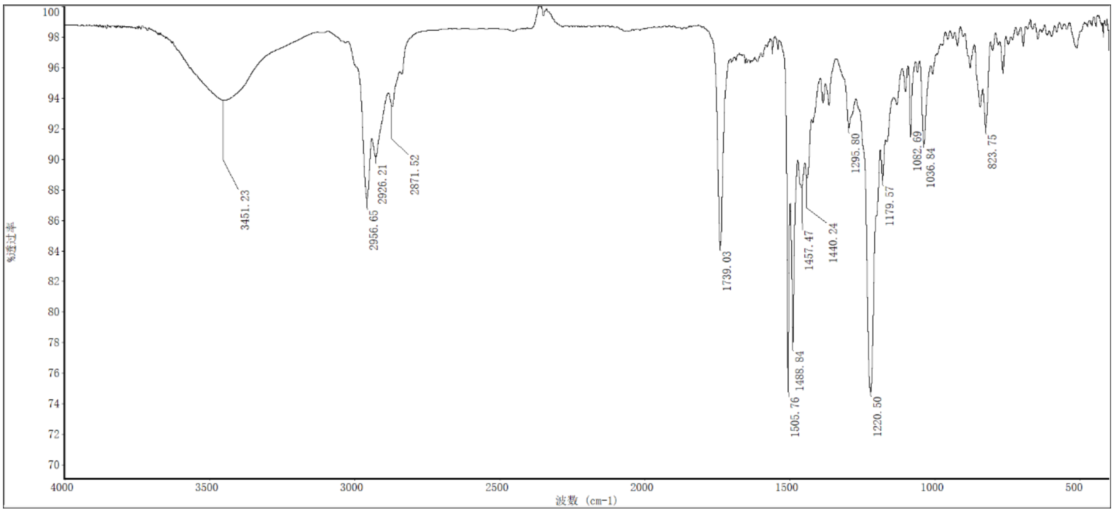

Figure S37. FT-IR spectrum of intermediate **6i**.

Figure S38. ^{1}H-NMR spectrum of intermediate **6i**.

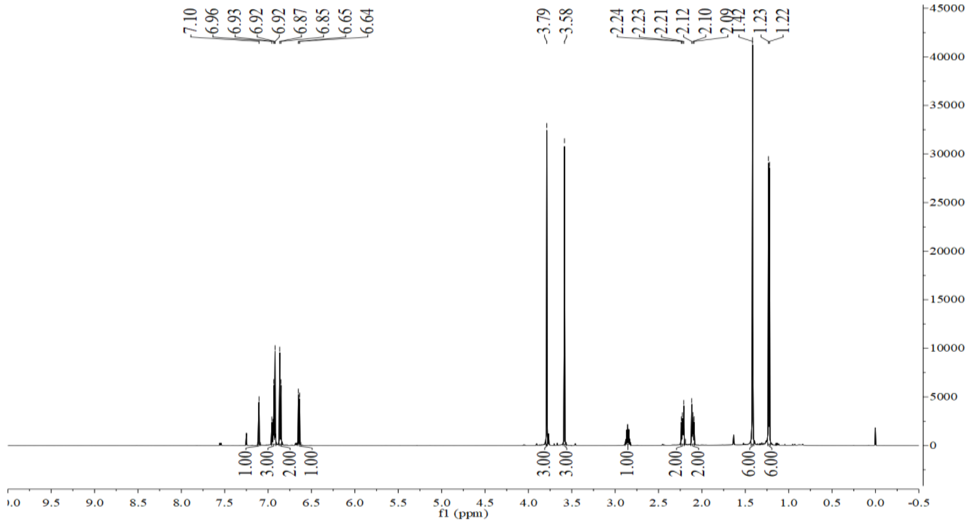

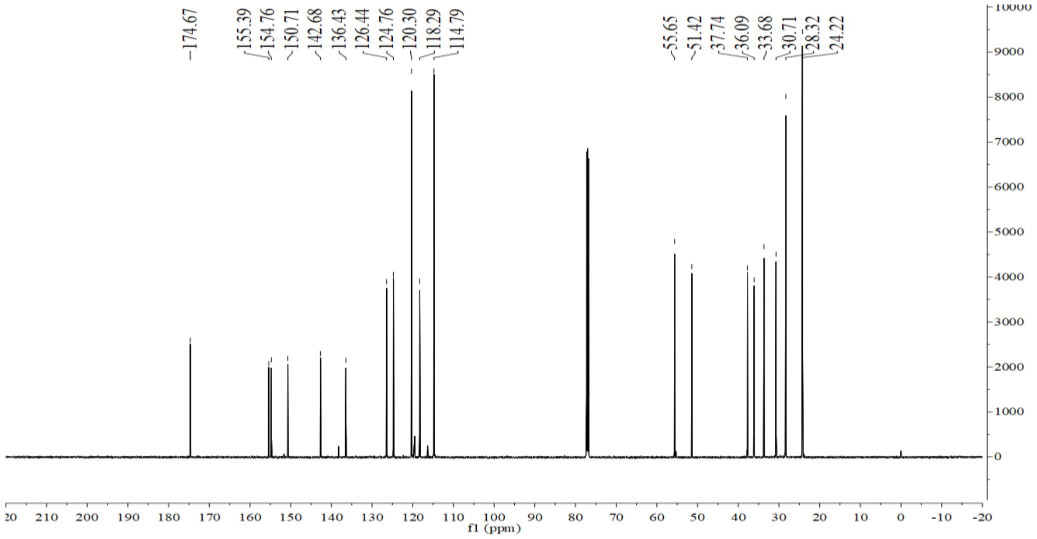

Figure S39. ^{13}C-NMR spectrum of intermediate **6i**.

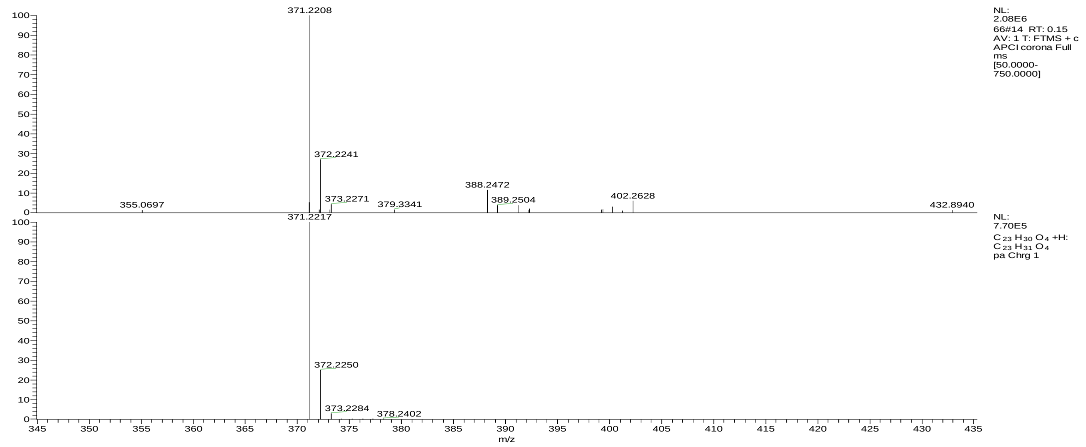

Figure S40. ESI-MS spectrum of intermediate **6i**.

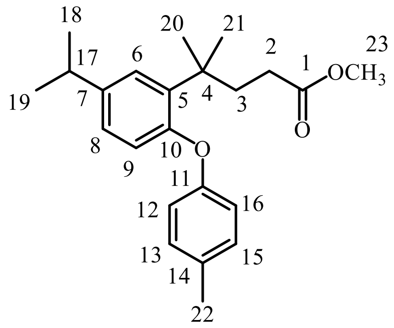

Intermediate **6j**, colorless oily liquid; yield 95.8%; FT-IR (KBr, cm^{-1}): 2958, 2926, 2869 (C-H), 1741 (C=O), 1507, 1489, 1457, 1435, 1288, 1232, 1166, 1083 (Ar-C=C); ^{1}H NMR (500 MHz, $CDCl_3$) δ 7.12 (t, J = 5.3 Hz, 3H, C_6-H, C_{13}-H, C_{15}-H), 6.98 (d, J = 2.2 Hz, 1H, C_8-H), 6.87 (d, J = 8.5 Hz, 2H, C_{12}-H, C_{16}-H), 6.71 (d, J = 8.3 Hz, 1H, C_9-H), 3.59 (s, 3H, C_{23}-H), 2.87 (dt, J = 13.8, 6.9 Hz, 1H, C_{17}-H), 2.32 (s, 3H, C_{22}-H), 2.20 (dd, J = 10.3, 5.5 Hz, 2H, C_2-H), 2.11 (dd, J = 9.4, 6.5 Hz, 2H, C_3-H), 1.41 (s, 6H, C_{20}-H, C_{21}-H), 1.24 (d, J = 6.9 Hz, 6H, C_{18}-H, C_{19}-H); ^{13}C NMR (126 MHz, $CDCl_3$) δ 174.64, 155.16, 153.98, 143.07, 136.95, 132.05, 130.10, 126.45, 124.80, 119.25, 118.67, 51.40, 37.72, 36.15, 33.72, 30.67, 28.36, 24.21, 20.64; ESI-MS m/z: 355.2258 [M + H^+].

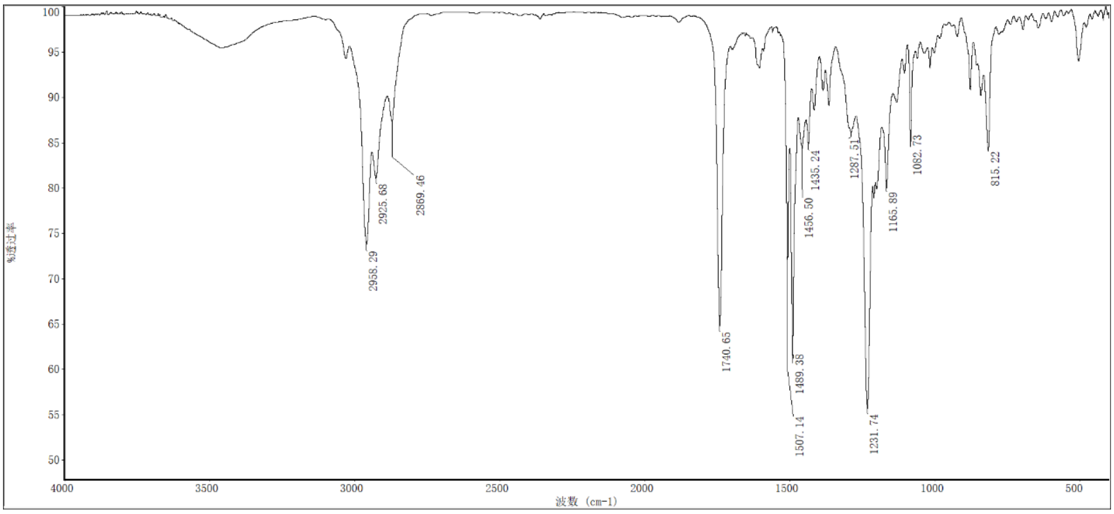

Figure S41. FT-IR spectrum of intermediate **6j**.

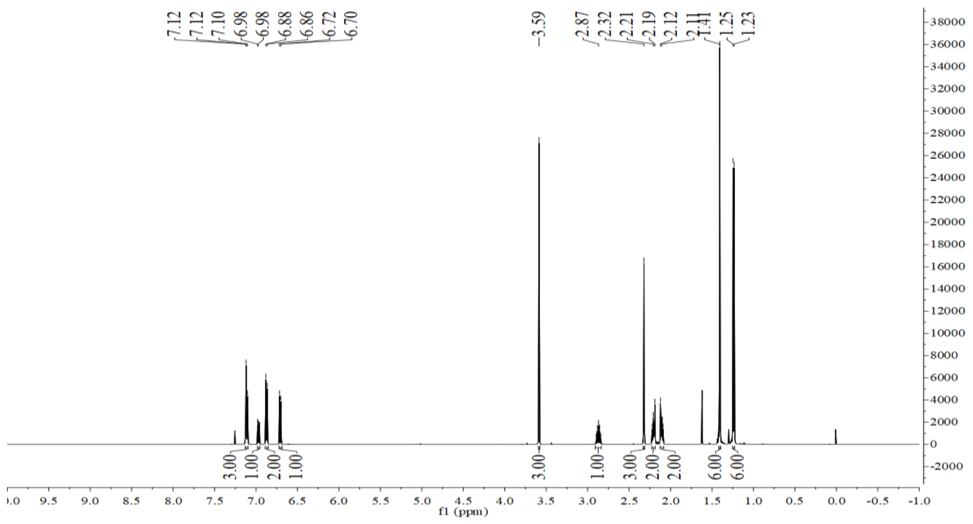

Figure S42. ^{1}H-NMR spectrum of intermediate **6j**.

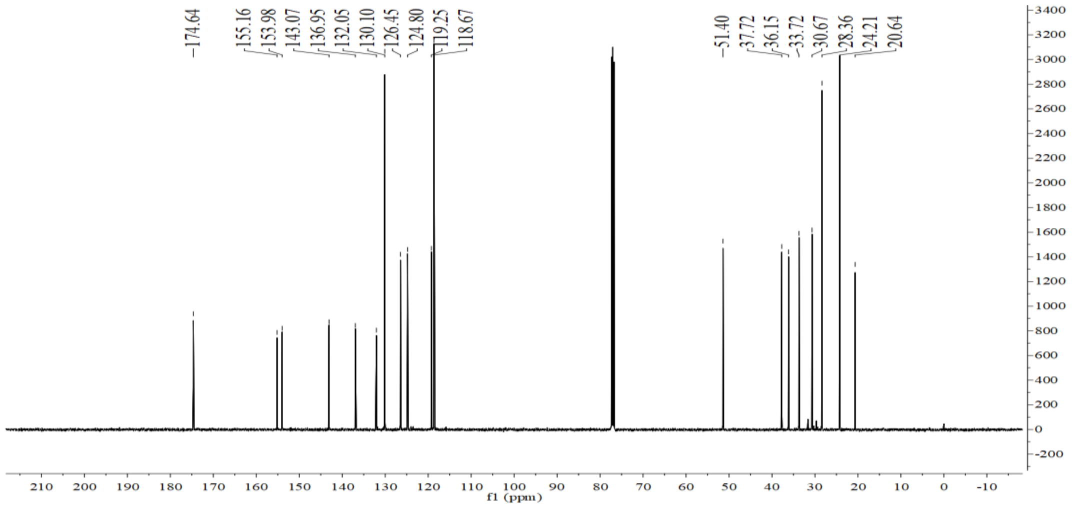

Figure S43. ^{13}C-NMR spectrum of intermediate **6j**.

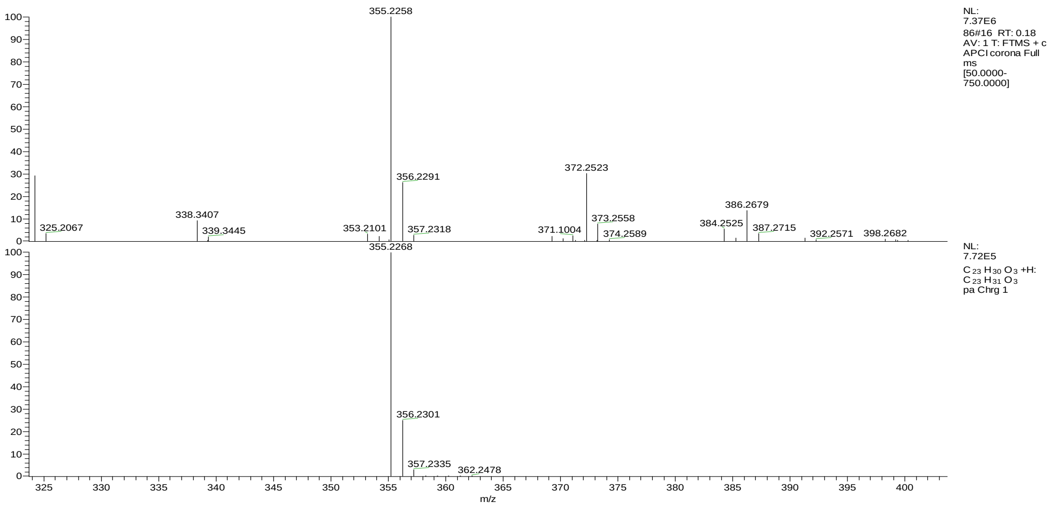

Figure S44. ESI-MS spectrum of intermediate **6j**.

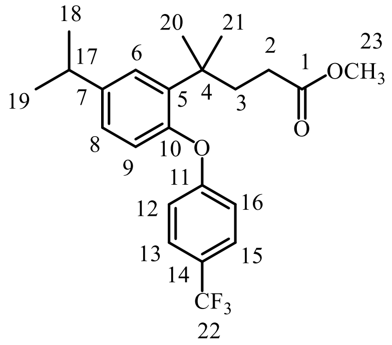

Intermediate **6k**, colorless oily liquid; yield 87.7%; FT-IR (KBr, cm^{-1}): 2959, 2927, 2872 (C-H), 1740 (C=O), 1616, 1514, 1489, 1456, 1324, 1243, 1123, 1083, 1065 (Ar-C=C); ^{1}H NMR (500 MHz, $CDCl_3$) δ 7.55 (d, J = 8.6 Hz, 2H, C_{13}-H, C_{15}-H), 7.17 (d, J = 2.2 Hz, 1H, C_6-H), 7.04 (dd, J = 16.0, 5.4 Hz, 3H, C_8-H, C_{12}-H, C_{16}-H), 6.78 (d, J = 4.7 Hz, 1H, C_9-H), 3.56 (s, 3H, C_{23}-H), 2.90 (dt, J = 13.8, 6.9 Hz, 1H, C_{17}-H), 2.10 (dd, J = 23.0, 9.7 Hz, 4H, C_2-H, C_3-H), 1.37 (s, 6H, C_{20}-H, C_{21}-H), 1.26 (d, J = 6.9 Hz, 6H, C_{18}-H, C_{19}-H); ^{13}C NMR (126 MHz, $CDCl_3$) δ 174.36, 160.63, 152.04, 144.86, 138.05, 127.42 – 126.79, 125.24, 120.99, 117.69, 51.44, 37.74, 36.38, 33.84, 30.56, 28.47, 24.17; ESI-MS m/z: 307.1836 [M + H^+].

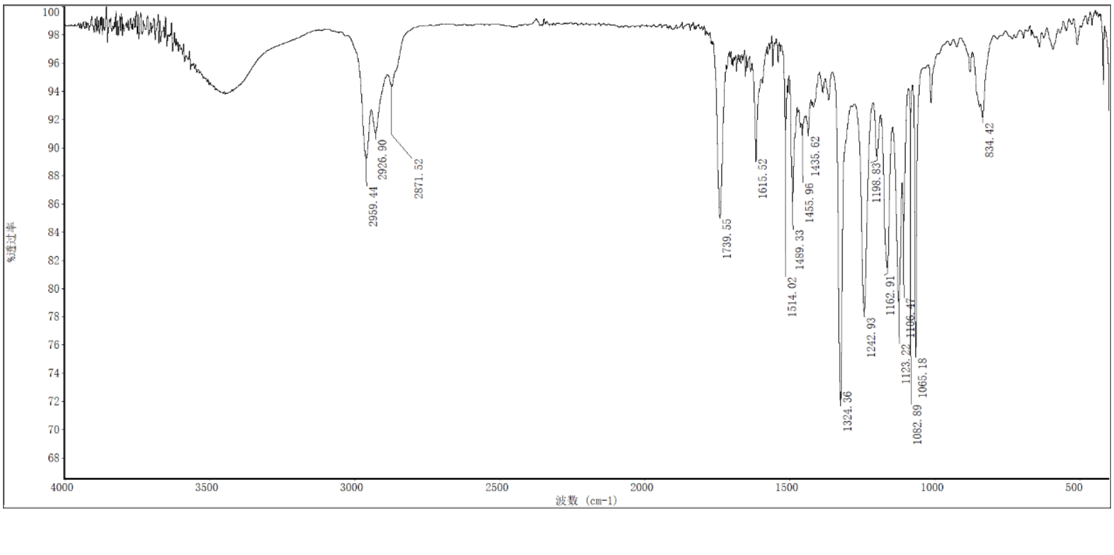

Figure S45. FT-IR spectrum of intermediate **6k**.

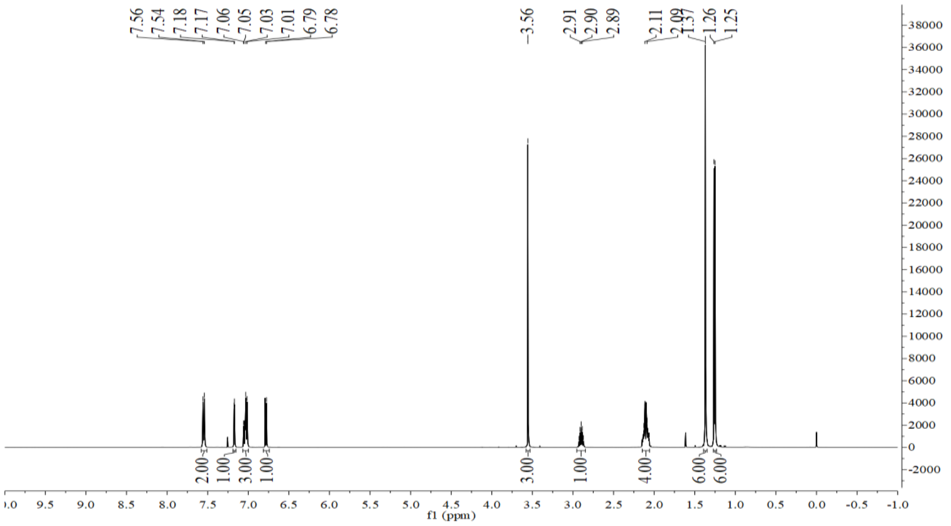

Figure S46. ^{1}H-NMR spectrum of intermediate **6k**.

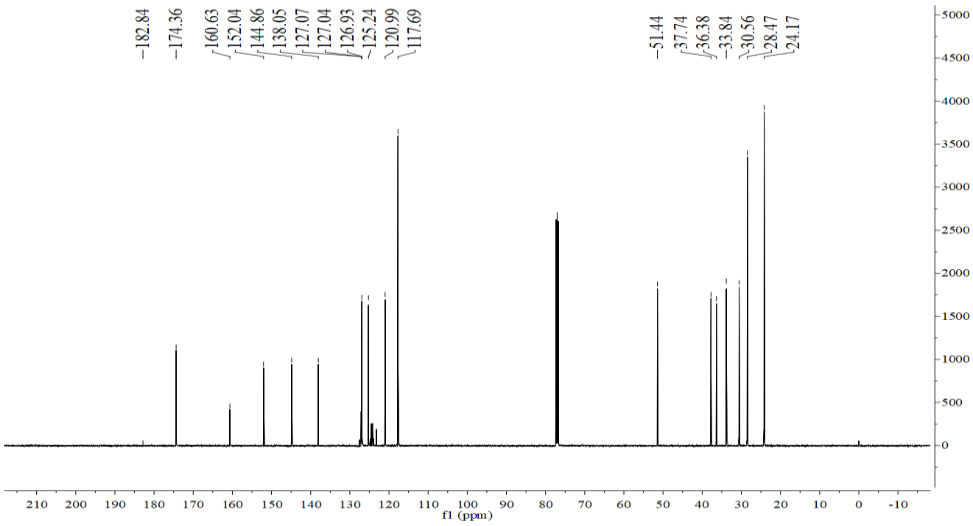

Figure S47. ^{13}C-NMR spectrum of intermediate **6k**.

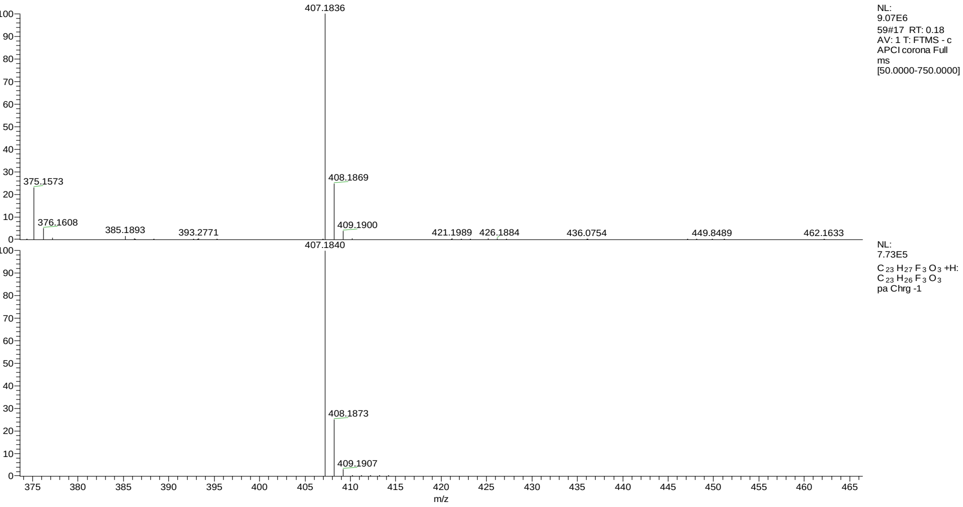

Figure S48. ESI-MS spectrum of intermediate **6k**.

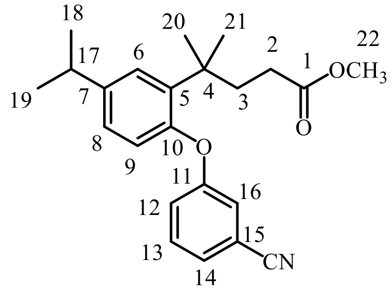

Intermediate **6l**, yellowish oily liquid; yield 98.2%; FT-IR (KBr, cm^{-1}): 2959, 2927, 2867 (C-H), 2236 (C≡N), 1739 (C=O), 1578, 1481, 1432, 1253, 1205, 1165, 1082 (Ar-C=C); ^{1}H NMR (600 MHz, $CDCl_3$) δ 7.41 (t, *J* = 8.0 Hz, 1H, C_{14}-H), 7.33 (d, *J* = 7.7 Hz, 1H, C_{13}-H), 7.23 (d, *J* = 9.8 Hz, 1H, C_{12}-H), 7.20 – 7.14 (m, 2H, C_6-H, C_{16}-H), 7.06 (dd, *J* = 8.3, 2.0 Hz, 1H, C_8-H), 6.74 (d, *J* = 8.3 Hz, 1H, C_9-H), 3.58 (s, 3H, C_{22}-H), 2.90 (t, *J* = 13.8 Hz, 1H, C_{17}-H), 2.13 – 2.05 (m, 4H, C_2-H, C_3-H), 1.37 (s, 6H, C_{20}-H, C_{21}-H), 1.26 (d, *J* = 6.9 Hz, 6H, C_{18}-H, C_{19}-H); ^{13}C NMR (151 MHz, $CDCl_3$) δ 174.31, 158.26, 151.92, 145.05, 137.99, 130.64, 127.06, 125.97, 125.39, 122.80, 120.83, 120.64, 118.40, 113.46, 51.51, 37.72, 36.38, 33.83, 30.51, 28.44, 24.15; ESI-MS m/z: 366.2055 [M + H^+].

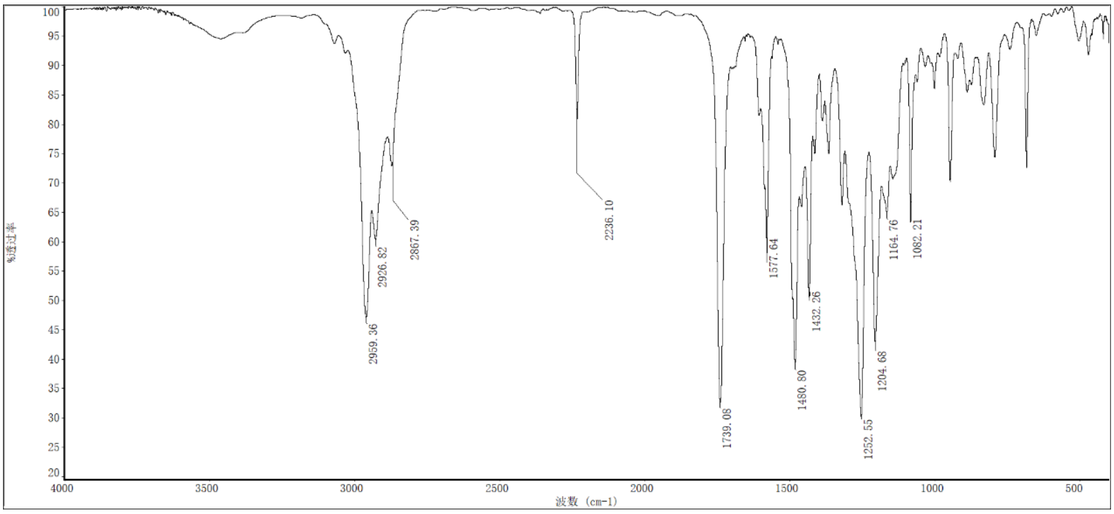

Figure S49. FT-IR spectrum of intermediate **6l**.

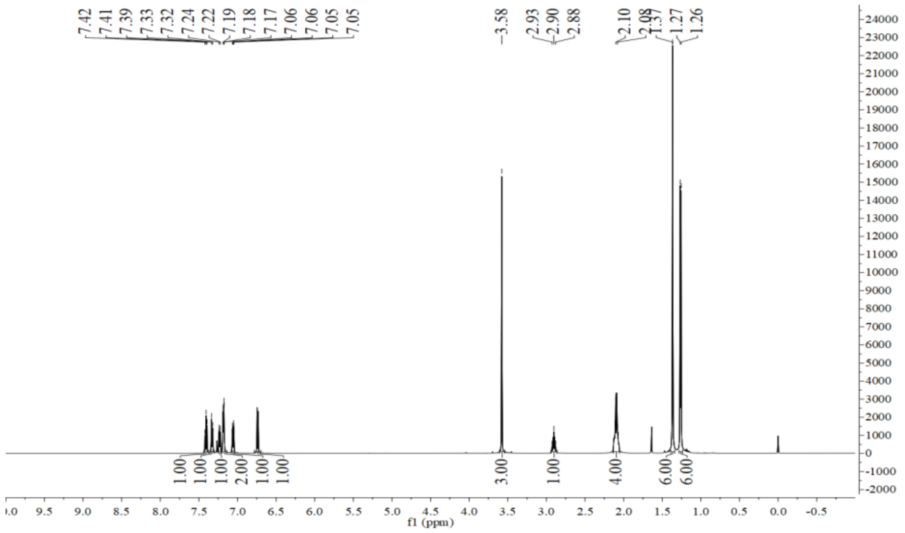

Figure S50. ^{1}H-NMR spectrum of intermediate **6l**.

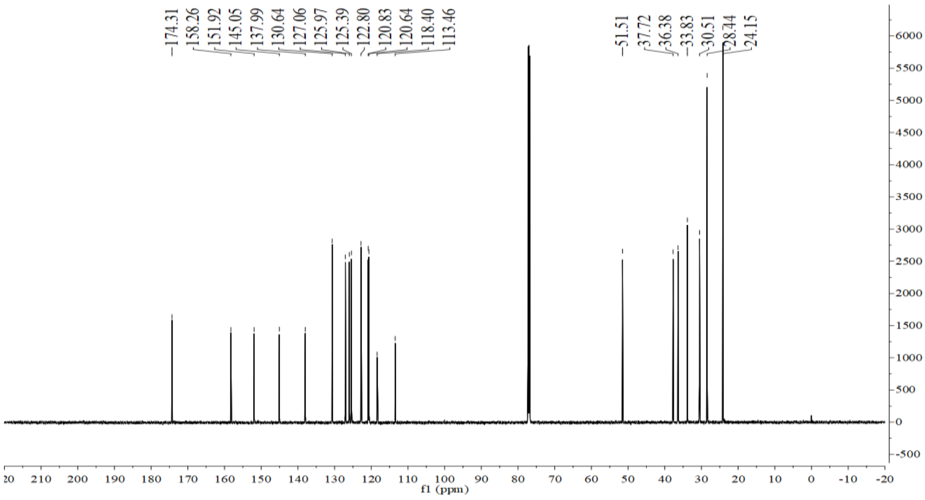

Figure S51. ^{13}C-NMR spectrum of intermediate **6l**.

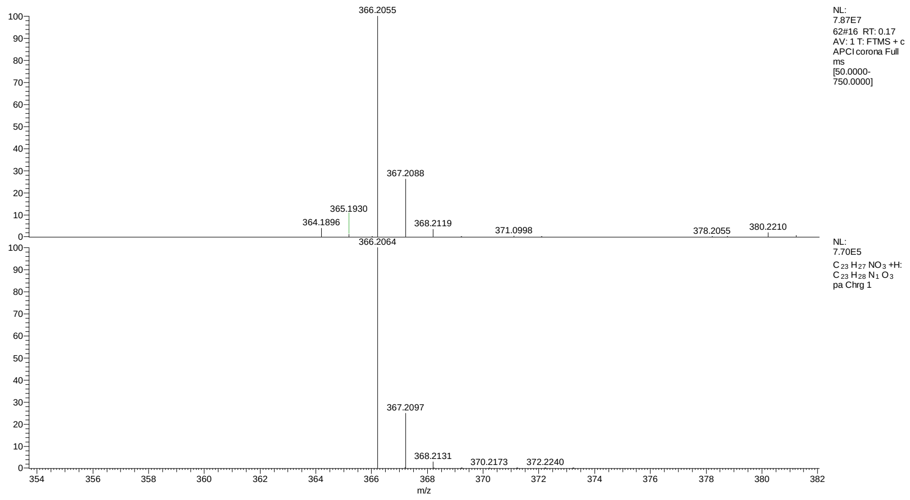

Figure S52. ESI-MS spectrum of intermediate **6l**.

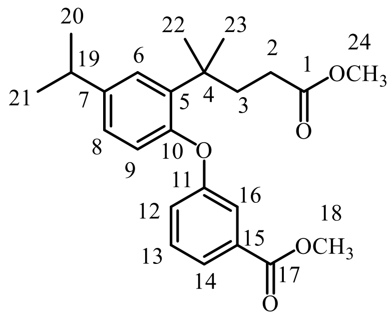

Intermediate **6m**, colorless oily liquid; yield 90.6%; FT-IR (KBr, cm^{-1}): 2957, 2926, 2874 (C-H), 1728 (C=O), 1582, 1484, 1444, 1274, 1226, 1197, 1162, 1098, 1082 (Ar-C=C); ^{1}H NMR (500 MHz, $CDCl_3$) δ 7.74 (d, J = 7.7 Hz, 1H, C_{14}-H), 7.64 (s, 1H), 7.37 (t, J = 8.0 Hz, 1H, C_{16}-H), 7.16 (d, J = 8.9 Hz, 2H, C_{13}-H, C_6-H), 7.01 (d, J = 10.4 Hz, 1H, C_8-H), 6.72 (d, J = 8.3 Hz, 1H, C_9-H), 3.89 (s, 3H, C_{18}-H), 3.57 (s, 3H, C_{24}-H), 2.89 (dt, J = 13.8, 6.9 Hz, 1H, C_{19}-H), 2.17 (dd, J = 14.4, 4.9 Hz, 2H, C_2-H), 2.11 (dd, J = 14.4, 4.9 Hz, 2H, C_3-H), 1.39 (s, 6H, C_{22}-H, C_{23}-H), 1.25 (d, J = 6.9 Hz, 6H, C_{20}-H, C_{21}-H); ^{13}C NMR (126 MHz, $CDCl_3$) δ 174.45, 166.60, 157.64, 152.93, 143.99, 137.46, 131.82, 129.63, 126.73, 125.09, 123.70, 122.88, 119.87, 119.31, 52.17, 51.39, 37.70, 36.24, 33.75, 30.58, 28.38, 24.16; ESI-MS m/z: 399.2158 [M + H^+].

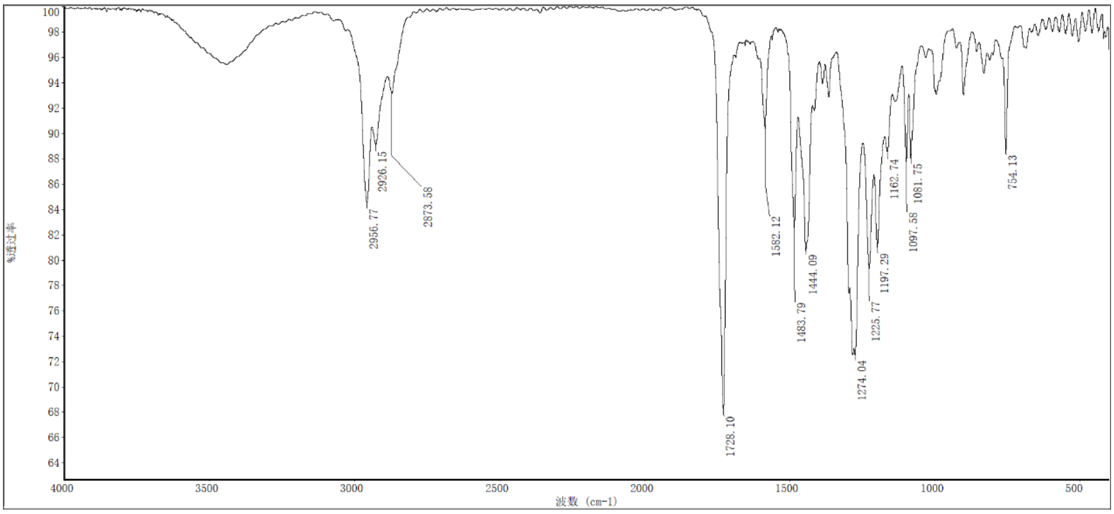

Figure S53. FT-IR spectrum of intermediate **6m**.

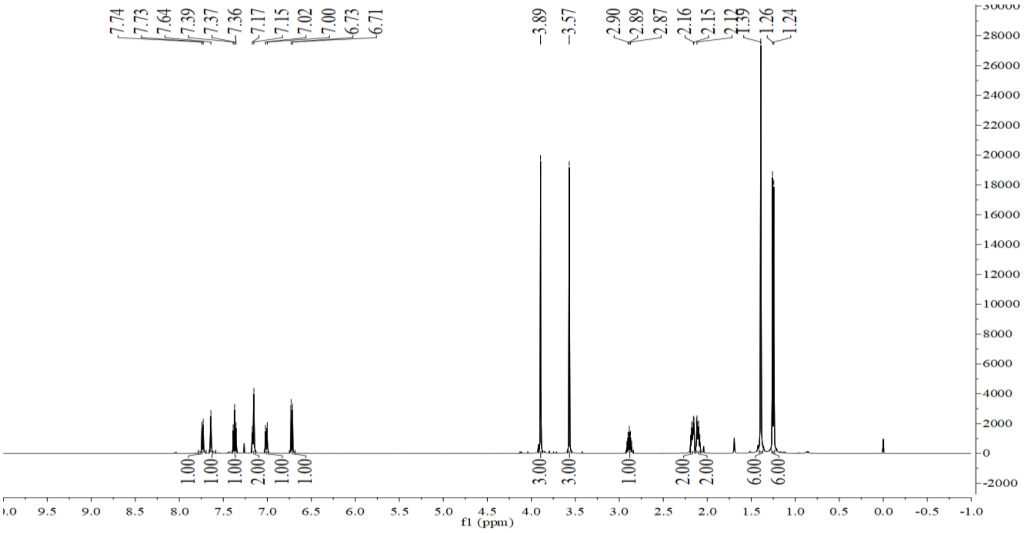

Figure S54. ^{1}H-NMR spectrum of intermediate **6m**.

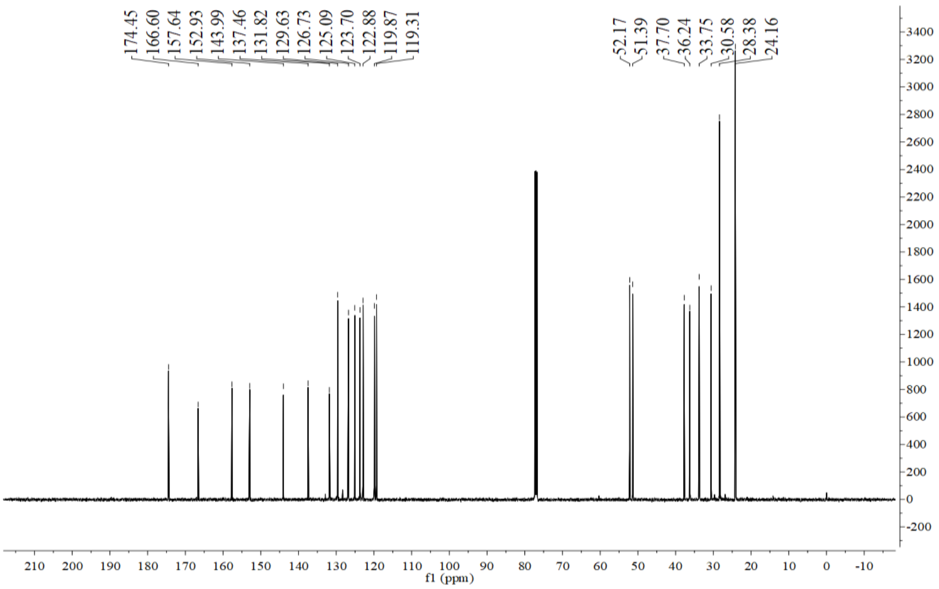

Figure S55. ^{13}C-NMR spectrum of intermediate **6m**.

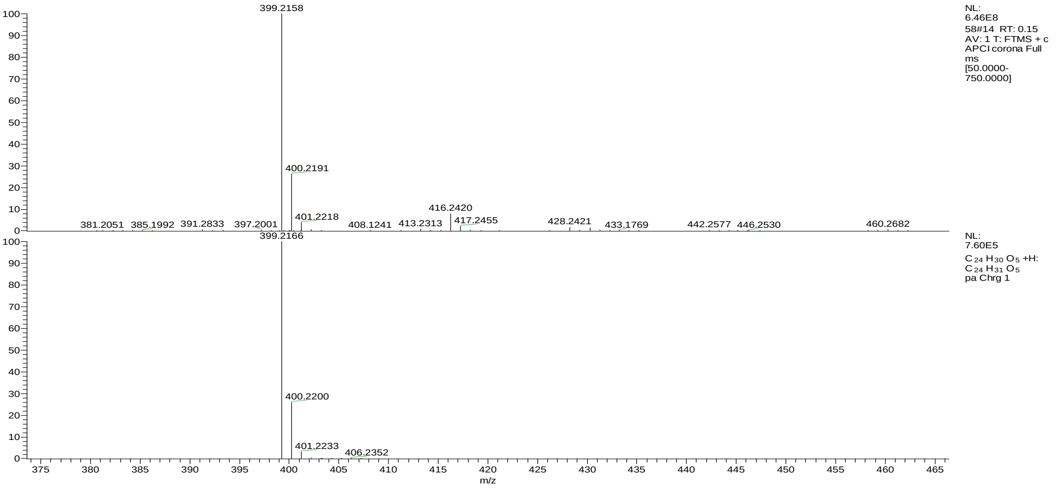

Figure S56. ESI-MS spectrum of intermediate **6m**.

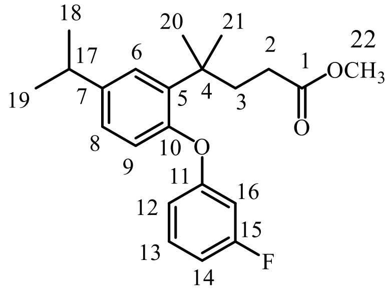

Intermediate **6n**, colorless oily liquid; yield 91.8%; FT-IR (KBr, cm^{-1}): 2959, 2931, 2872 (C-H), 1741 (C=O), 1598, 1483, 1448, 1269, 1208, 1121 (Ar-C=C); ^{1}H NMR (600 MHz, $CDCl_3$) δ 7.26 – 7.21 (m, 1H, C_{13}-H), 7.15 (d, *J* = 2.2 Hz, 1H, C_6-H), 7.03 (dd, *J* = 8.3, 2.2 Hz, 1H, C_8-H), 6.78 (d, *J* = 8.3 Hz, 1H, C_9-H), 6.74 (ddd, *J* = 10.0, 8.2, 2.3 Hz, 2H, C_{14}-H, C_{16}-H), 6.66 (dt, *J* = 10.5, 2.3 Hz, 1H, C_{12}-H), 3.57 (s, 3H, C_{22}-H), 2.89 (dt, *J* = 13.8, 6.9 Hz, 1H, C_{17}-H), 2.16 – 2.12 (m, 2H, C_2-H), 2.11 – 2.07 (m, 2H, C_3-H), 1.38 (s, 6H, C_{20}-H, C_{21}-H), 1.25 (d, *J* = 6.9 Hz, 6H, C_{18}-H, C_{19}-H); ^{13}C NMR (151 MHz, $CDCl_3$) δ 174.45, 164.39, 162.76, 159.14 (d, *J* = 10.6 Hz), 152.56, 144.35, 137.71, 130.37 (d, *J* = 9.9 Hz), 126.76, 125.10, 120.60, 113.78 (d, *J* = 3.0 Hz), 109.29, 109.15, 105.74, 105.57, 51.42, 37.72, 36.32, 33.80, 30.59, 28.43, 24.18; ESI-MS m/z: 359.2007 [M + H^{+}].

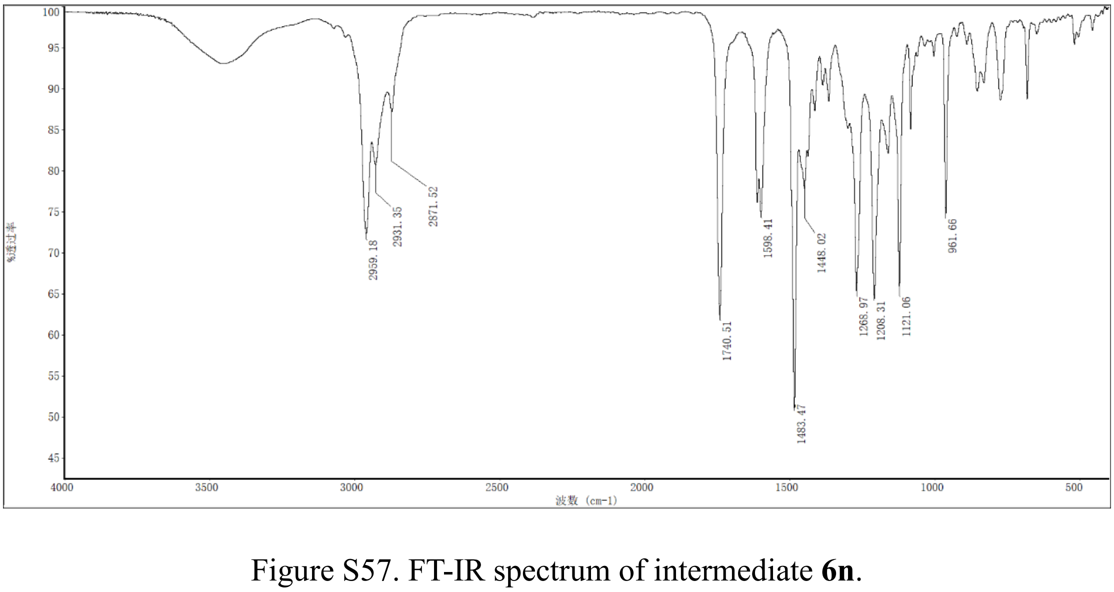

Figure S57. FT-IR spectrum of intermediate **6n**.

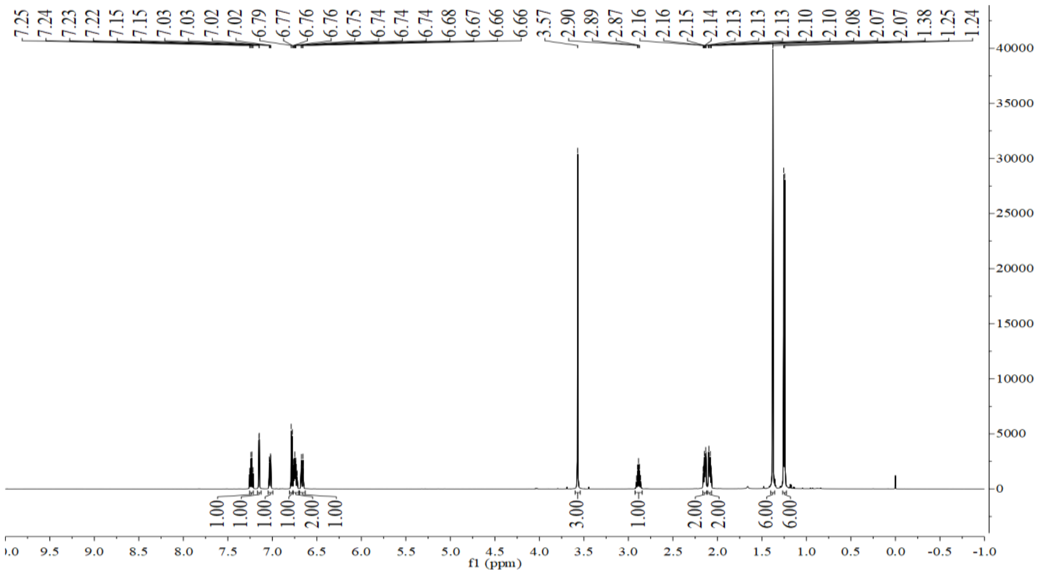

Figure S58. ^{1}H-NMR spectrum of intermediate **6n**.

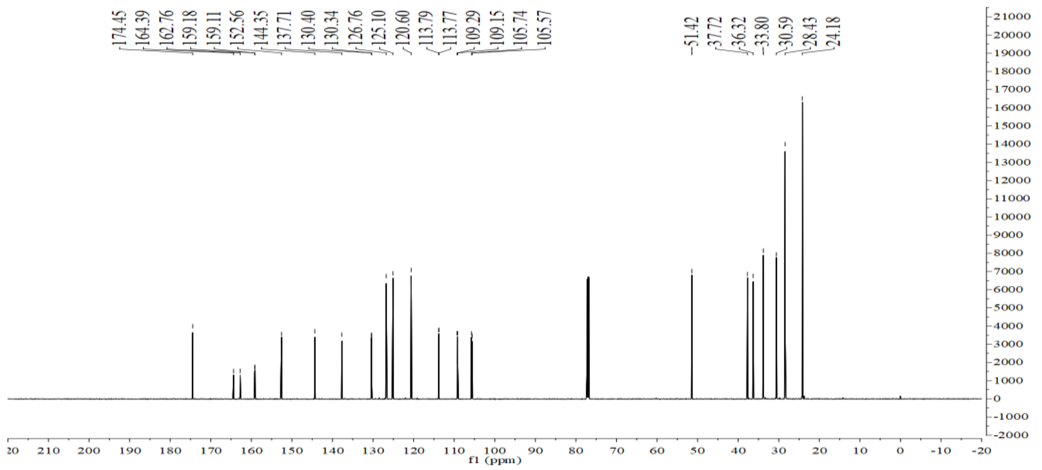

Figure S59. ^{13}C-NMR spectrum of intermediate **6n**.

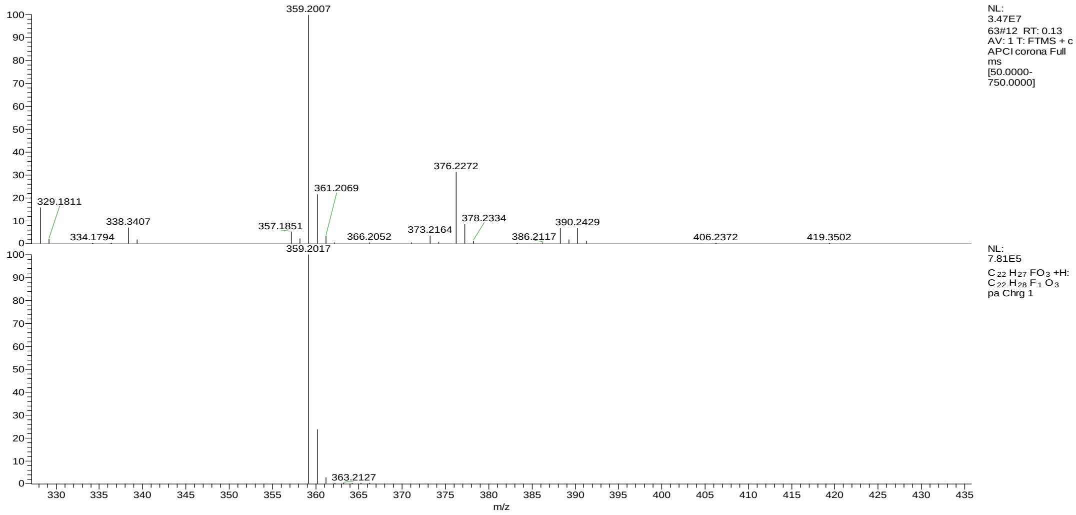

Figure S60. ESI-MS spectrum of intermediate **6n**.

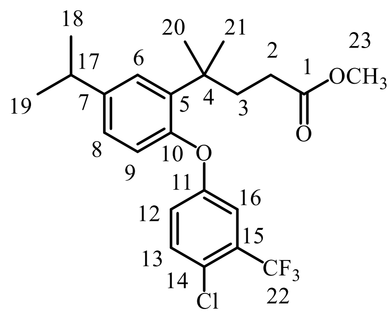

Intermediate **6o**, colorless oily liquid; yield 82.7%; FT-IR (KBr, cm^{-1}): 2960, 2925, 2876 (C-H), 1740 (C=O), 1477, 1424, 1315, 1256, 1230, 1172, 1144, 1082 (Ar-C=C); ^{1}H NMR (600 MHz, $CDCl_3$) δ 7.40 (d, J = 8.8 Hz, 1H, C_{13}-H), 7.31 (d, J = 2.9 Hz, 1H, C_{16}-H), 7.17 (s, 1H, C_6-H), 7.04 (d, J = 5.7 Hz, 2H, C_8-H, C_9-H), 6.72 (d, J = 8.3 Hz, 1H, C_{12}-H), 3.57 (s, 3H, C_{23}-H), 2.89 (dt, J = 13.8, 6.9 Hz, 1H, C_{17}-H), 2.12 (d, J = 8.6 Hz, 2H, C_2-H), 2.09 – 2.05 (m, 2H, C_3-H), 1.37 (s, 6H, C_{20}-H, C_{21}-H), 1.25 (d, J = 6.9 Hz, 6H, C_{18}-H, C_{19}-H); ^{13}C NMR (151 MHz, $CDCl_3$) δ 174.33, 156.36, 152.25, 144.89, 137.80, 132.61, 127.04, 125.37, 122.04, 120.20, 117.48 (d, J = 5.4 Hz), 51.48, 37.73, 36.36, 33.81, 30.55, 28.43, 24.14; ESI-MS m/z: 441.1446 [M + H^+].

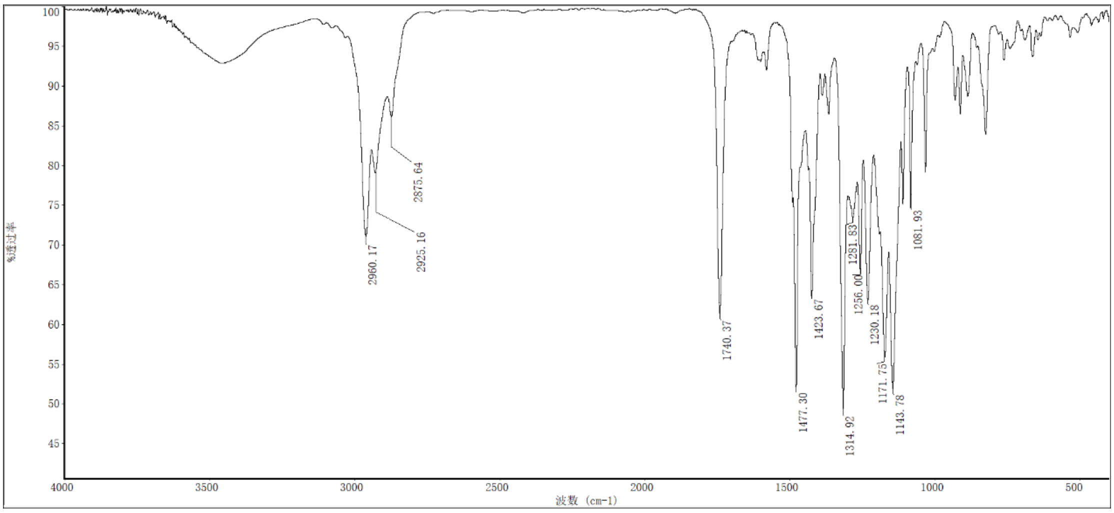

Figure S61. FT-IR spectrum of intermediate **6o**.

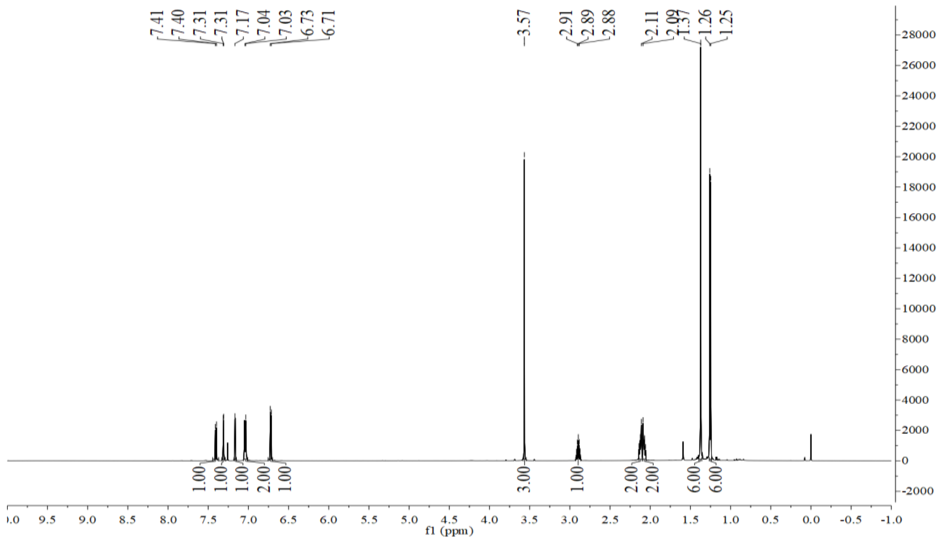

Figure S62. ^{1}H-NMR spectrum of intermediate **6o**.

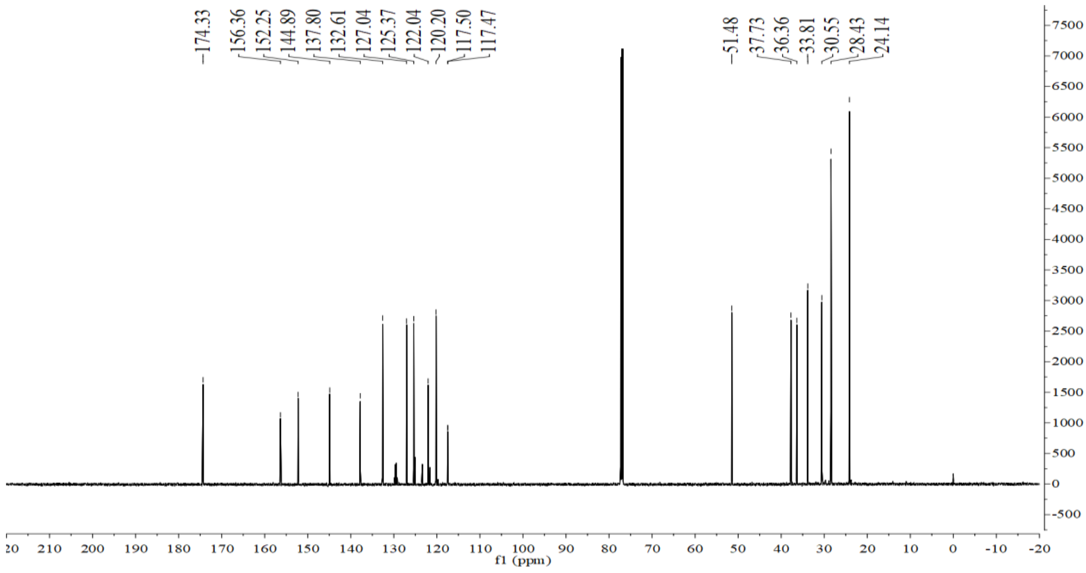

Figure S63. ^{13}C-NMR spectrum of intermediate **6o**.

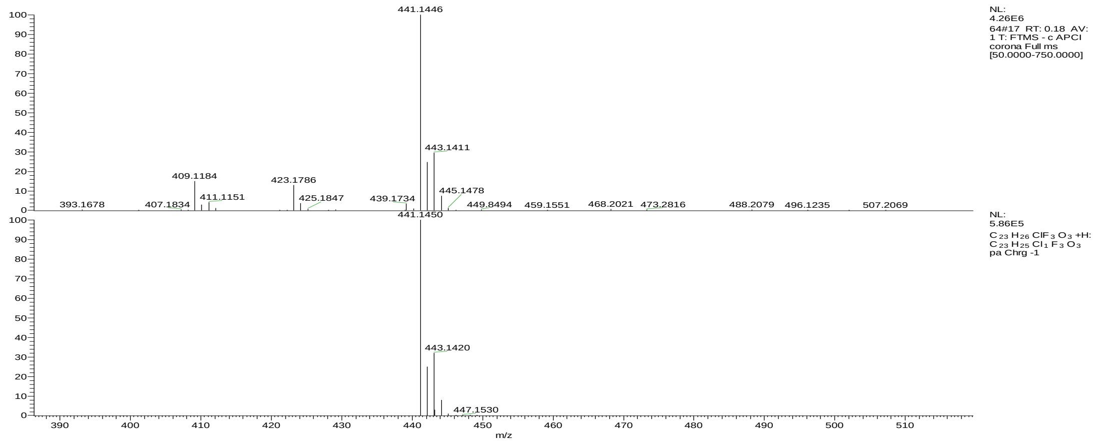

Figure S64. ESI-MS spectrum of intermediate **6o**.

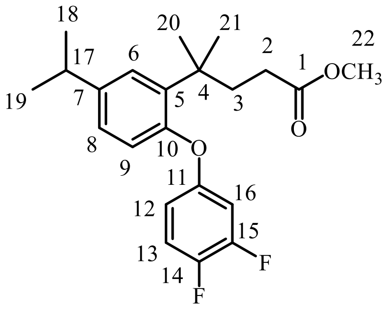

Intermediate **6p**, yellowish oily liquid; yield 89.5%; FT-IR (KBr, cm^{-1}): 2959, 2927, 2872 (C-H), 1740 (C=O), 1617 1514, 1489, 1459, 1437, 1301, 1250, 1204, 1146, 1082 (Ar-C=C); ^{1}H NMR (600 MHz, $CDCl_3$) δ 7.14 (d, J = 2.2 Hz, 1H, C_{13}-H), 7.09 (dd, J = 18.8, 9.1 Hz, 1H, C_6-H), 7.02 (dd, J = 8.3, 2.1 Hz, 1H, C_8-H), 6.79 (dd, J = 13.4, 4.8 Hz, 1H, C_9-H), 6.71 (dd, J = 14.8, 8.7 Hz, 2H, C_{12}-H, C_{16}-H), 3.58 (s, 3H, C_{22}-H), 2.88 (dt, J = 13.8, 6.9 Hz, 1H, C_{17}-H), 2.16 – 2.12 (m, 2H, C_2-H), 2.10 – 2.06 (m, 2H, C_3-H), 1.38 (s, 6H, C_{20}-H, C_{21}-H), 1.24 (d, J = 6.9 Hz, 6H, C_{18}-H, C_{19}-H); ^{13}C NMR (151 MHz, $CDCl_3$) δ 174.42, 152.95, 144.30, 137.46, 126.83, 125.16, 119.94, 117.59 – 117.30 (m), 113.86 (d, J = 2.3 Hz), 107.87, 107.73, 51.45, 37.73, 36.29, 33.78, 30.59, 28.39, 24.17; ESI-MS m/z: 377.1914 [M + H^+].

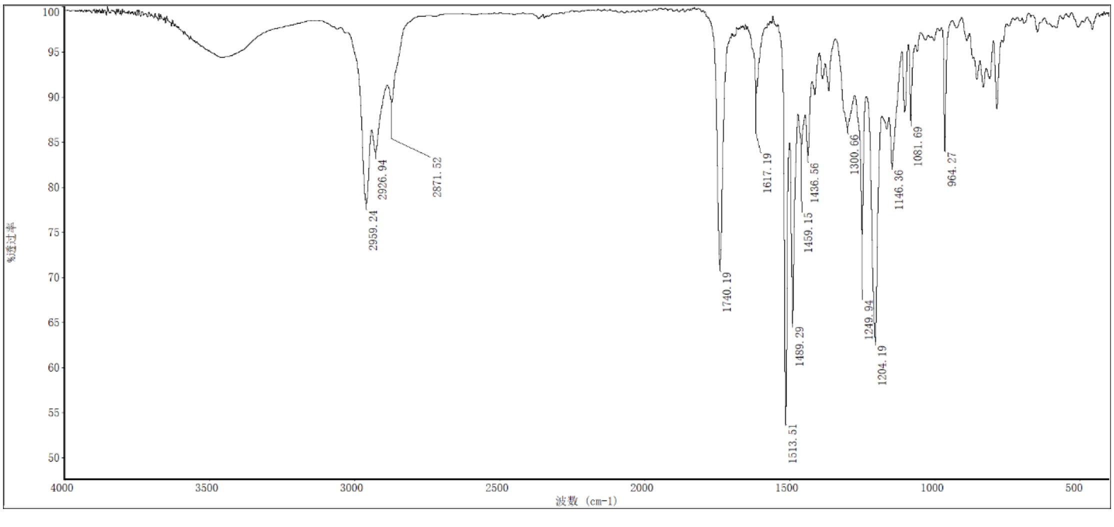

Figure S65. FT-IR spectrum of intermediate **6p**.

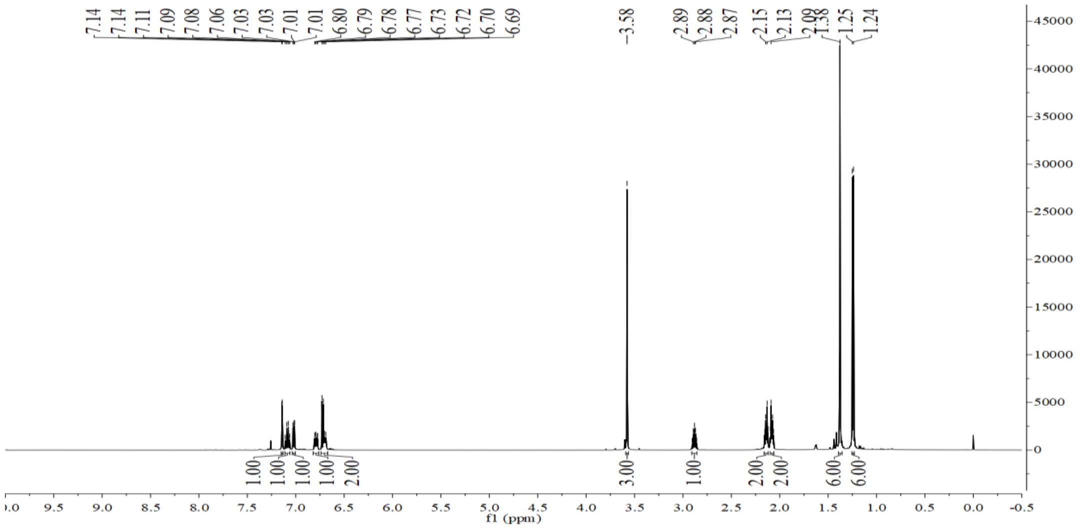

Figure S66. ^{1}H-NMR spectrum of intermediate **6p**.

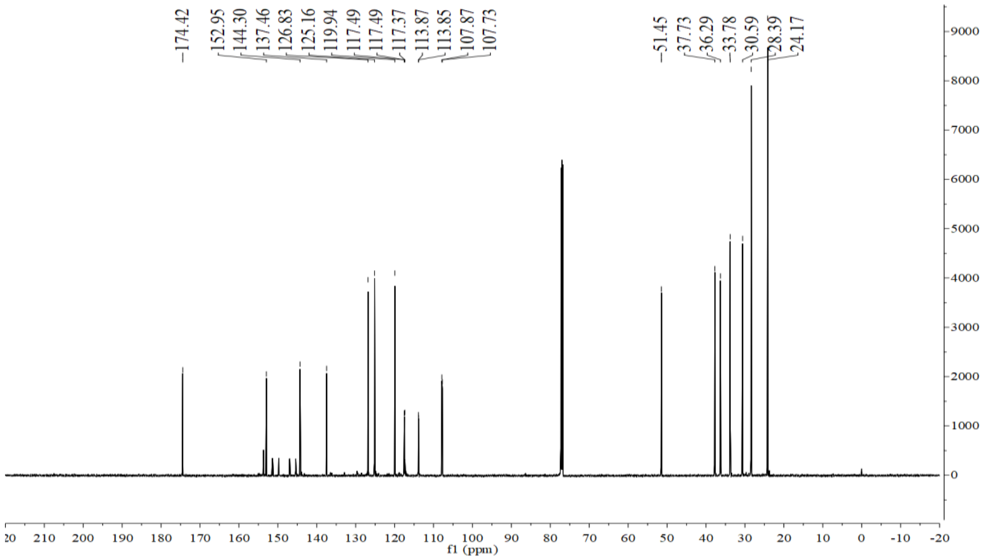

Figure S67. ^{13}C-NMR spectrum of intermediate **6p**.

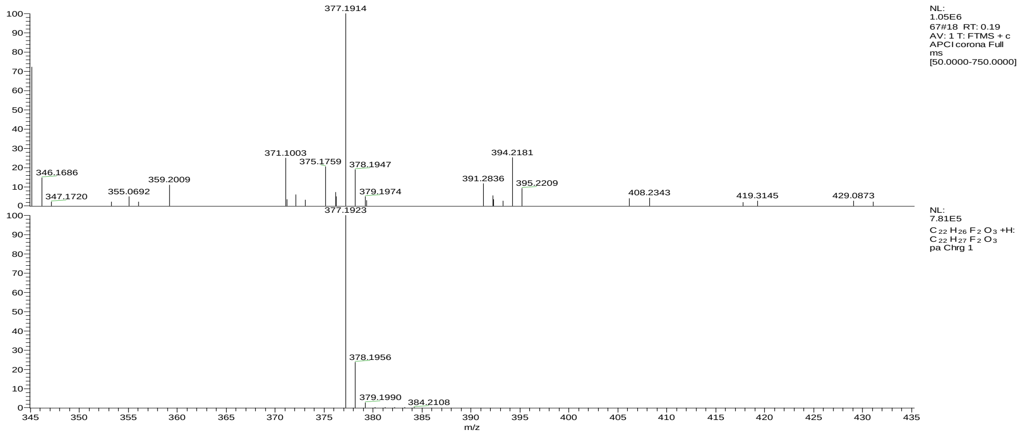

Figure S68. ESI-MS spectrum of intermediate **6p**.

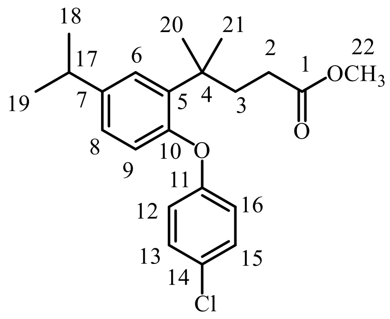

Intermediate **6q**, colorless oily liquid; yield 95.2%; FT-IR (KBr, cm^{-1}): 2960, 2923, 2872 (C-H), 1740 s(C=O), 1588, 1483, 1436, 1366, 1234, 1198, 1163, 1083 (Ar-C=C); ^1H NMR (500 MHz, $CDCl_3$) δ 7.28 – 7.21 (m, 2H, C_{13}-H, C_{15}-H), 7.14 (d, J = 2.2 Hz, 1H, C_6-H), 7.00 (dd, J = 8.3, 2.2 Hz, 1H, C_8-H), 6.90 (d, J = 8.9 Hz, 2H, C_{12}-H, C_{16}-H), 6.72 (d, J = 8.3 Hz, 1H, C_9-H), 3.57 (s, 3H, C_{22}-H), 2.92 – 2.82 (m, 1H, C_{17}-H), 2.14 (q, J = 7.6 Hz, 2H, C_2-H), 2.07 (d, J = 12.5 Hz, 2H, C_3-H), 1.38 (s, 6H, C_{20}-H, C_{21}-H), 1.24 (d, J = 6.9 Hz, 6H, C_{18}-H, C_{19}-H); ^{13}C NMR (126 MHz, $CDCl_3$) δ 174.46, 156.26, 153.03, 143.98, 137.43, 129.56, 127.44, 126.69, 125.02, 119.93, 119.57, 51.43, 37.69, 36.22, 33.74, 30.58, 28.37, 24.16; ESI-MS m/z: 375.1712 [M + H^+].

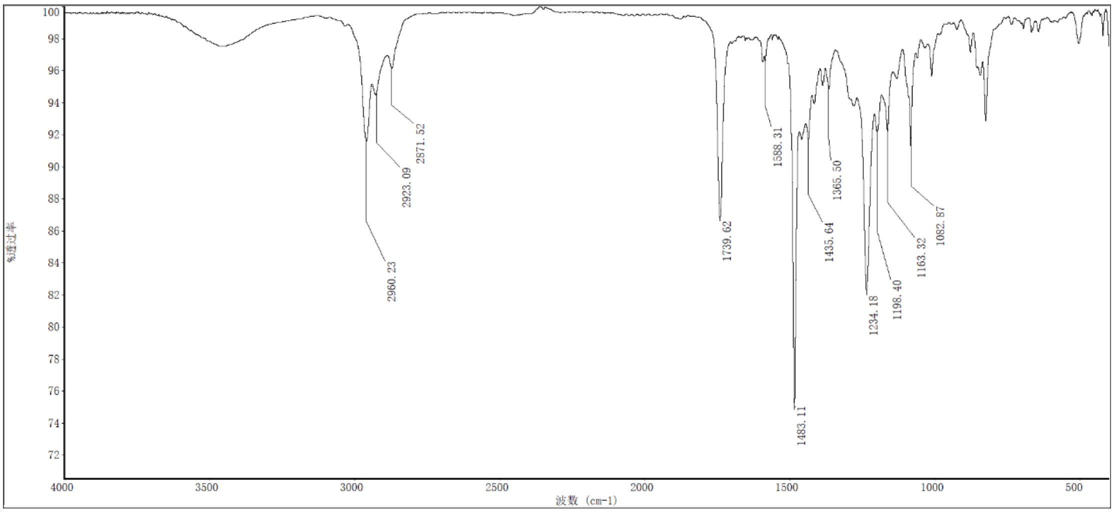

Figure S69. FT-IR spectrum of intermediate **6q**.

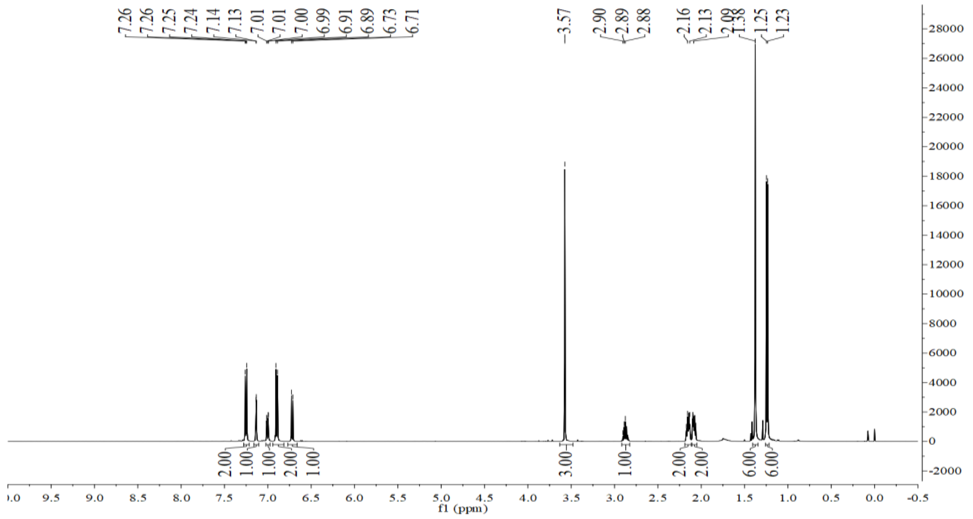

Figure S70. ^{1}H-NMR spectrum of intermediate **6q**.

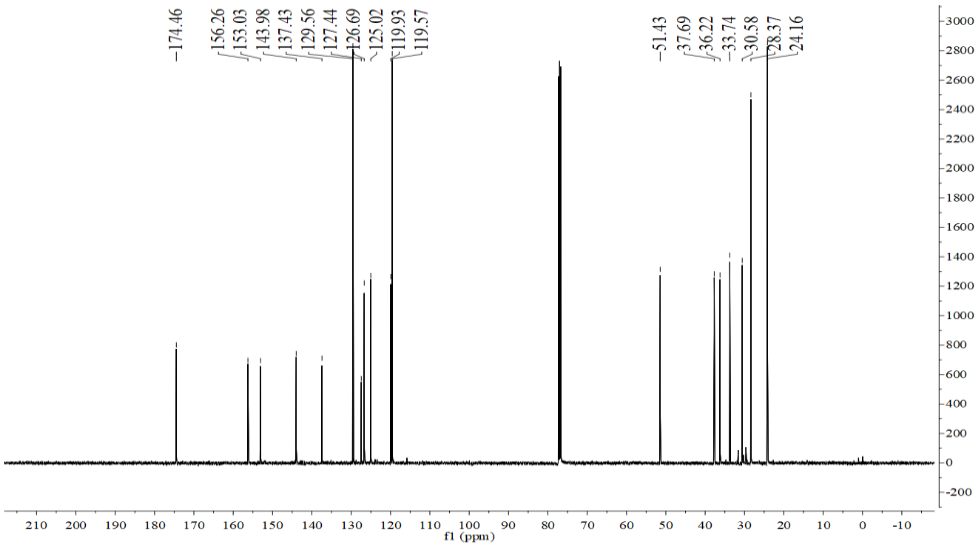

Figure S71. ^{13}C-NMR spectrum of intermediate **6q**.

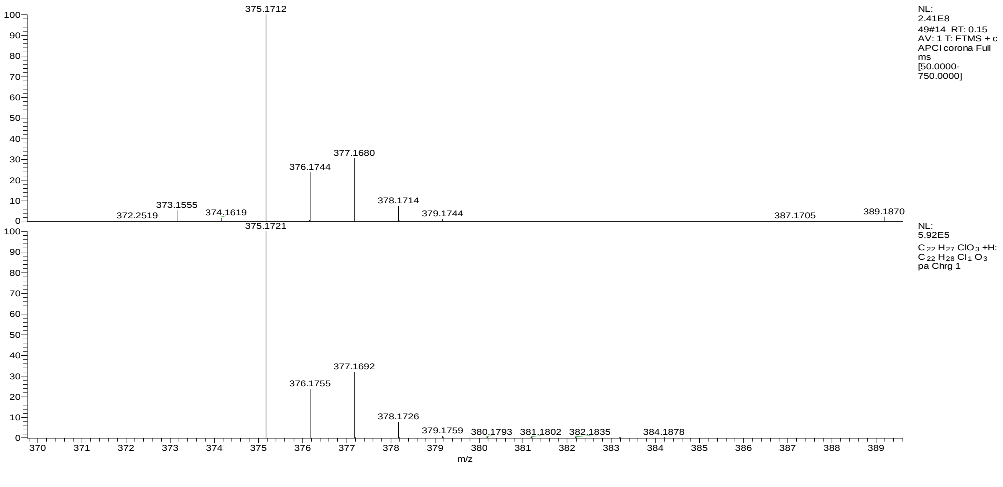

Figure S72. ESI-MS spectrum of intermediate **6q**.

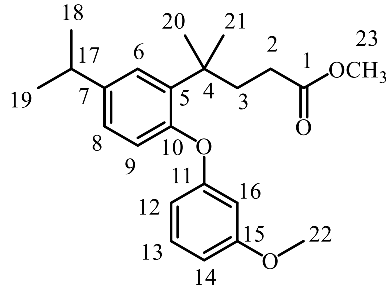

Intermediate **6r**, colorless oily liquid; yield 86.3%; FT-IR (KBr, cm^{-1}): 2958, 2926, 2874 (C-H), 1740 (C=O), 1604, 1486, 1452, 1363, 1282, 1263, 1213, 1141, 1083, 1040 (Ar-C=C); ^{1}H NMR (600 MHz, $CDCl_3$) δ 7.19 (t, J = 8.5 Hz, 1H, C_{13}-H), 7.12 (d, J = 2.2 Hz, 1H, C_6-H), 6.99 (d, J = 6.1 Hz, 1H, C_8-H), 6.77 (d, J = 8.3 Hz, 1H, C_9-H), 6.60 (dd, J = 6.6, 4.7 Hz, 1H, C_{14}-H), 6.57 – 6.47 (m, 2H, C_{12}-H, C_{16}-H), 3.77 (s, 3H, C_{22}-H), 3.57 (s, 3H, C_{23}-H), 2.87 (dt, J = 13.8, 6.9 Hz, 1H, C_{17}-H), 2.20 – 2.15 (m, 2H, C_2-H), 2.09 (dd, J = 9.5, 7.5 Hz, 2H, C_3-H), 1.39 (s, 6H, C_{20}-H, C_{21}-H), 1.24 (d, J = 6.9 Hz, 6H, C_{18}-H, C_{19}-H); ^{13}C NMR (151 MHz, $CDCl_3$) δ 174.58, 160.91, 158.86, 153.17, 143.66, 137.35, 129.99, 126.55, 120.19, 110.68, 108.07, 104.57, 55.32, 51.40, 37.71, 36.24, 33.76, 30.64, 28.42, 24.20; ESI-MS m/z: 371.2209 [M + H^+].

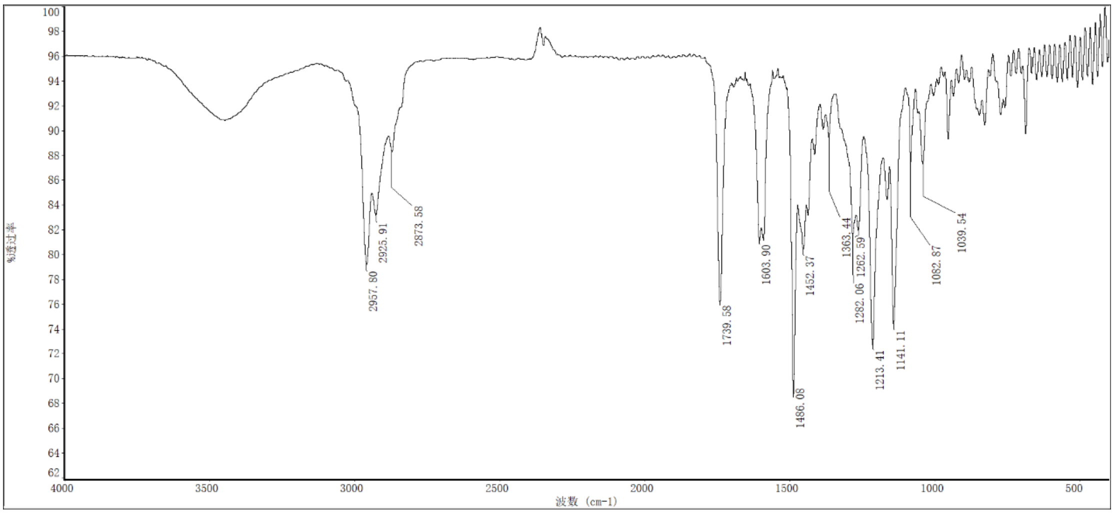

Figure S73. FT-IR spectrum of intermediate **6r**.

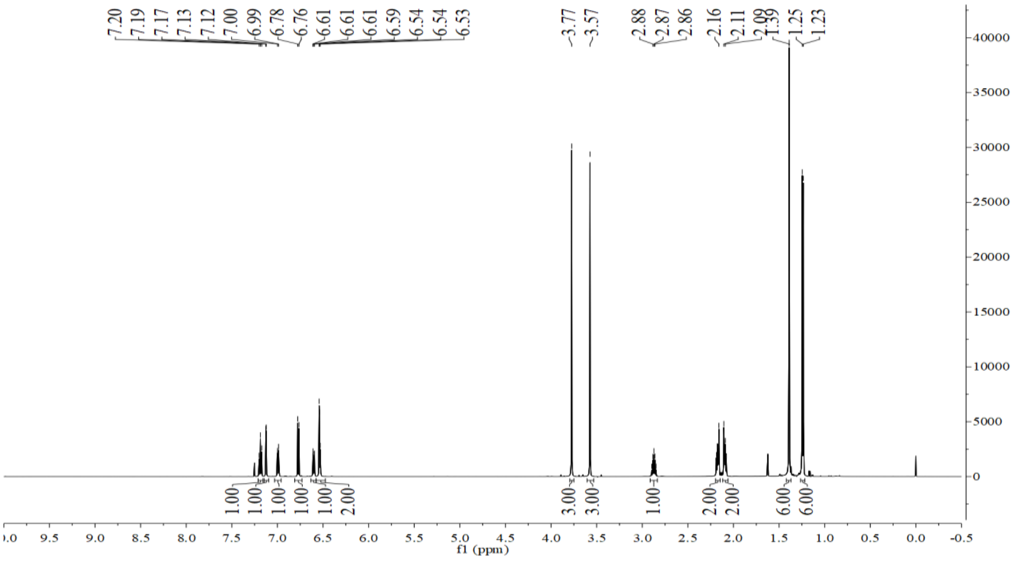

Figure S74. ^{1}H-NMR spectrum of intermediate **6r**.

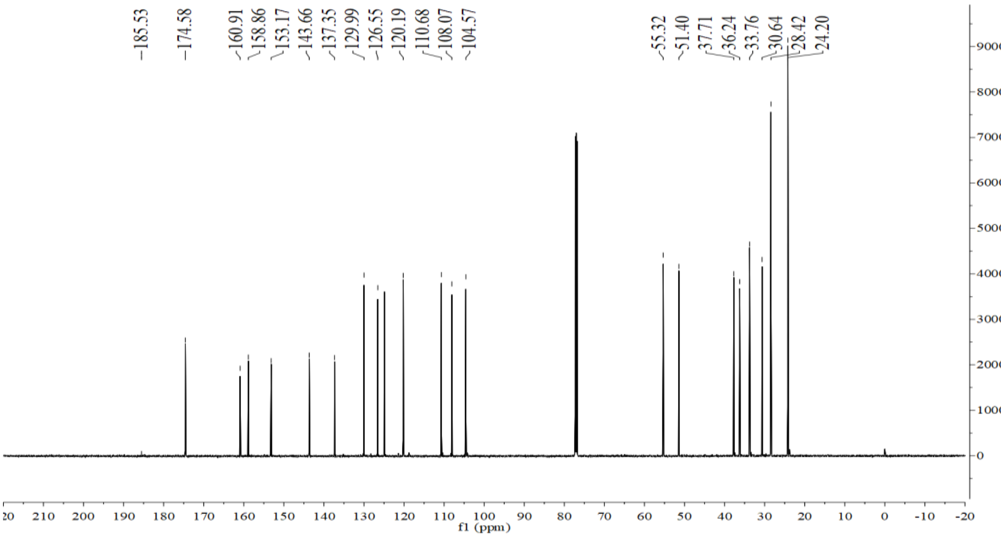

Figure S75. ^{13}C-NMR spectrum of intermediate **6r**.

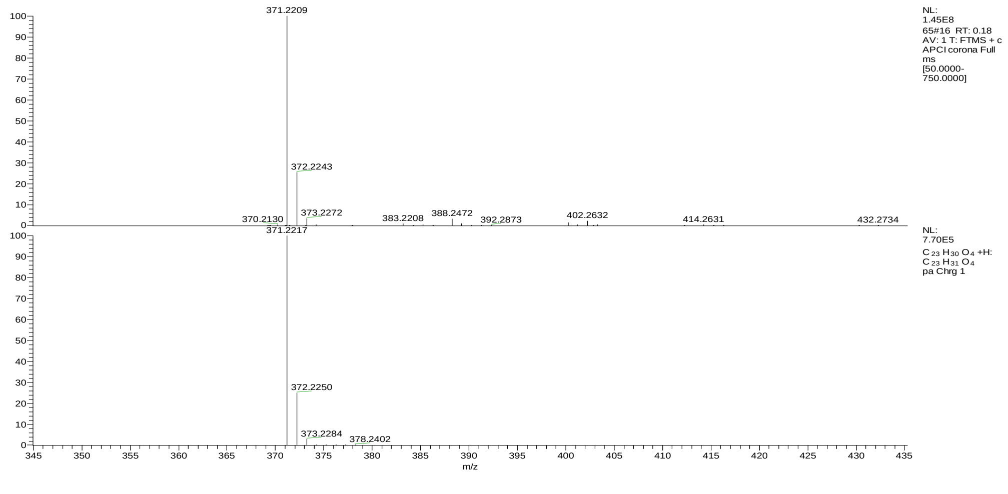

Figure S76. ESI-MS spectrum of intermediate **6r**.

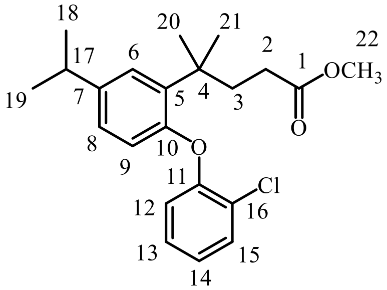

Intermediate **6s**, yellowish oily liquid; yield 87.3%; FT-IR (KBr, cm^{-1}): 2958, 2926, 2872 (C-H), 1740 (C=O), 1582, 1491, 1475, 1446, 1366, 1264, 1239, 1199, 1165, 1082, 1059 (Ar-C=C); ^{1}H NMR (500 MHz, $CDCl_3$) δ 7.45 (dd, J = 8.0, 1.6 Hz, 1H, C_{15}-H), 7.19 – 7.14 (m, 2H, C_6-H, C_{13}-H), 7.04 – 6.96 (m, 2H, C_8-H, C_{14}-H), 6.92 (d, J = 8.2 Hz, 1H, C_{12}-H), 6.60 (d, J = 8.3 Hz, 1H, C_9-H), 3.57 (s, 3H, C_{22}-H), 2.87 (dt, J = 13.8, 6.9 Hz, 1H, C_{17}-H), 2.27 – 2.21 (m, 2H, C_2-H), 2.12 (dd, J = 10.4, 6.4 Hz, 2H, C_3-H), 1.43 (s, 6H, C_{20}-H, C_{21}-H), 1.24 (d, J = 6.9 Hz, 6H, C_{18}-H, C_{19}-H); ^{13}C NMR (126 MHz, $CDCl_3$) δ 174.53, 152.97, 152.60, 143.55, 136.60, 130.64, 127.76, 126.76, 125.54, 124.85, 123.84, 119.70, 118.07, 51.38, 37.80, 36.17, 33.71, 30.69, 28.38, 24.19; ESI-MS m/z: 357.1712 [M + H^+].

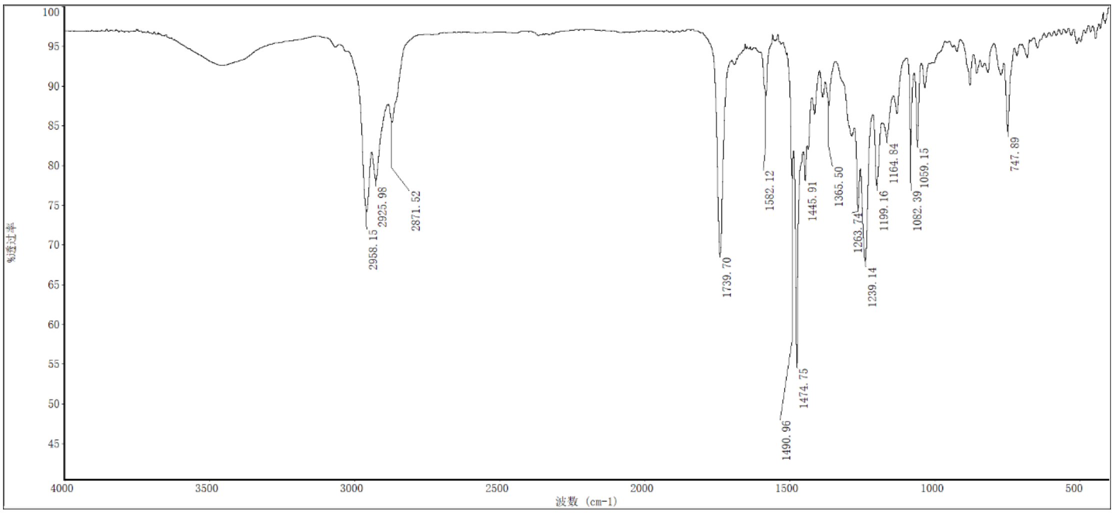

Figure S77. FT-IR spectrum of intermediate **6s**.

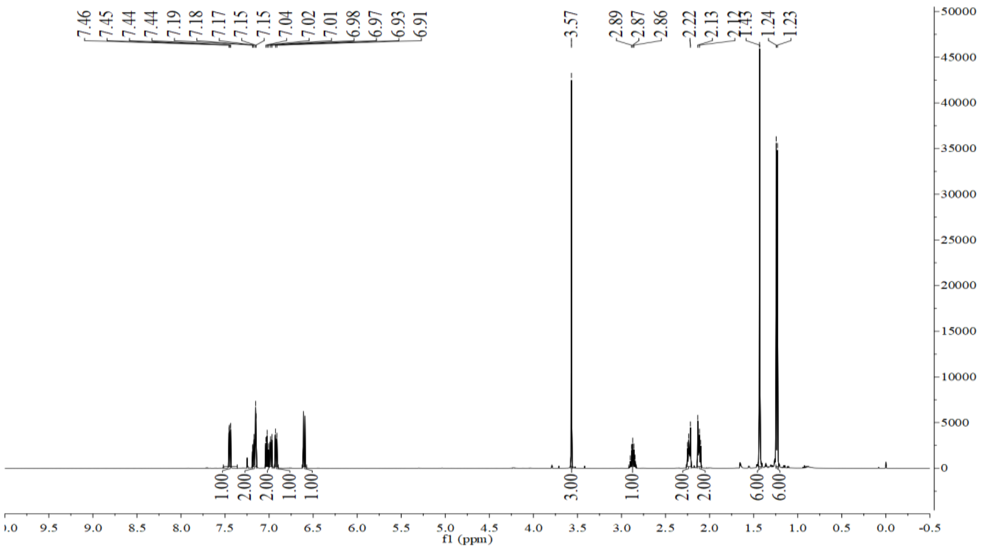

Figure S78. ^{1}H-NMR spectrum of intermediate **6s**.

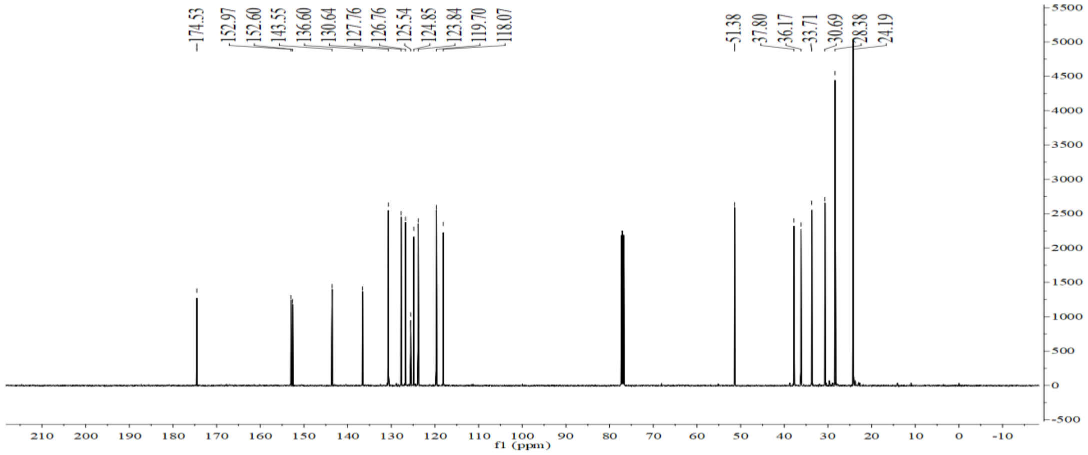

Figure S79. ^{13}C-NMR spectrum of intermediate **6s**.

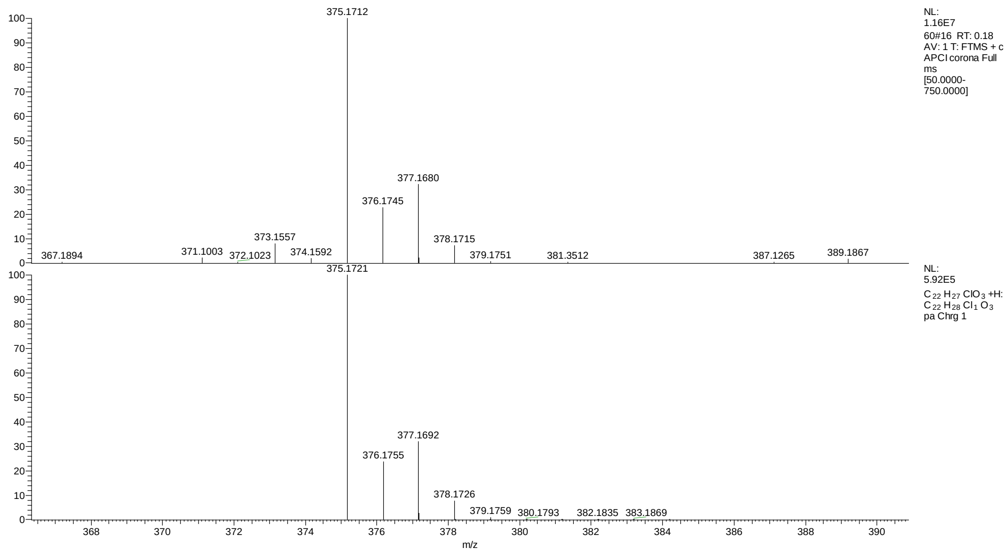

Figure S80. ESI-MS spectrum of intermediate **6s**.

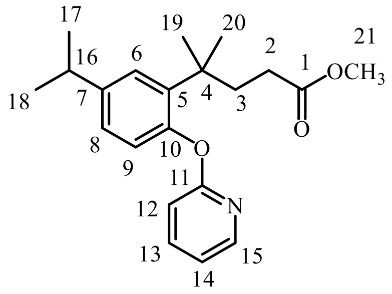

Intermediate **6t**, colorless oily liquid; yield 93.4%; FT-IR (KBr, cm^{-1}): 3447 (Ar–H) 2958, 2925, 2871(C-H), 1736 (C=O), 1593, 1572, 1492, 1466, 1427, 1285, 1264, 1201, 1164, 1082 (Ar-C=C); ^{1}H NMR (500 MHz, $CDCl_3$) δ 8.18 (dd, J = 4.7, 1.6 Hz, 1H, C_{15}-H), 7.68 – 7.64 (m, 1H, C_{13}-H), 7.17 (s, 1H, C_{13}-H), 7.07 (d, J = 8.3 Hz, 1H, C_8-H), 6.97 – 6.94 (m, 1H, C_9-H), 6.89 (dd, J = 8.3, 3.4 Hz, 2H, C_{12}-H, C_{14}-H), 3.54 (s, 3H, C_{21}-H), 2.93 – 2.86 (m, 1H, C_{16}-H), 2.10 (s, 4H, C_2-H, C_3-H), 1.37 (s, 6H, C_{19}-H, C_{20}-H), 1.25 (d, J = 6.9 Hz, 6H, C_{17}-H, C_{18}-H); ^{13}C NMR (126 MHz, $CDCl_3$) δ 174.55, 163.76, 150.58, 147.83, 144.64, 139.26, 137.93, 126.61, 124.97, 122.66, 118.08, 111.75, 51.34, 37.63, 36.51, 33.82, 30.43, 28.57, 24.14; ESI-MS m/z: 342.2068 [M + H^+].

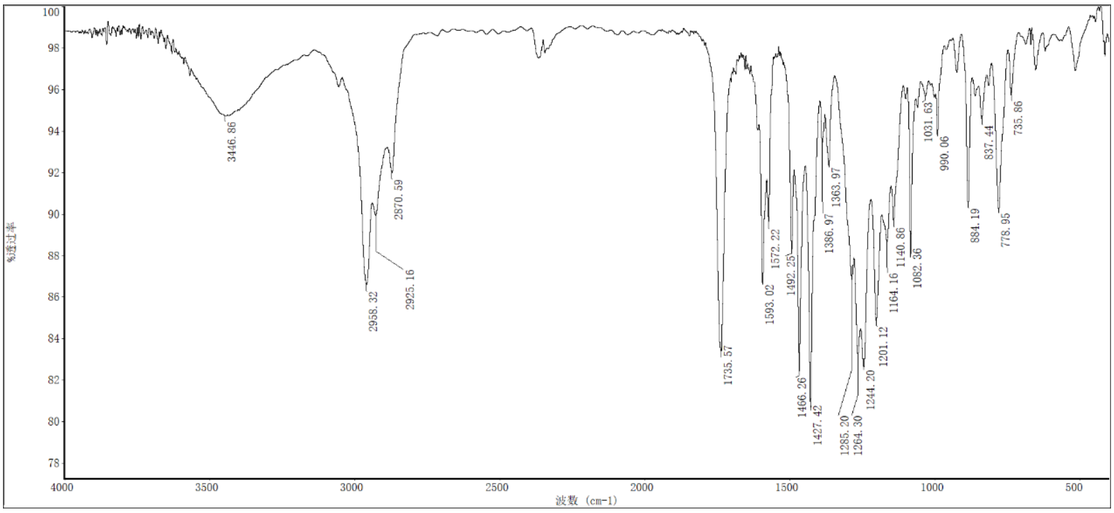

Figure S81. FT-IR spectrum of intermediate **6t**.

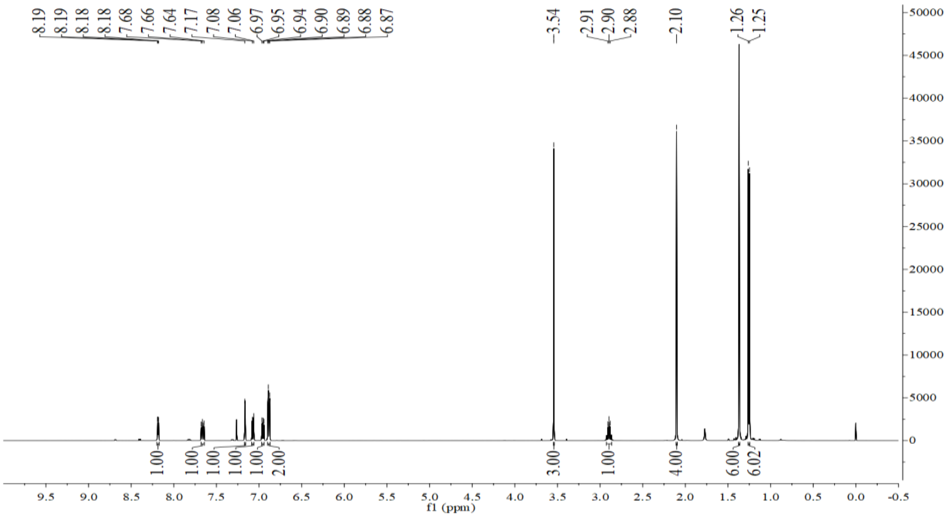

Figure S82. ^{1}H-NMR spectrum of intermediate **6t**.

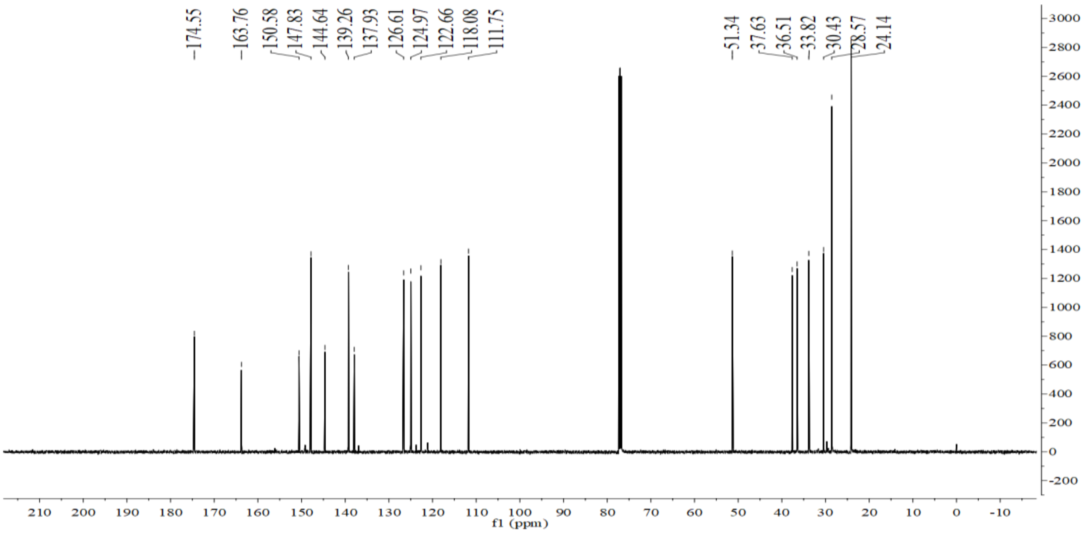

Figure S83. ^{13}C-NMR spectrum of intermediate **6t**.

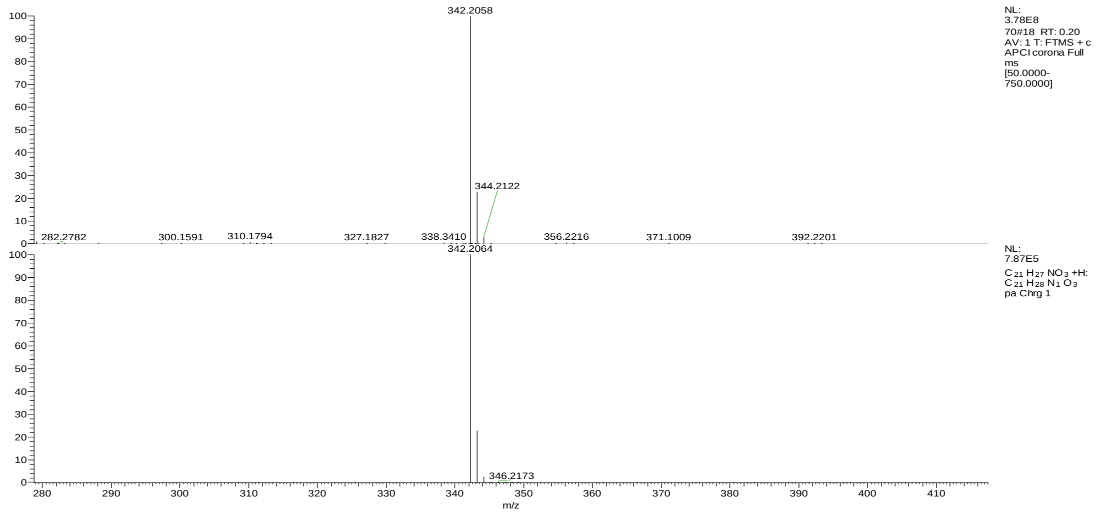

Figure S84. ESI-MS spectrum of intermediate **6t**.

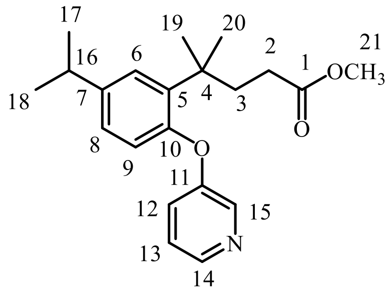

Intermediate **6u**, colorless oily liquid; yield 96.8%; FT-IR (KBr, cm^{-1}): 3447 (Ar–H) 2959, 2929, 2871 (C-H), 1739 (C=O), 1573, 1491, 1475, 1422, 1386, 1242, 1198, 1102, 1082 (Ar-C=C); ^{1}H NMR (500 MHz, $CDCl_3$) δ 8.38 (d, J = 2.3 Hz, 1H, C_{12}-H), 8.32 (dd, J = 4.3, 1.6 Hz, 1H, C_6-H), 7.25 (t, J = 6.9 Hz, 2H, C_{14}-H, C_{15}-H), 7.16 (d, J = 2.1 Hz, 1H, C_{13}-H), 7.02 (dd, J = 8.3, 2.1 Hz, 1H, C_8-H), 6.72 (d, J = 8.3 Hz, 1H, C_9-H), 3.57 (s, 3H, C_{21}-H), 2.94 – 2.84 (m, 1H, C_{16}-H), 2.19 – 2.14 (m, 2H, C_2-H), 2.12 – 2.07 (m, 2H, C_3-H), 1.39 (s, 6H, C_{19}-H, C_{20}-H), 1.25 (d, J = 6.9 Hz, 6H, C_{17}-H, C_{18}-H); ^{13}C NMR (126 MHz, $CDCl_3$) δ 174.40, 154.16, 152.70, 144.45, 143.78, 141.23, 137.58, 126.88, 125.15 (d, J = 16.1 Hz), 124.01, 119.76, 51.48, 37.76, 36.32, 33.78, 30.59, 28.40, 24.1; ESI-MS m/z: 342.2057 [M + H^+].

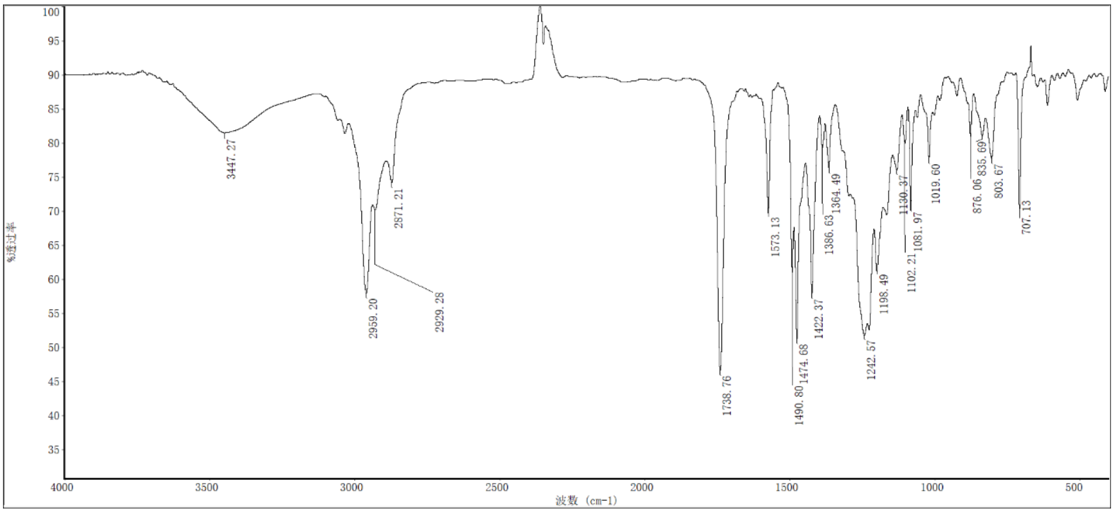

Figure S85. FT-IR spectrum of intermediate **6u**.

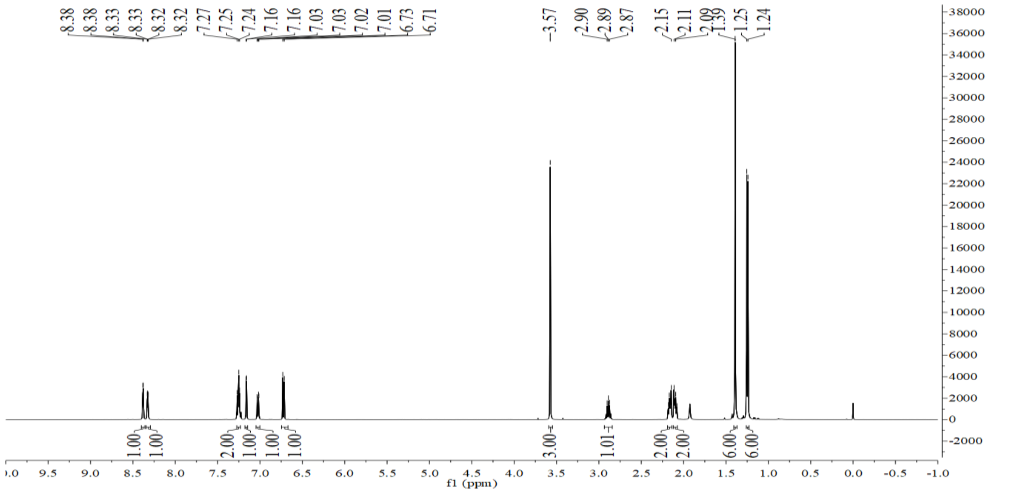

Figure S86. ^{1}H-NMR spectrum of intermediate **6u**.

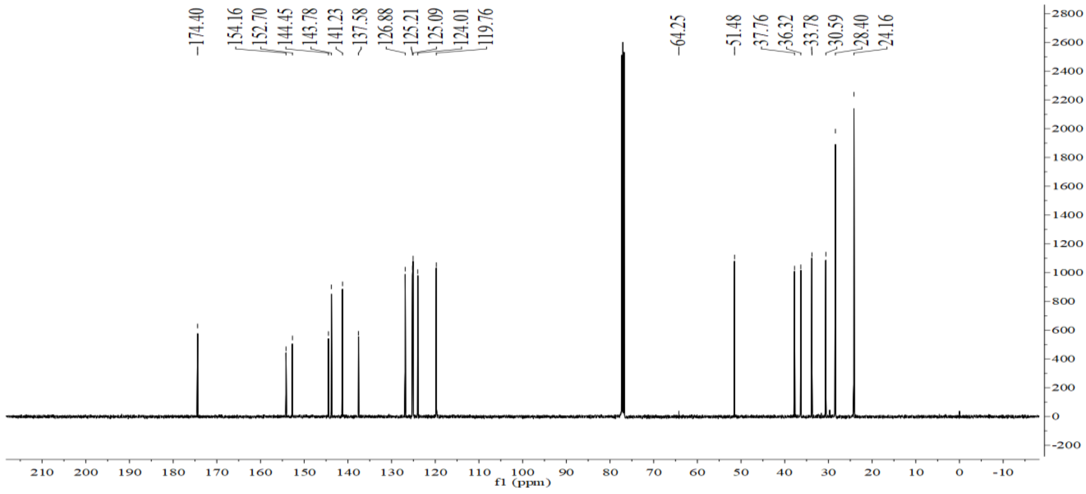

Figure S87. ^{13}C-NMR spectrum of intermediate **6u**.

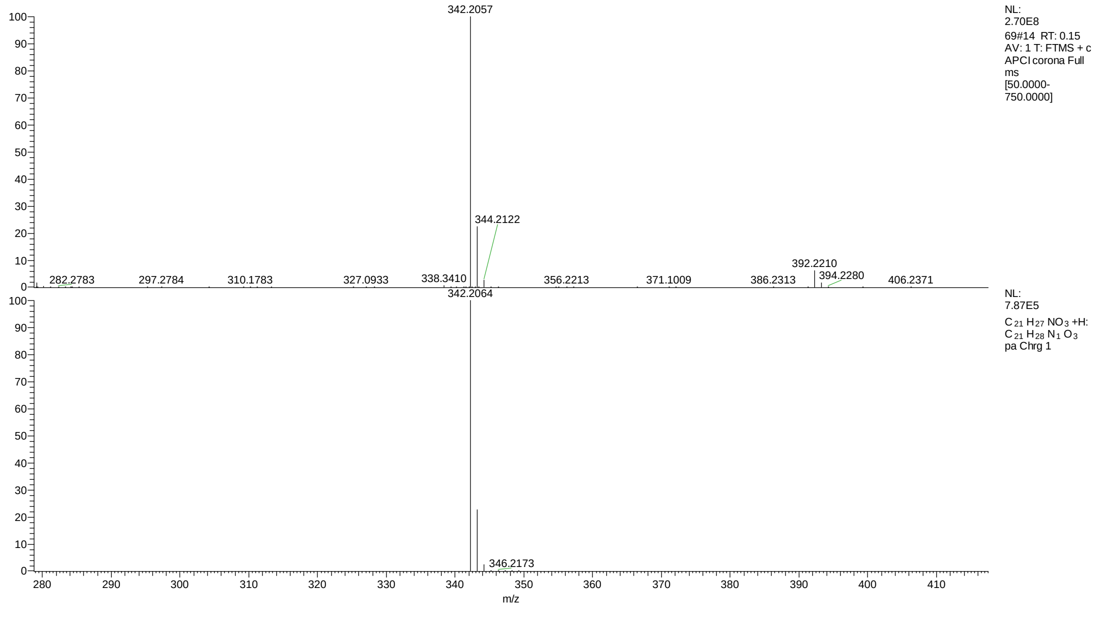

Figure S88. ESI-MS spectrum of intermediate **6u**.

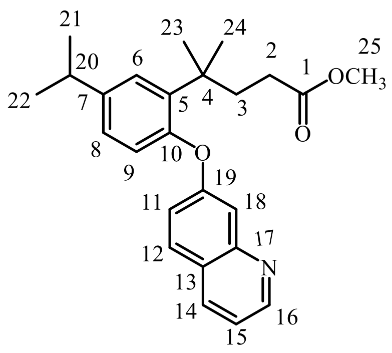

Intermediate **6v**, colorless oily liquid; yield 93.6%; FT-IR (KBr, cm^{-1}): 2959, 2933, 2871(C-H), 1738 (C=O), 1622, 1597, 1489, 1463, 1434, 1322, 1255, 1222, 1203, 1151, 1082 (Ar-C=C); ^{1}H NMR (500 MHz, $CDCl_3$) δ 8.81 (dd, J = 4.2, 1.7 Hz, 1H, C_{16}-H), 8.08 (d, J = 9.2 Hz, 1H, C_{12}-H, C_{14}-H), 8.01 (d, J = 8.4 Hz, 1H, C_{18}-H), 7.47 (dd, J = 9.2, 2.7 Hz, 1H, C_{15}-H), 7.35 (dd, J = 8.3, 4.2 Hz, 1H, C_8-H), 7.22 – 7.19 (m, 2H, C_6-H, C_{11}-H), 7.05 (d, J = 8.3 Hz, 1H, C_9-H), 3.50 (s, 3H, C_{25}-H), 2.95 – 2.88 (m, 1H, C_{20}-H), 2.21 – 2.17 (m, 2H, C_2-H), 2.15 – 2.10 (m, 2H, C_3-H), 1.41 (s, 6H, C_{23}-H, C_{24}-H), 1.27 (d, J = 6.9 Hz, 6H, C_{21}-H, C_{22}-H); ^{13}C NMR (126 MHz, $CDCl_3$) δ 174.42, 155.83, 152.77, 148.75, 144.92, 144.37, 137.79, 135.10, 131.25, 129.19, 126.86, 125.15, 123.15, 121.40, 120.64, 112.32, 51.41, 37.79, 36.35, 33.82, 30.65, 28.47, 24.20; ESI-MS m/z: 392.2213[M + H^+].

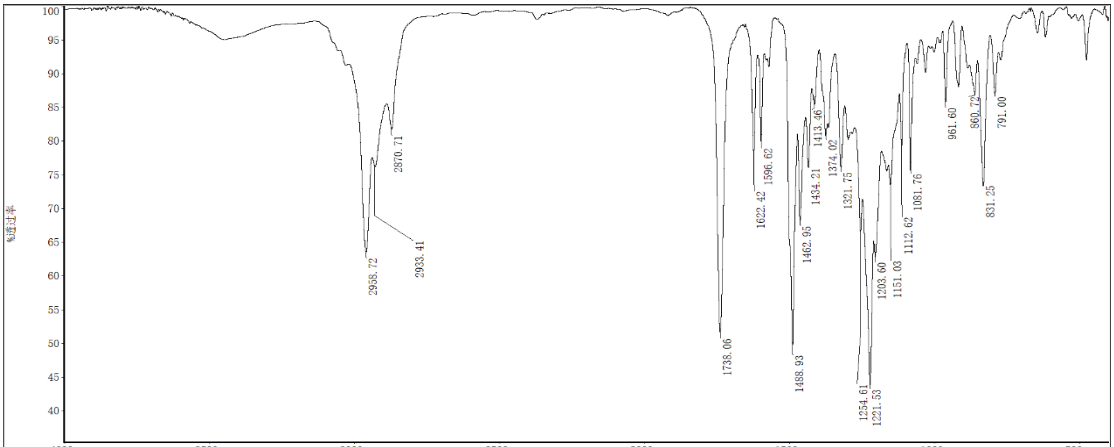

Figure S89. FT-IR spectrum of intermediate **6v**.

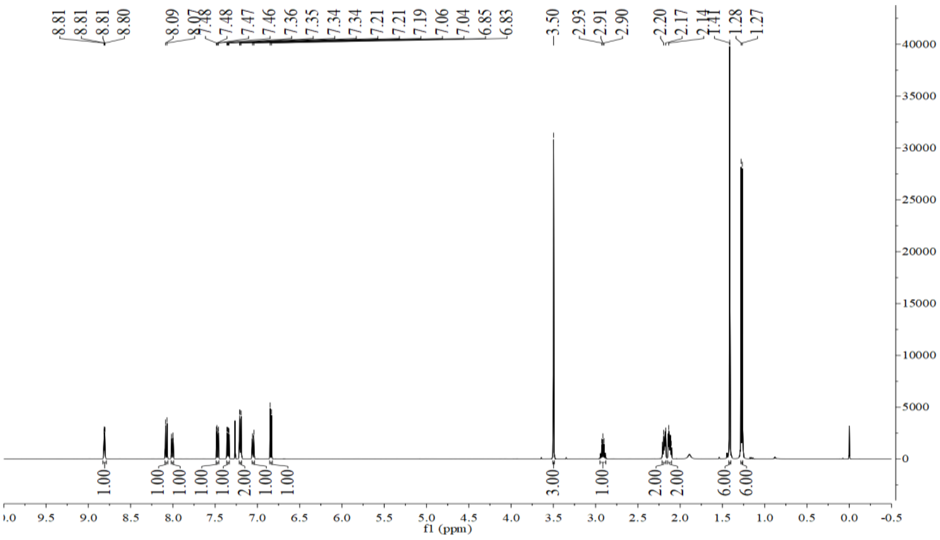

Figure S90. ^{1}H-NMR spectrum of intermediate **6v**.

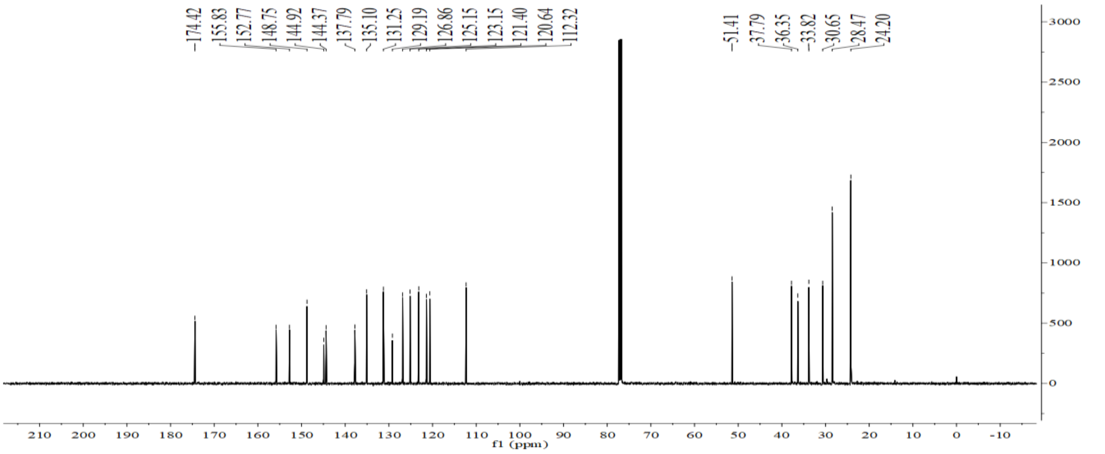

Figure S91. ^{13}C-NMR spectrum of intermediate **6v**.

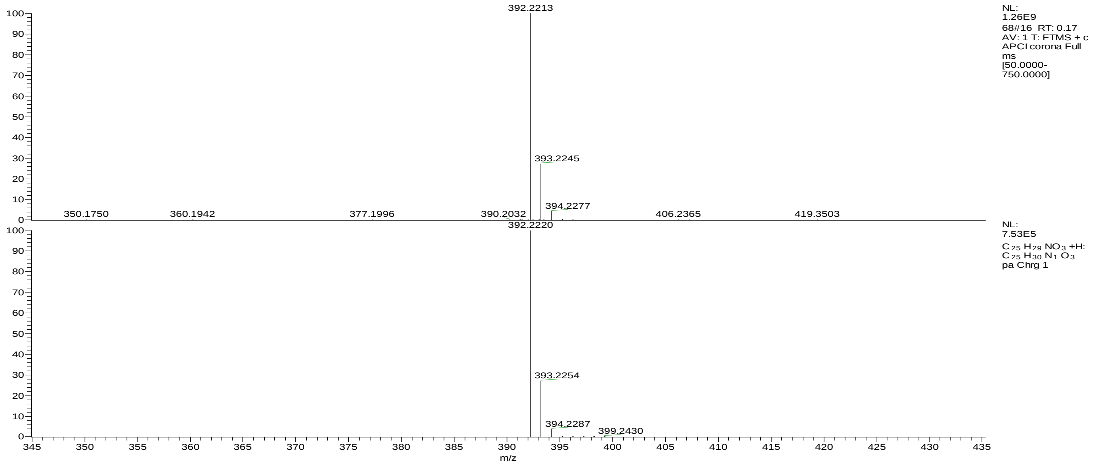

Figure S92. ESI-MS spectrum of intermediate **6v**.

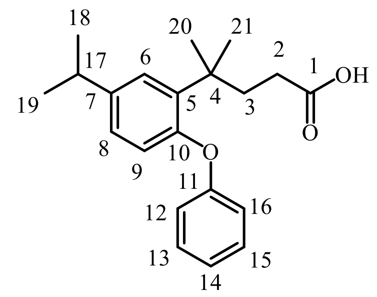

7a, white solid; yield 96.4%; m. p. 110.6 – 111.4 °C; FT-IR (KBr, cm^{-1}): 3039 (Ar–H), 2966, 2926, 2872 (C-H), 1705 (C=O), 1588, 1486, 1456, 1305, 1232 (Ar-C=C); ^{1}H NMR (500 MHz, $CDCl_3$) δ 7.38 – 7.25 (m, 2H, C_{13}-H, C_{15}-H), 7.13 (s, 1H, C_6-H), 7.04 (t, J = 7.4 Hz, 1H, C_{14}-H), 6.97 (dd, J = 14.5, 9.1 Hz, 3H, C_8-H C_{12}-H, C_{16}-H), 6.73 (d, J = 8.3 Hz, 1H, C_9-H), 2.87 (dt, J = 13.8, 6.9 Hz, 1H, C_{17}-H), 2.18 (d, J = 4.9 Hz, 2H, C_2-H), 2.12 (d, J = 4.9 Hz, 2H, C_3-H), 1.40 (s, 6H, C_{20}-H, C_{21}-H)), 1.24 (d, J = 6.9 Hz, 6H, C_{18}-H, C_{19}-H); ^{13}C NMR (126 MHz, $CDCl_3$) δ 180.36, 157.49, 153.47, 143.50, 137.06, 129.64, 126.52, 124.96, 122.60, 119.79, 118.56, 37.69, 35.91, 33.74, 30.56, 28.41, 24.20; ESI-MS m/z: 325.1808 [M + H^+].

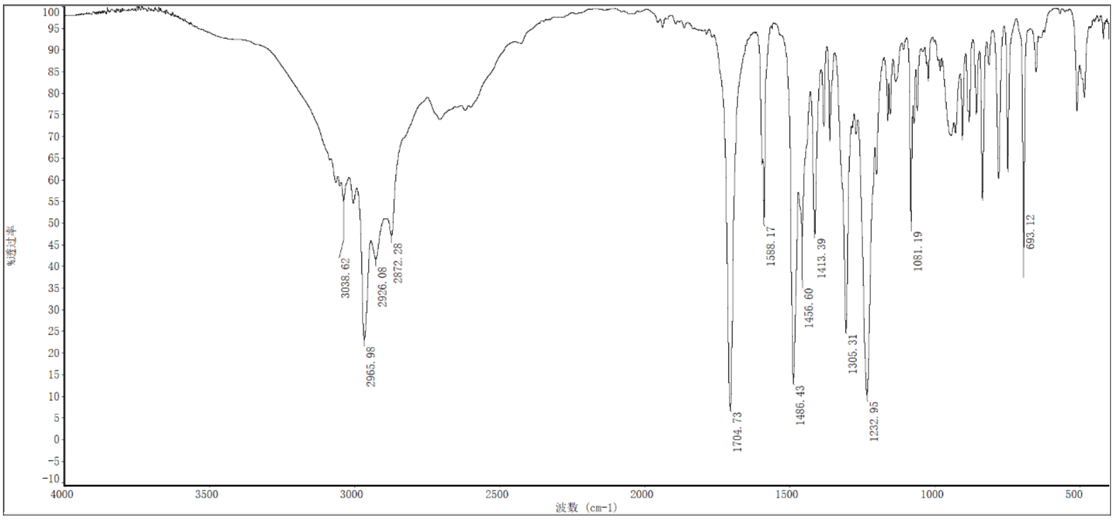

Figure S93. FT-IR spectrum of **7a**.

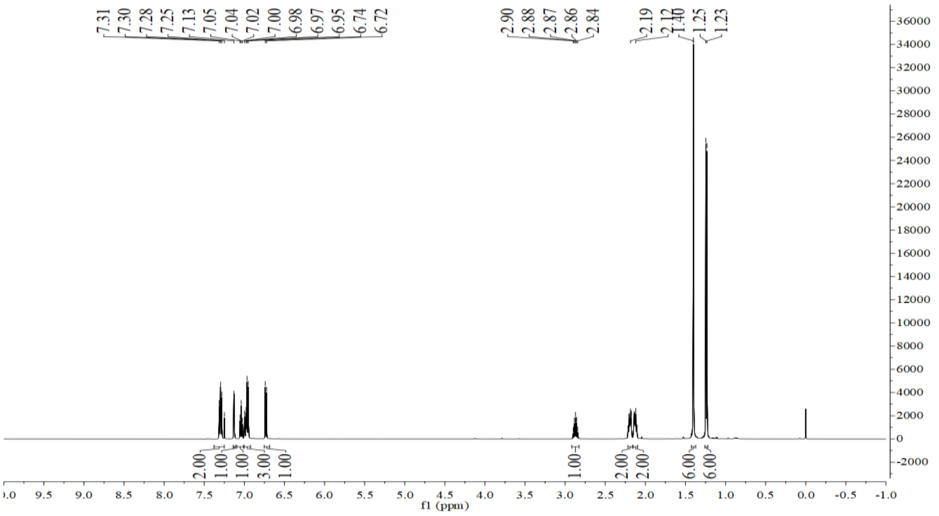

Figure S94. ^{1}H-NMR spectrum of **7a**.

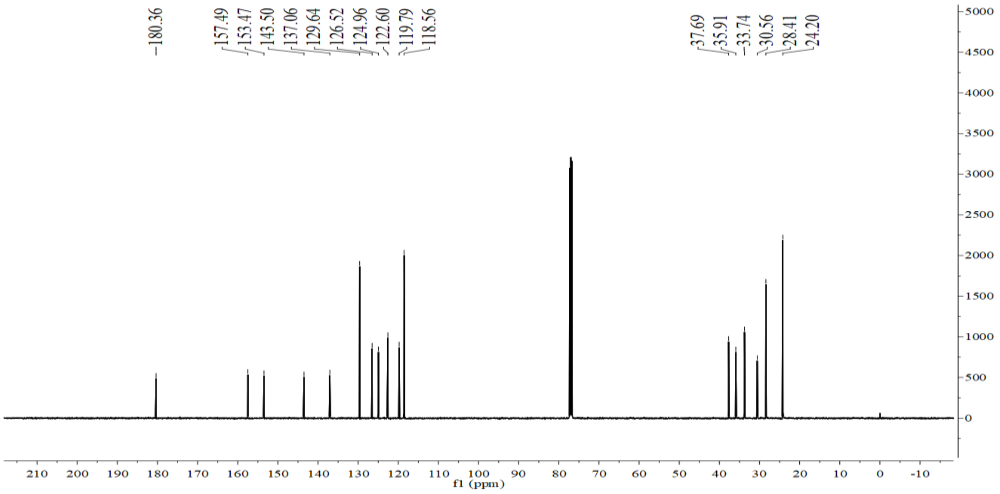

Figure S95. ^{13}C-NMR spectrum of **7a**.

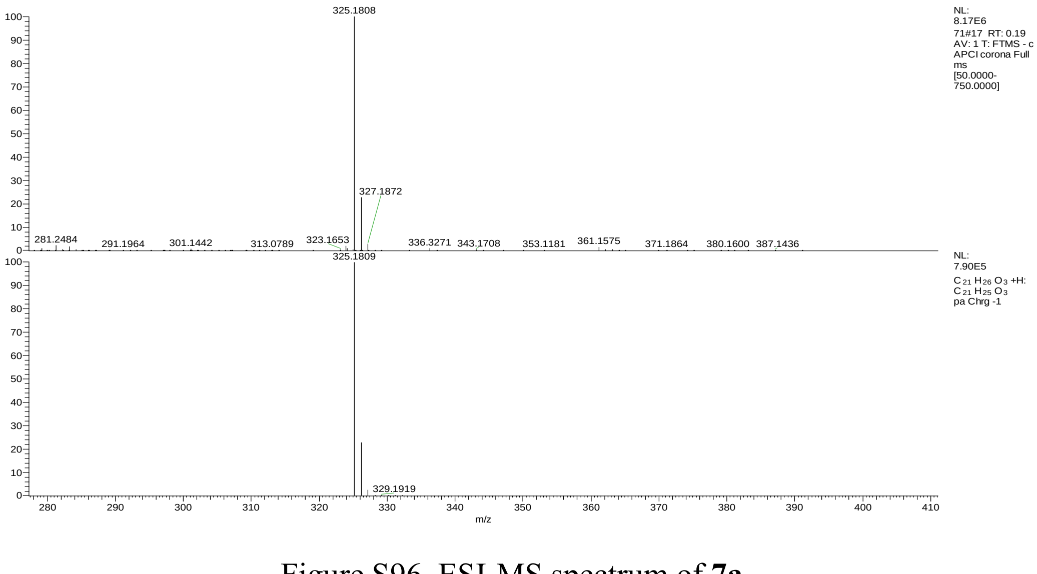

Figure S96. ESI-MS spectrum of **7a**.

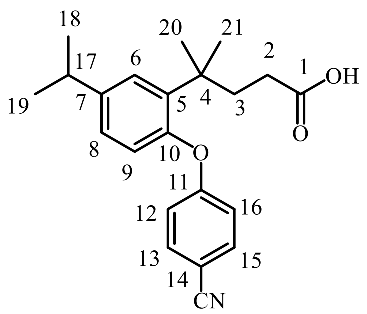

7b., white solid; yield 98.4%; m. p. 143.6– 144.8 °C; FT-IR (KBr, cm^{-1}): 3042 (Ar–H), 2966 (C-H), 2225 (C≡N), 1707 (C=O), 1595, 1489, 1457, 1307, 1230 (Ar-C=C); ^{1}H NMR (500 MHz, $CDCl_3$) δ 7.58 (d, J = 11.5 Hz, 2H, C_{13}-H, C_{15}-H), 7.18 (s, 1H, C_6-H), 7.07 (d, J = 10.4 Hz, 1H, C_8-H), 6.99 (d, J = 9.5 Hz, 2H, C_{12}-H, C_{16}-H), 6.79 (d, J = 8.2 Hz, 1H, C_9-H), 2.97 – 2.79 (m, 1H, C_{17}-H), 2.09 (s, 4H, C_2-H, C_3-H), 1.35 (s, 6H, C_{20}-H, C_{21}-H)), 1.26 (d, J = 6.9 Hz, 6H, C_{18}-H, C_{19}-H); ^{13}C NMR (126 MHz, $CDCl_3$) δ 179.96, 161.59, 151.20, 145.53, 138.14, 134.13, 127.09, 125.45, 121.41, 118.92, 117.92, 105.45, 37.68, 36.06, 33.85, 30.38, 28.47, 24.13; ESI-MS m/z: 350.1760 [M + H^+].

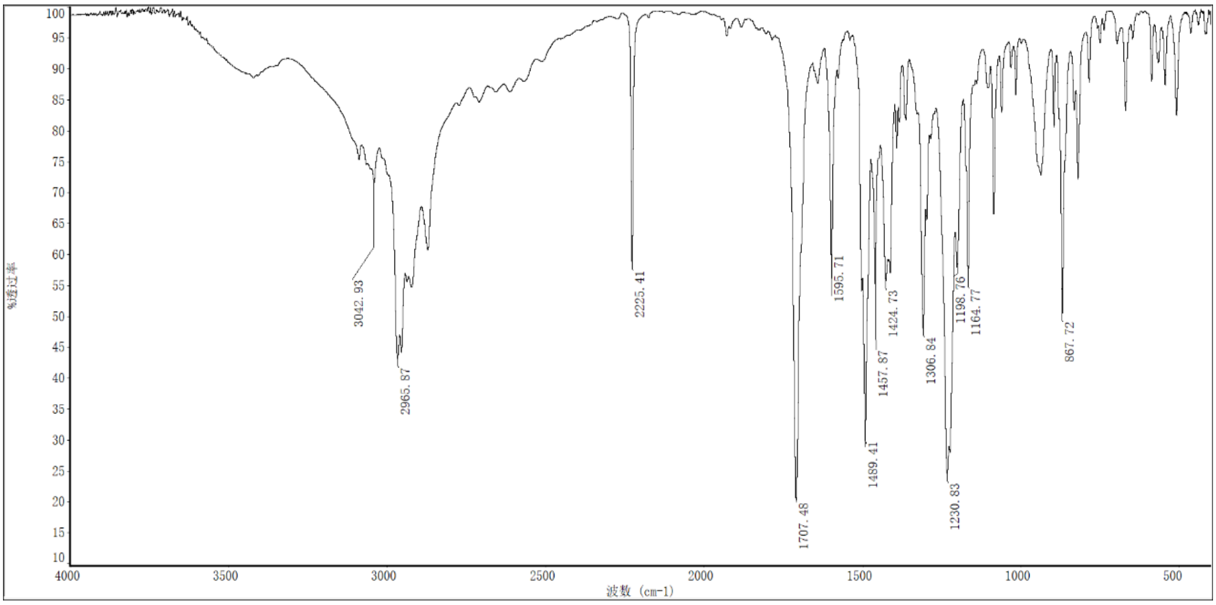

Figure S97. FT-IR spectrum of **7b**.

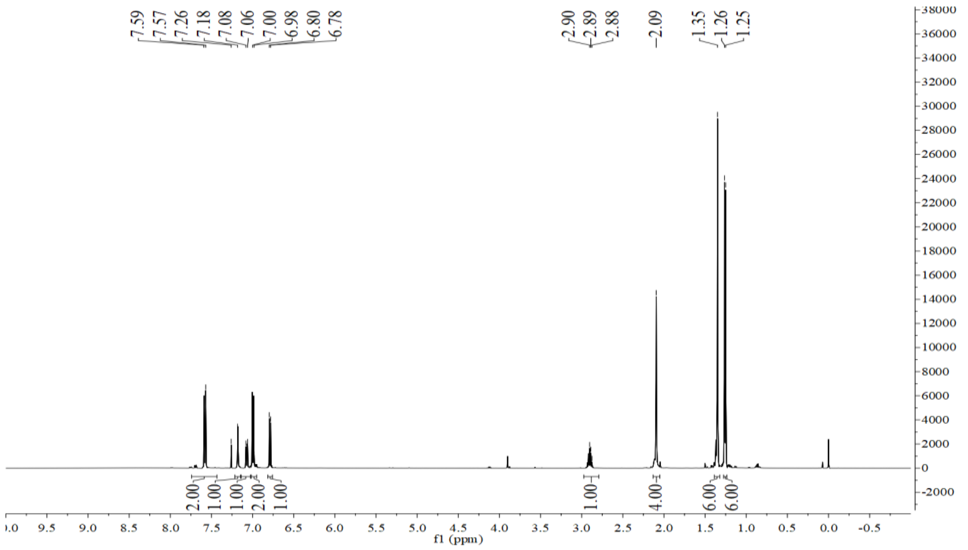

Figure S98. ^{1}H-NMR spectrum of **7b**.

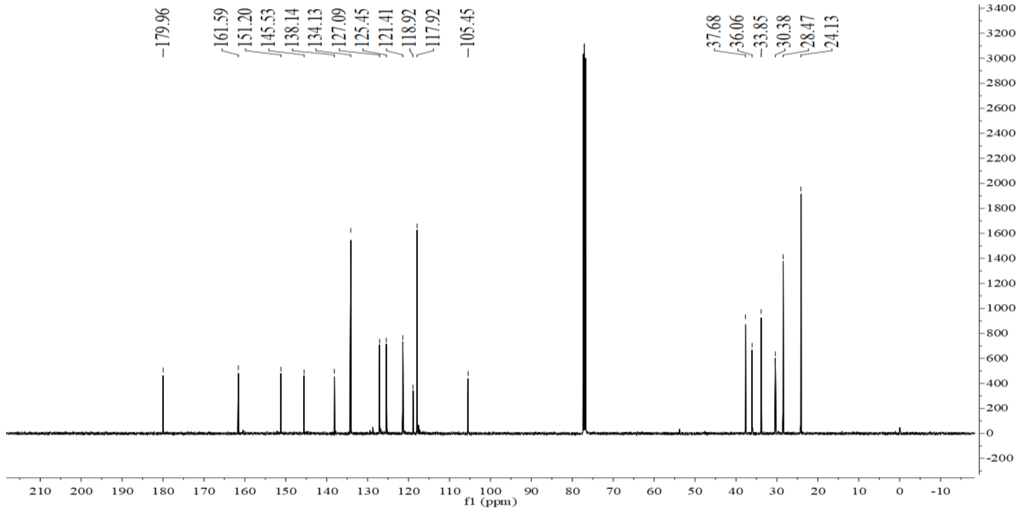

Figure S99. ^{13}C-NMR spectrum of **7b**.

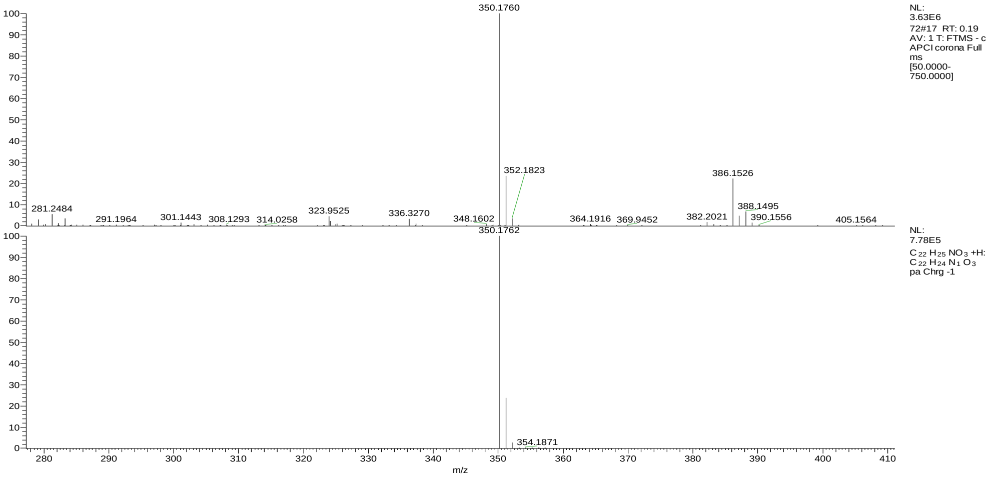

Figure S100. ESI-MS spectrum of **7b**.

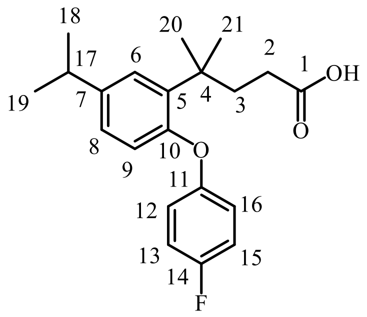

7c, white solid; yield 97.2%; m. p. 126.5– 127.8 °C; FT-IR (KBr, cm^{-1}): 3080 (Ar–H), 2960, 2872 (C-H), 1709 (C=O), 1504, 1488, 1457, 1293, 1209 (Ar-C=C); ^{1}H NMR (500 MHz, $CDCl_3$) δ 7.12 (d, *J* = 2.1 Hz, 1H, C_6-H), 6.98 (d, *J* = 8.2 Hz, 3H, C_8-H, C_{13}-H, C_{15}-H), 6.92 (d, *J* = 6.9 Hz, 2H, C_{12}-H, C_{16}-H), 6.67 (d, *J* = 8.3 Hz, 1H, C_9-H), 2.87 (dt, *J* = 13.8, 6.9 Hz, 1H, C_{17}-H), 2.19 (d, *J* = 9.1 Hz, 2H, C_2-H), 2.12 (d, *J* = 9.2 Hz, 2H, C_3-H), 1.40 (s, 6H, C_{20}-H, C_{21}-H), 1.23 (d, *J* = 6.9 Hz, 6H, C_{18}-H, C_{19}-H); ^{13}C NMR (126 MHz, $CDCl_3$) δ 180.27, 159.41, 157.50, 153.90, 153.22, 143.49, 136.76, 126.60, 125.02, 119.98, 119.09, 116.27, 116.08, 37.71, 35.88, 33.72, 30.55, 28.36, 24.19;ESI-MS m/z: 343.1714 [M + H^+].

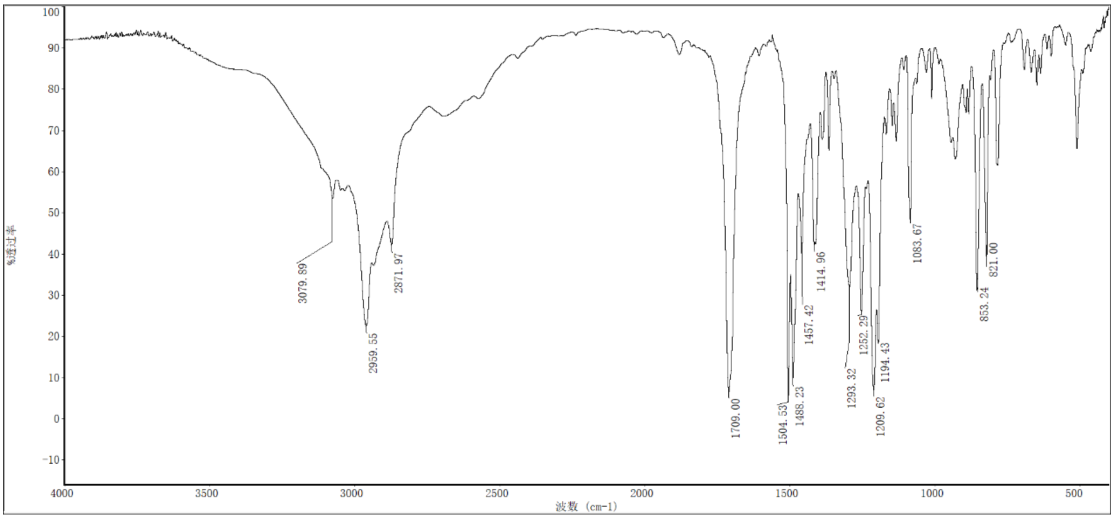

Figure S101. FT-IR spectrum of **7c**.

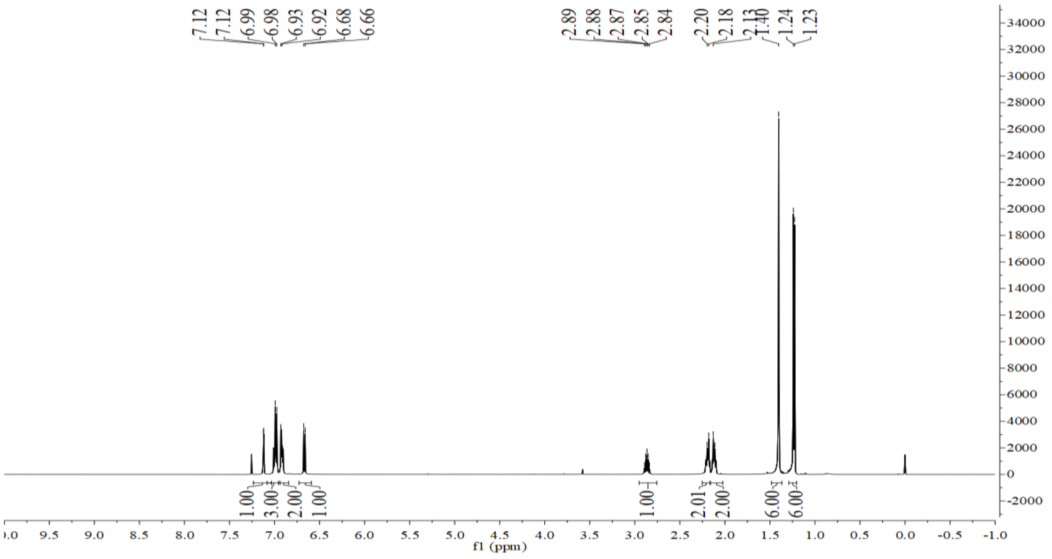

Figure S102. ^{1}H-NMR spectrum of **7c**.

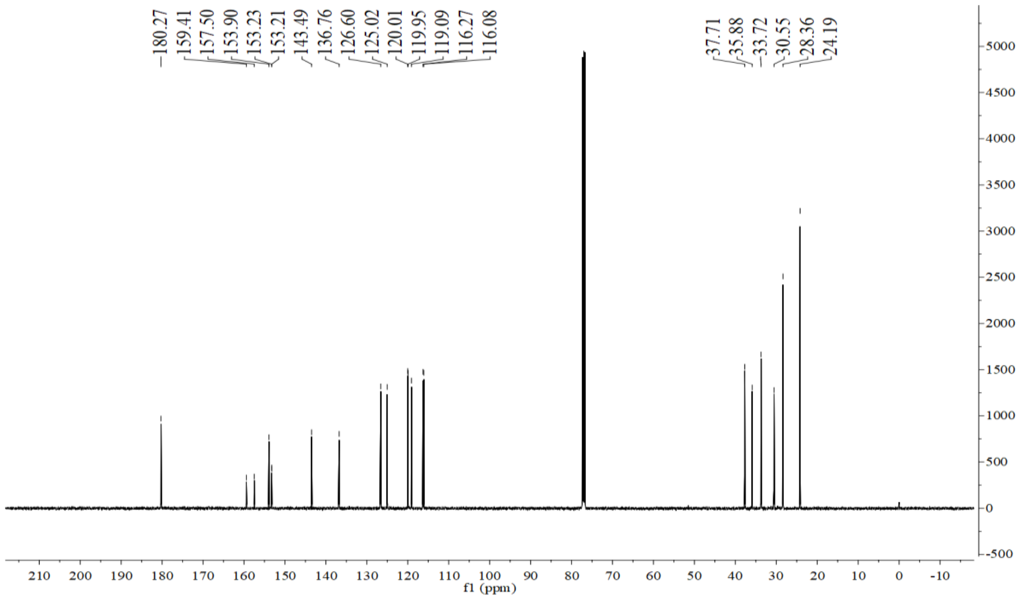

Figure S103. ^{13}C-NMR spectrum of **7c**.

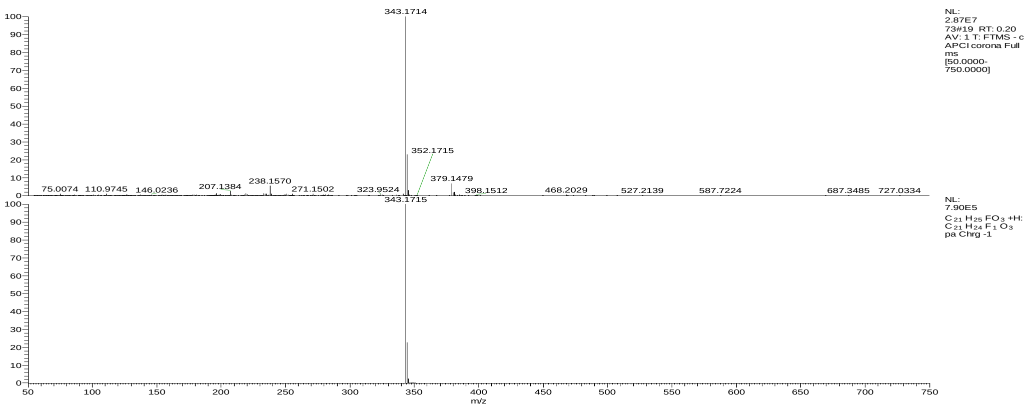

Figure S104. ESI-MS spectrum of **7c**.

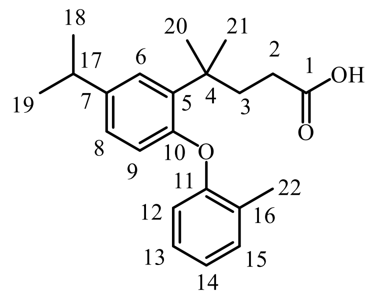

7d, white solid; yield 98.1%; m. p. 112.6– 113.9 °C; FT-IR (KBr, cm^{-1}): 3032 (Ar–H), 2954, 2923, 2869 (C-H), 1708 (C=O), 1485, 1460, 1413, 1304, 1237, 1208 (Ar-C=C); ^{1}H NMR (600 MHz, $CDCl_3$) δ 7.23 (d, J = 7.0 Hz, 1H, C_6-H), 7.13 – 7.10 (m, 2H, C_{13}-H, C_{15}-H), 7.00 (t, J = 7.8 Hz, 1H, C_{14}-H), 6.94 (d, J = 10.4 Hz, 1H, C_8-H), 6.79 (d, J = 8.0 Hz, 1H, C_9-H), 6.54 (d, J = 8.3 Hz, 1H, C_{12}-H), 2.86 (dt, J = 13.8, 6.9 Hz, 1H, C_{17}-H), 2.28 (s, 3H, C_{22}-H), 2.25 (d, J = 8.9 Hz, 2H, C_2-H), 2.14 (dd, J = 9.3, 7.3 Hz, 2H, C_3-H), 1.44 (s, 6H, C_{20}-H, C_{21}-H), 1.23 (d, J = 6.9 Hz, 6H, C_{18}-H, C_{19}-H); ^{13}C NMR (151 MHz, $CDCl_3$) δ 180.33, 154.88, 154.04, 142.63, 131.21, 127.09 , 126.51, 124.86, 123.19, 118.66, 117.62, 37.77, 35.89, 33.67, 30.64, 28.36, 24.22, 16.35; ESI-MS m/z: 339.1964 [M + H^+].

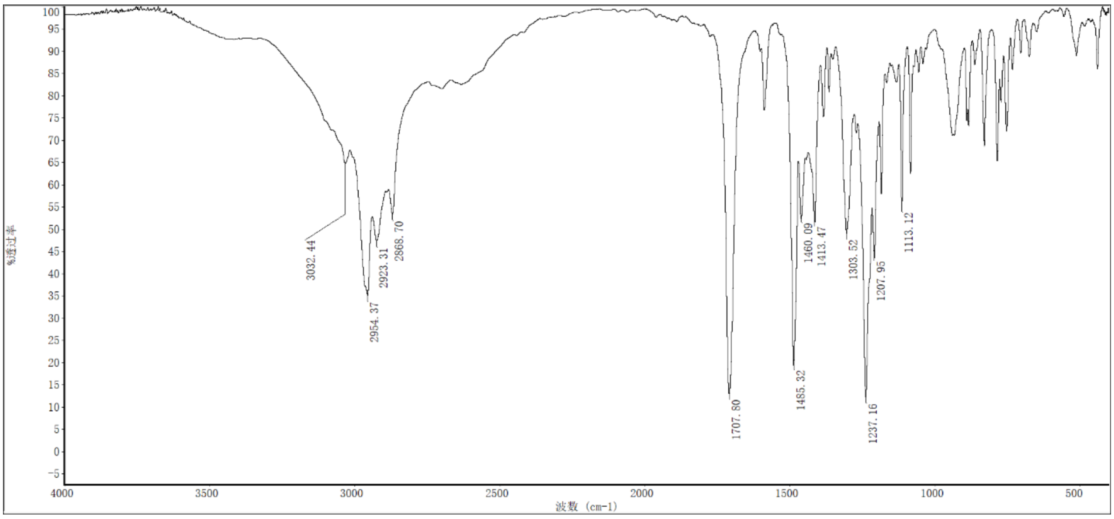

Figure S105. FT-IR spectrum of **7d**.

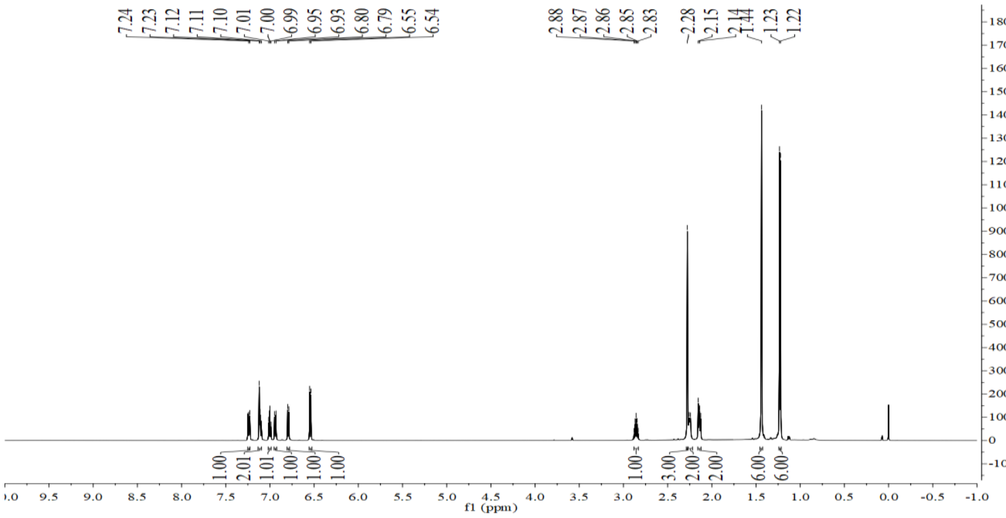

Figure S106. ^{1}H-NMR spectrum of **7d**.

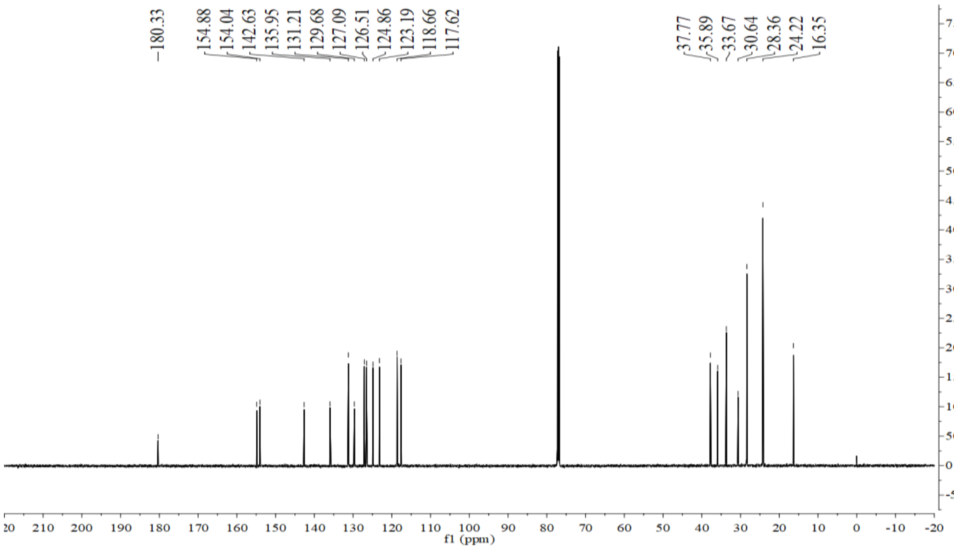

Figure S107. ^{13}C-NMR spectrum of **7d**.

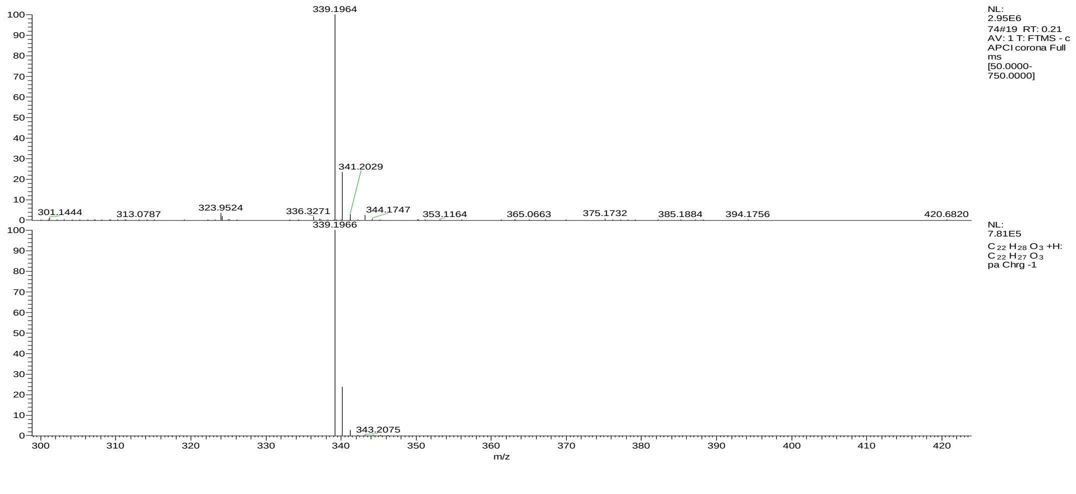

Figure S108. ESI-MS spectrum of **7d**.

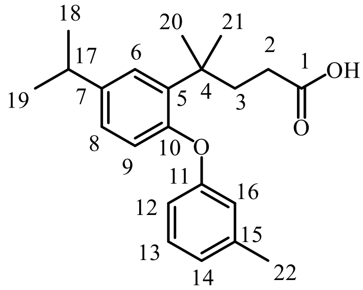

7e, white solid; yield 95.7%; m. p. 110.5– 112.0 °C; FT-IR (KBr, cm^{-1}): 2961, 2871, 2869 (C-H), 1709 (C=O), 1591, 1485, 1410, 1307, 1259, 1229 (Ar-C=C); ^{1}H NMR (600 MHz, $CDCl_3$) δ 7.18 (t, *J* = 7.8 Hz, 1H, C_{13}-H), 7.13 (d, *J* = 2.2 Hz, 1H, C_6-H), 6.99 (dd, *J* = 8.3, 2.1 Hz, 1H, C_8-H), 6.86 (d, *J* = 7.5 Hz, 1H, C_{16}-H), 6.80 (s, 1H, C_{14}-H), 6.74 (t, *J* = 10.3 Hz, 2H, C_9-H C_{12}-H), 2.87 (dt, *J* = 13.8, 6.9 Hz, 1H, C_{17}-H), 2.31 (s, 3H, C_{22}-H), 2.22 – 2.18 (m, 2H, C_2-H), 2.15 – 2.10 (m, 2H, C_3-H), 1.41 (s, 6H, C_{20}-H, C_{21}-H), 1.24 (d, *J* = 6.9 Hz, 6H, C_{18}-H, C_{19}-H); ^{13}C NMR (151 MHz, $CDCl_3$) δ 180.51, 157.40, 153.60, 143.32, 139.83, 136.94, 129.34, 126.48, 124.93, 123.48, 119.69, 119.32, 115.58, 37.69, 35.89, 33.73, 30.5 , 28.40, 24.21, 21.38; ESI-MS m/z: 339.1963 [M + H^+].

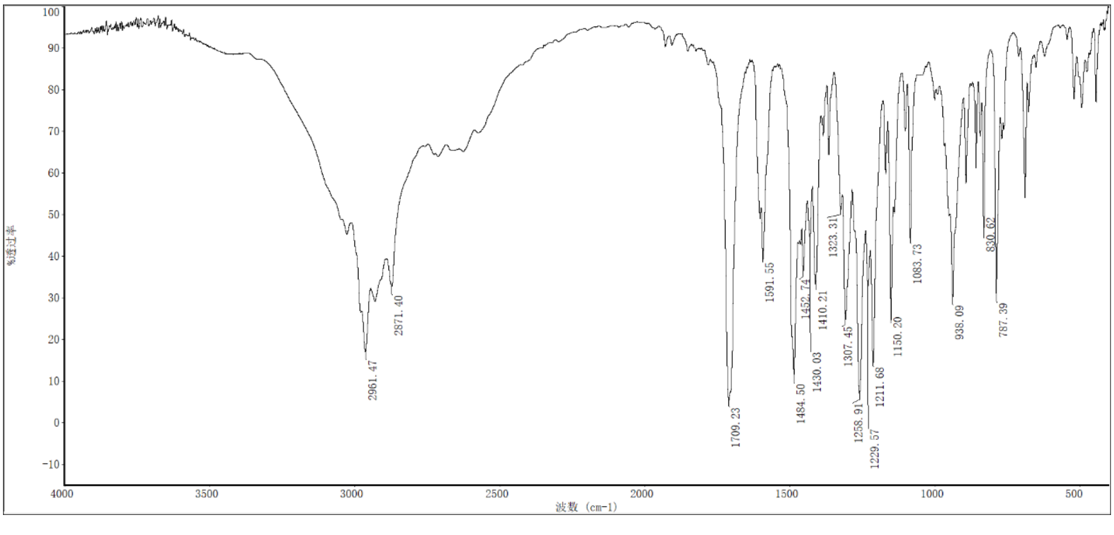

Figure S109. FT-IR spectrum of **7e**.

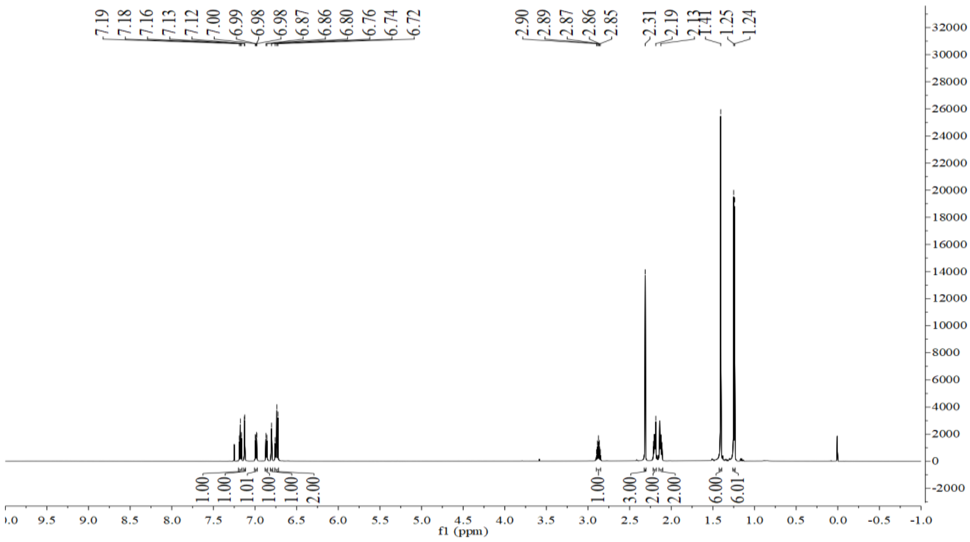

Figure S110. ^{1}H-NMR spectrum of **7e**.

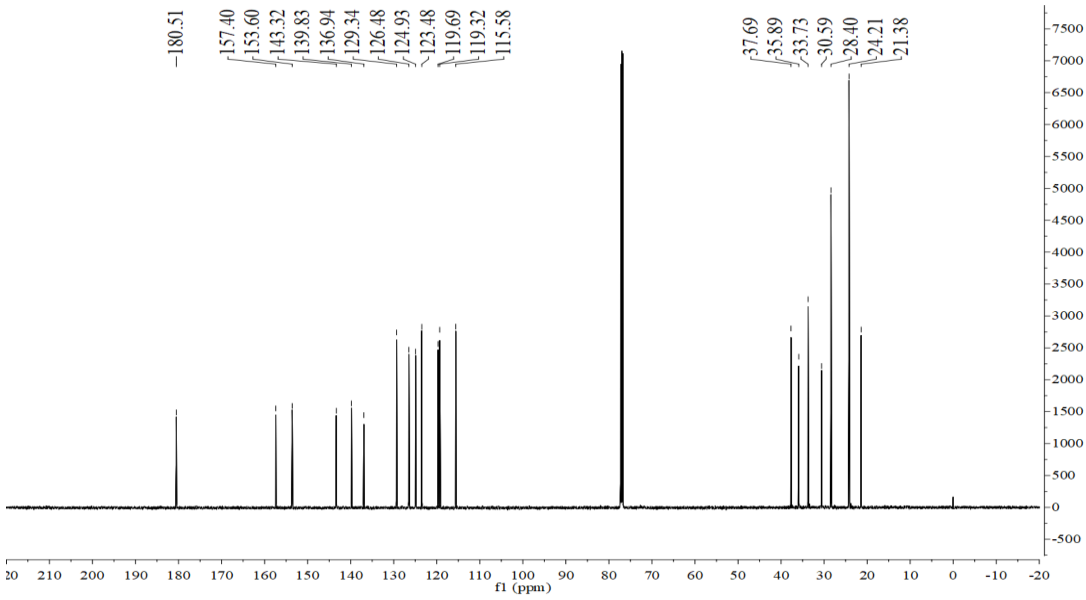

Figure S111. ^{13}C-NMR spectrum of **7e**.

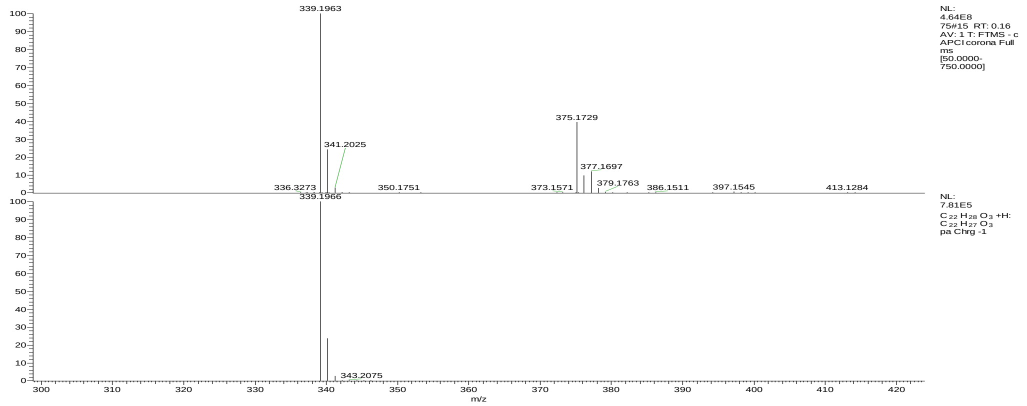

Figure S112. ESI-MS spectrum of **7e**.

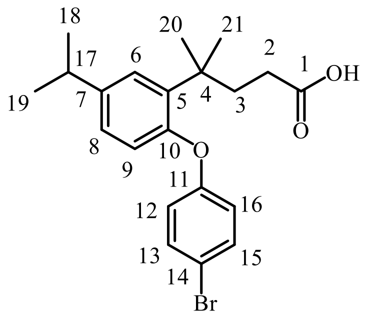

7f, white solid; yield 98.2%; m. p. 133.3– 134.6 °C; FT-IR (KBr, cm^{-1}): 2961, 2871, 2869 (C-H), 1708 (C=O), 1481, 1411, 1241, 1215 (Ar-C=C); ^{1}H NMR (600 MHz, $CDCl_3$) δ 7.39 (d, J = 8.9 Hz, 2H, C_{13}-H, C_{15}-H), 7.13 (s, 1H, C_6-H), 7.01 (d, J = 10.4 Hz, 1H, C_8-H), 6.84 (d, J = 2.1 Hz, 2H, C_{12}-H, C_{16}-H), 6.73 – 6.71 (m, 1H, C_9-H), 2.87 (dt, J = 13.8, 6.9 Hz, 1H, C_{17}-H), 2.15 (d, J = 9.3 Hz, 2H, C_2-H), 2.13 – 2.09 (m, 2H, C_3-H), 1.38 (s, 6H, C_{20}-H, C_{21}-H), 1.24 (d, J = 6.9 Hz, 6H, C_{18}-H, C_{19}-H); ^{13}C NMR (151 MHz, $CDCl_3$) δ 180.14, 156.82, 152.91, 144.14, 138.56, 137.33, 132.58, 126.71, 125.15, 120.48, 120.04, 114.94, 37.68, 35.93, 33.76, 30.48, 28.41, 24.17; ESI-MS m/z: 403.0910 [M + H^+].

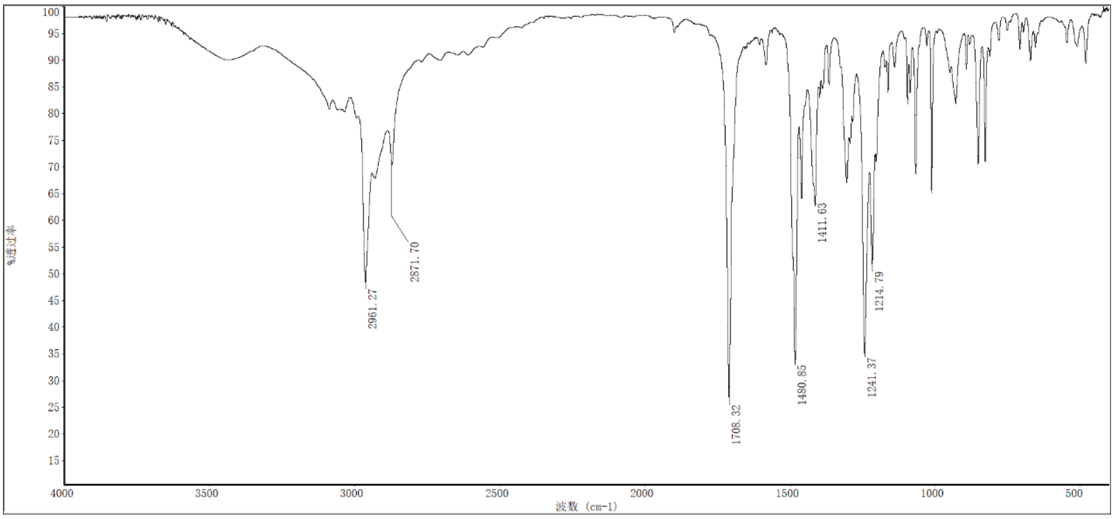

Figure S113. FT-IR spectrum of **7f**.

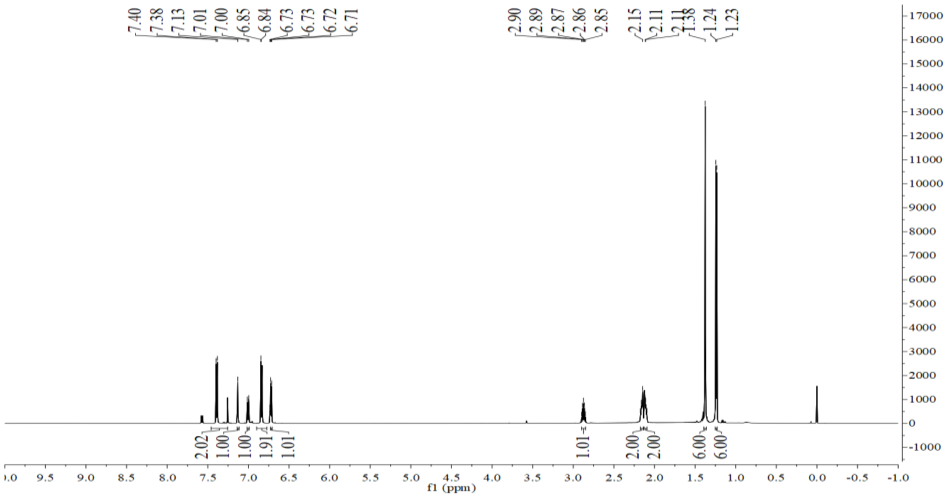

Figure S114. ^{1}H-NMR spectrum of **7f**.

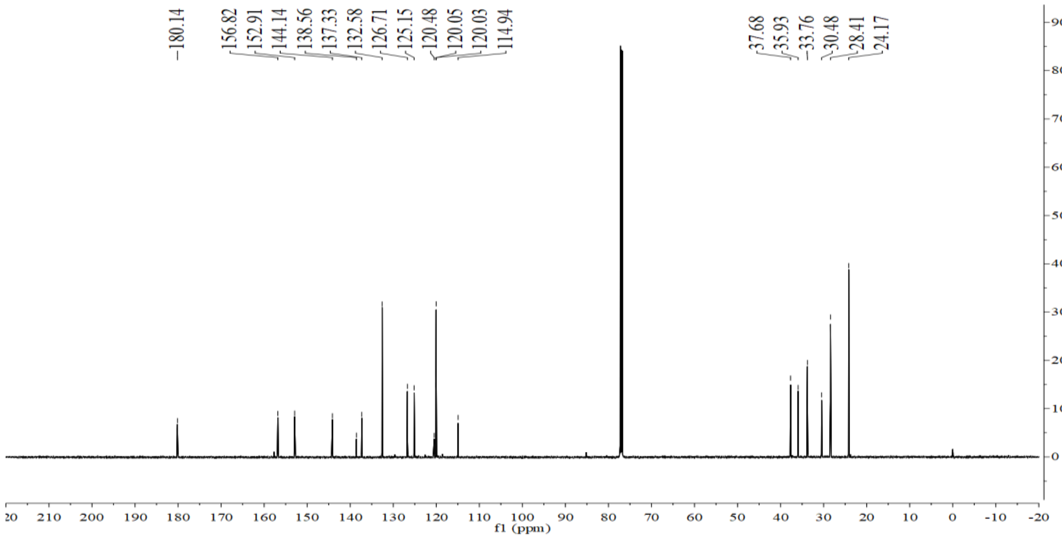

Figure S115. ^{13}C-NMR spectrum of **7f**.

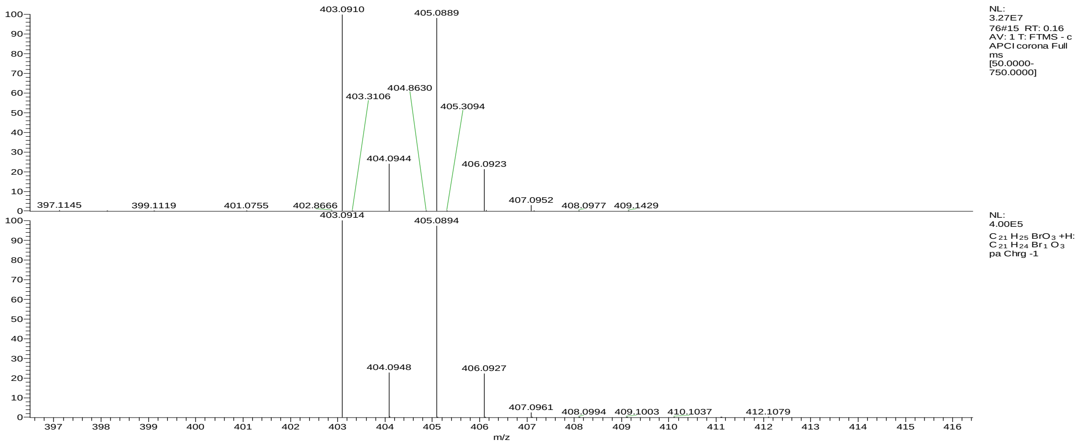

Figure S116. ESI-MS spectrum of **7f**.

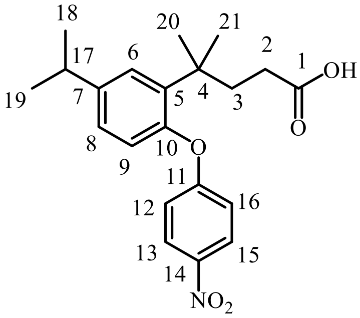

7g, white solid; yield 94.8%; m. p. 133.6– 134.9 °C; FT-IR (KBr, cm^{-1}): 2961, 2923 (C-H), 1702 (C=O), 1588, 1514, 1487, 1347, 1253 (Ar-C=C); ^{1}H NMR (600 MHz, $CDCl_3$) δ 8.19 (d, *J* = 9.2 Hz, 2H, C_{13}-H, C_{15}-H), 7.20 (d, *J* = 2.0 Hz, 1H, C_6-H), 7.10 (d, *J* = 8.2 Hz, 1H, C_8-H), 7.01 (t, *J* = 6.1 Hz, 2H, C_{12}-H, C_{16}-H), 6.82 (d, *J* = 8.2 Hz, 1H, C_9-H), 2.92 (dt, *J* = 13.8, 6.9 Hz, 1H, C_{17}-H), 2.09 (s, 4H, C_2-H, C_3-H), 1.36 (s, 6H, C_{20}-H, C_{21}-H), 1.27 (d, *J* = 6.9 Hz, 6H, C_{18}-H, C_{19}-H); ^{13}C NMR (151 MHz, $CDCl_3$) δ 179.96, 163.31, 151.13, 145.86, 142.47, 138.21, 127.20, 125.99, 125.57, 121.62, 117.11, 37.70, 36.03, 33.88, 30.30, 28.49, 24.13; ESI-MS m/z: 370.1659 [M + H^+].

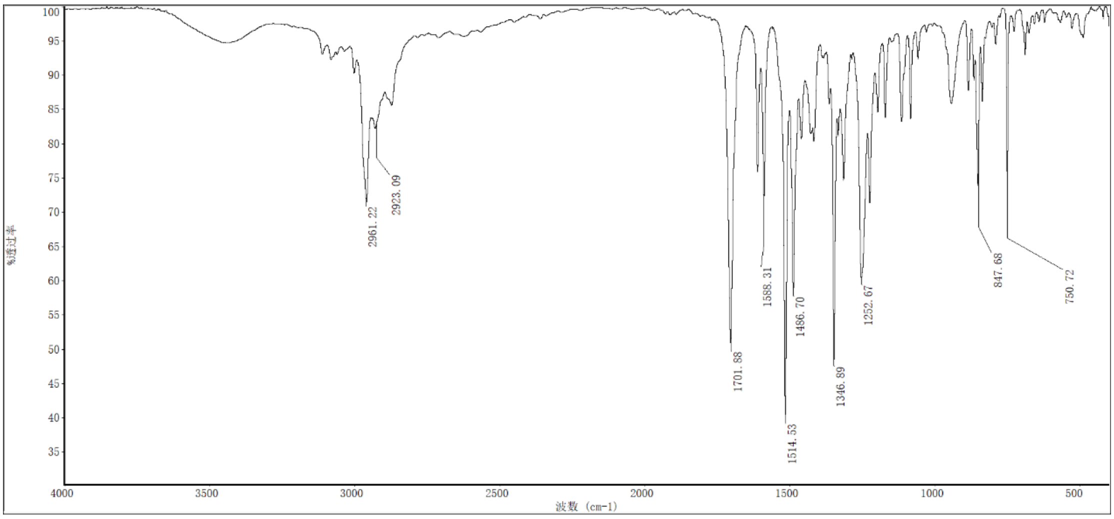

Figure S117. FT-IR spectrum of **7g**.

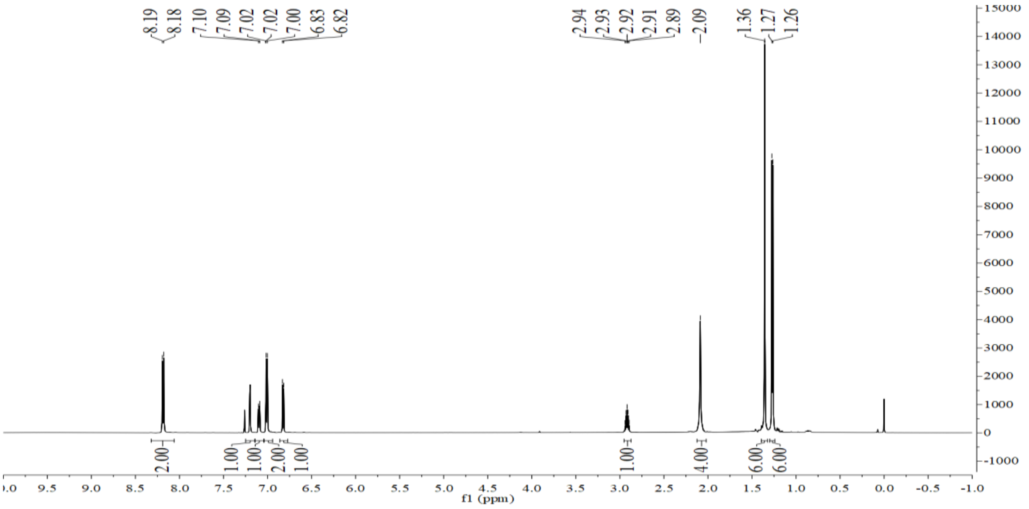

Figure S118. ^{1}H-NMR spectrum of **7g**.

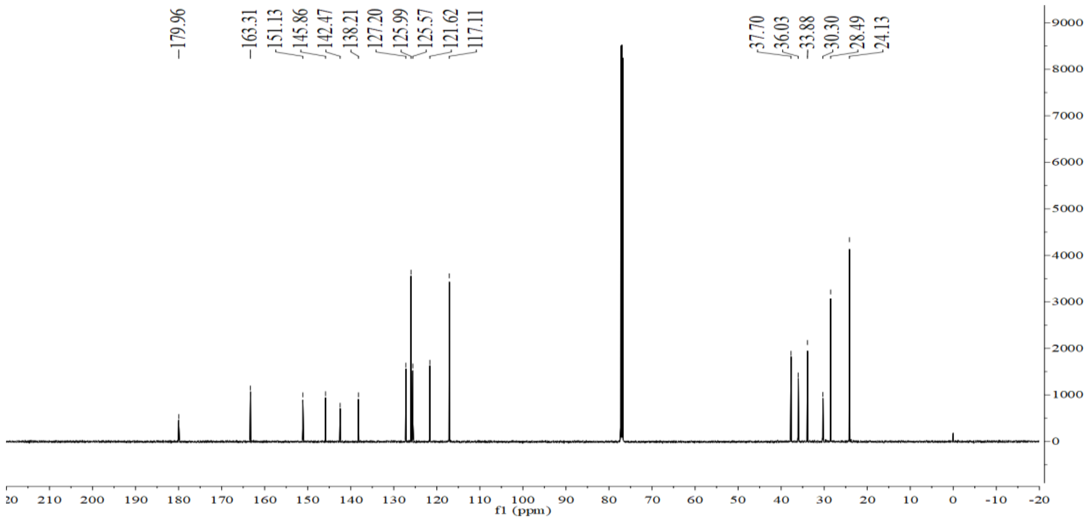

Figure S119. ^{13}C-NMR spectrum of **7g**.

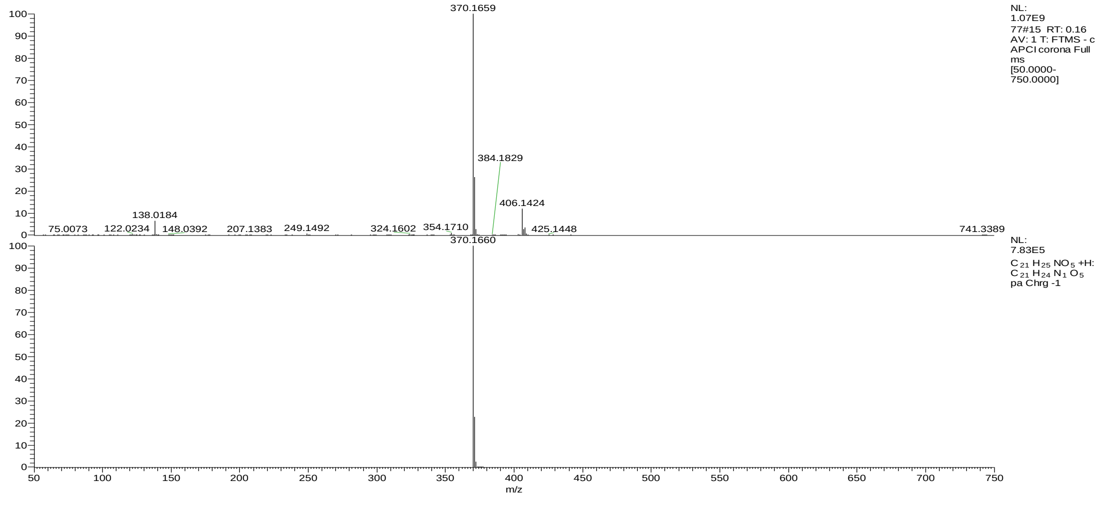

Figure S120. ESI-MS spectrum of **7g**.

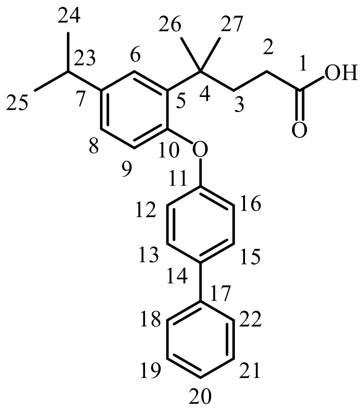

7h, white solid; yield 96.5%; m. p. 148.8– 150.2 °C; FT-IR (KBr, cm^{-1}): 3030 (Ar–H), 2960, 2867 (C-H), 1705 (C=O), 1486, 1411, 1299, 1244, 1167 (Ar-C=C); ^{1}H NMR (600 MHz, $CDCl_3$) δ 7.53 (dd, J = 17.8, 8.1 Hz, 4H, C_{12}-H, C_{13}-H, C_{15}-H, C_{16}-H), 7.40 (t, J = 7.6 Hz, 2H, C_{19}-H, C_{21}-H), 7.29 (s, 1H, C_8-H), 7.14 (s, 1H, C_{20}-H), 7.02 (d, J = 8.5 Hz, 3H, C_6-H, C_{18}-H, C_{22}-H), 6.80 (d, J = 8.3 Hz, 1H, C_9-H), 2.88 (dt, J = 13.8, 6.9 Hz, 1H, C_{23}-H), 2.23 – 2.16 (m, 2H, C_2-H), 2.14 – 2.08 (m, 2H, C_3-H), 1.41 (s, 6H, C_{26}-H, C_{27}-H), 1.25 (d, J = 6.9 Hz, 6H, C_{24}-H, C_{25}-H); ^{13}C NMR (151 MHz, $CDCl_3$) δ 180.47, 157.15, 153.37, 143.76, 140.67, 137.25, 135.66, 128.75, 128.38, 126.90, 126.63, 125.08, 120.10, 118.69, 37.75, 35.95, 33.81, 30.58, 28.49, 24.25; ESI-MS m/z: 401.2119 [M + H^+].

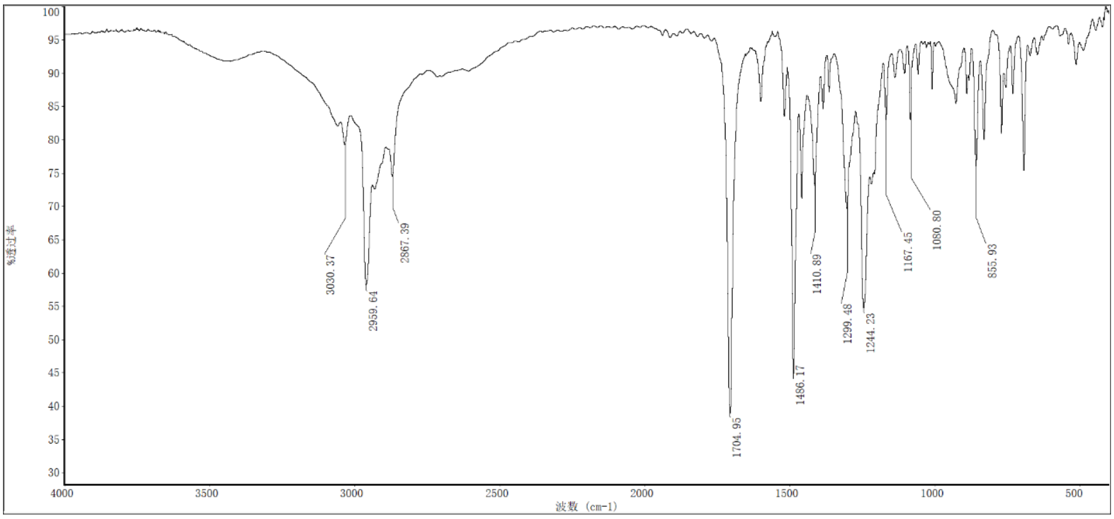

Figure S121. FT-IR spectrum of **7h**.

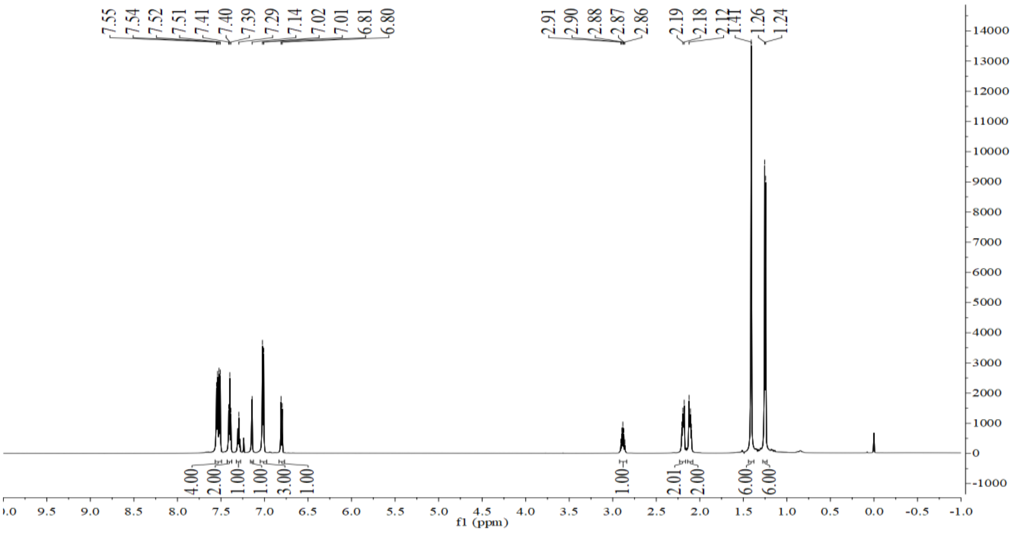

Figure S122. ^{1}H-NMR spectrum of **7h**.

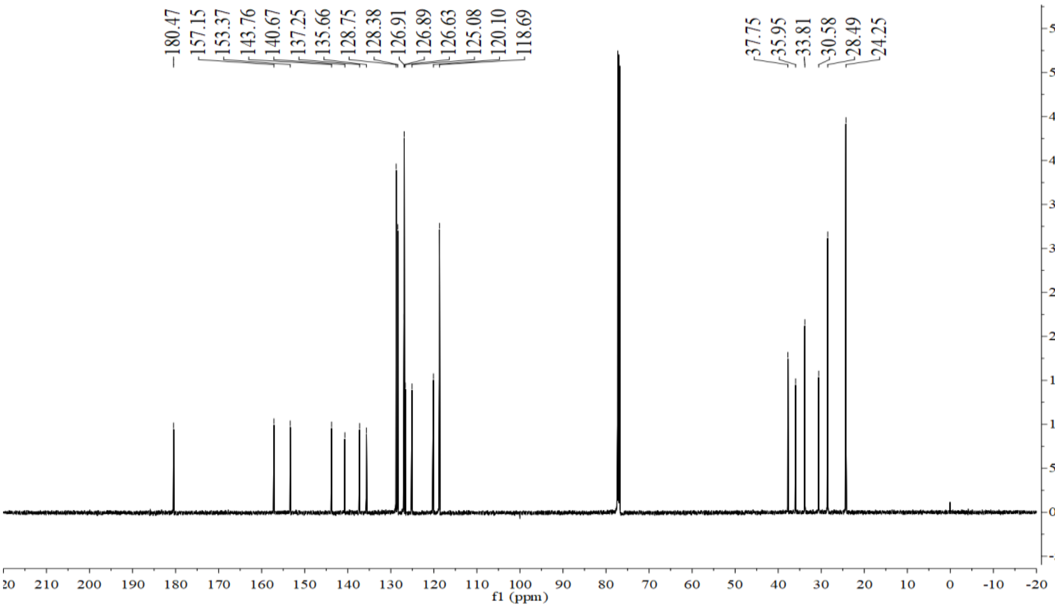

Figure S123. ^{13}C-NMR spectrum of **7h**.

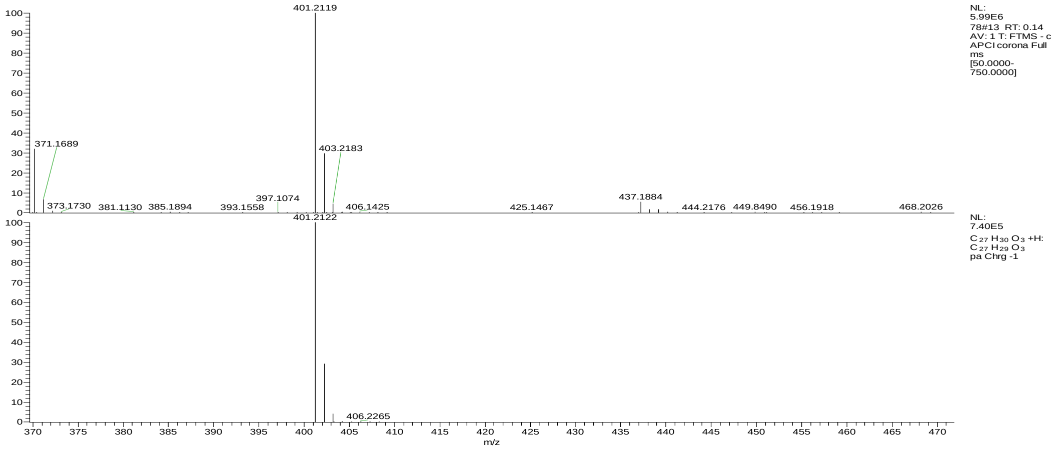

Figure S124. ESI-MS spectrum of **7h**.

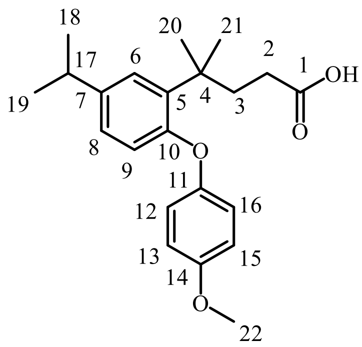

7i, white solid; yield 90.4%; m. p. 121.5– 123.1 °C; FT-IR (KBr, cm^{-1}): 2959, 2872 (C-H), 1711 (C=O), 1506, 1489, 1455, 1421, 1303, 1249, 1224, 1220 (Ar-C=C); ^{1}H NMR (600 MHz, $CDCl_3$) δ 7.10 (d, J = 2.1 Hz, 1H, C_6-H), 6.95 (dd, J = 8.3, 2.1 Hz, 1H, C_8-H), 6.91 (d, J = 9.0 Hz, 2H, C_{13}-H, C_{15}-H), 6.85 (d, J = 9.1 Hz, 2H, C_{12}-H, C_{16}-H), 6.64 (d, J = 8.3 Hz, 1H, C_9-H), 3.78 (s, 3H, C_{22}-H), 2.85 (dt, J = 13.8, 6.9 Hz, 1H, C_{17}-H), 2.25 – 2.21 (m, 2H, C_2-H), 2.15 – 2.10 (m, 2H, C_3-H), 1.42 (s, 6H, C_{20}-H, C_{21}-H), 1.22 (d, J = 6.9 Hz, 6H, C_{18}-H, C_{19}-H); ^{13}C NMR (151 MHz, $CDCl_3$) δ 180.50, 155.38, 154.70, 150.64, 142.72, 136.23, 126.41, 124.83, 120.27, 118.28, 114.81, 55.64, 37.71, 33.67, 30.63, 28.33, 24.21; ESI-MS m/z: 355.1913 [M + H^+].

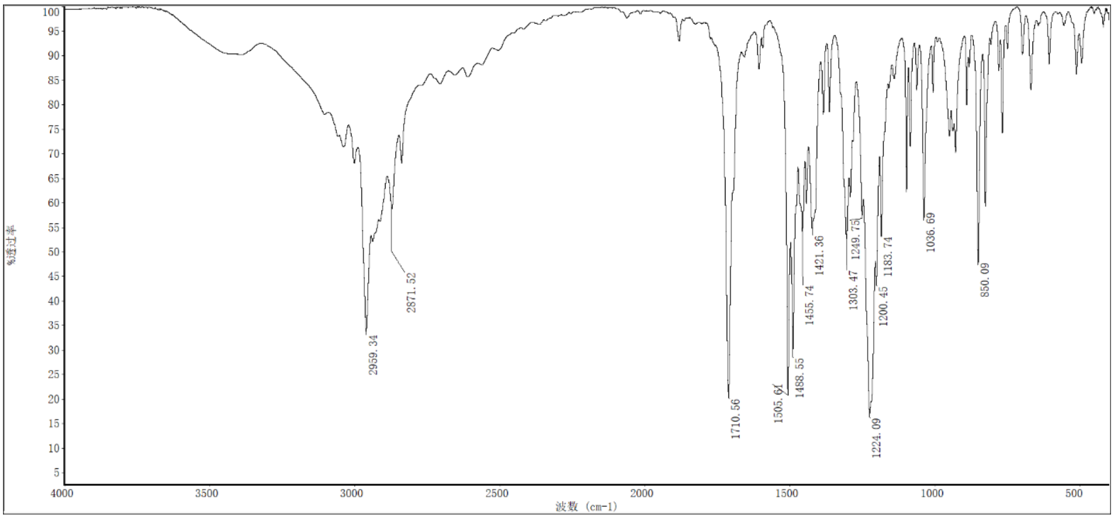

Figure S125. FT-IR spectrum of **7i**.

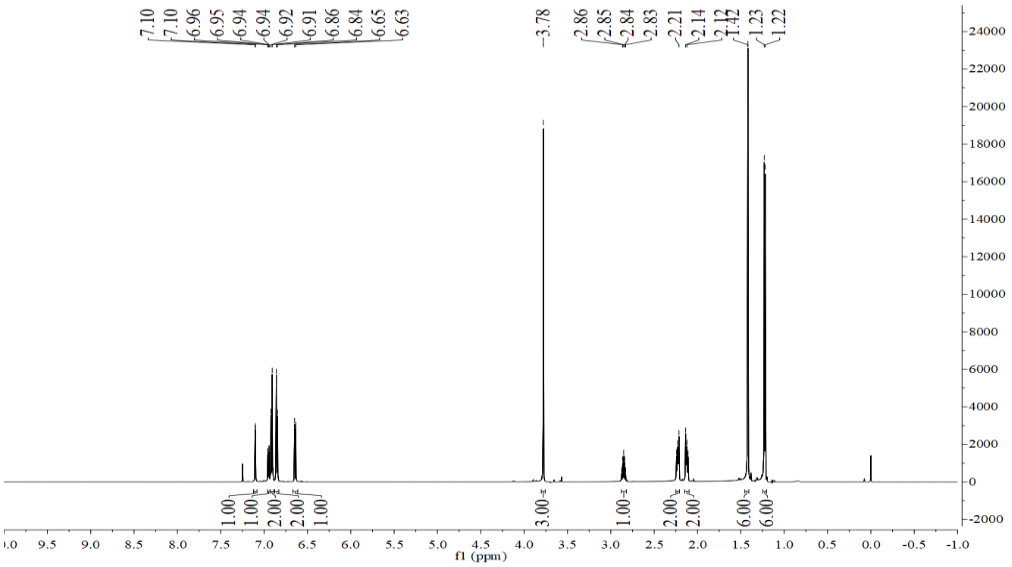

Figure S126. ^{1}H-NMR spectrum of **7i**.

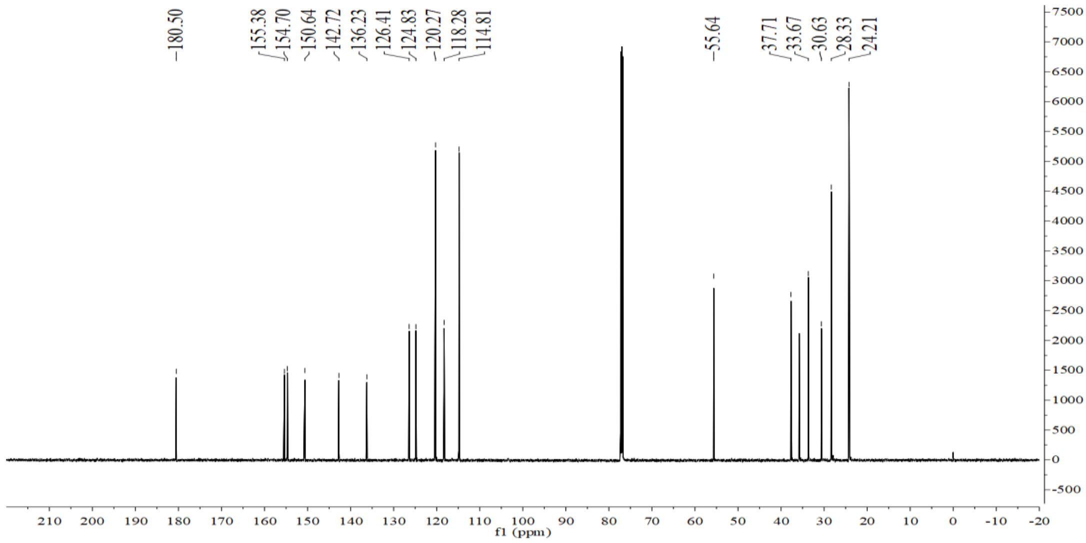

Figure S127. ^{13}C-NMR spectrum of **7i**.

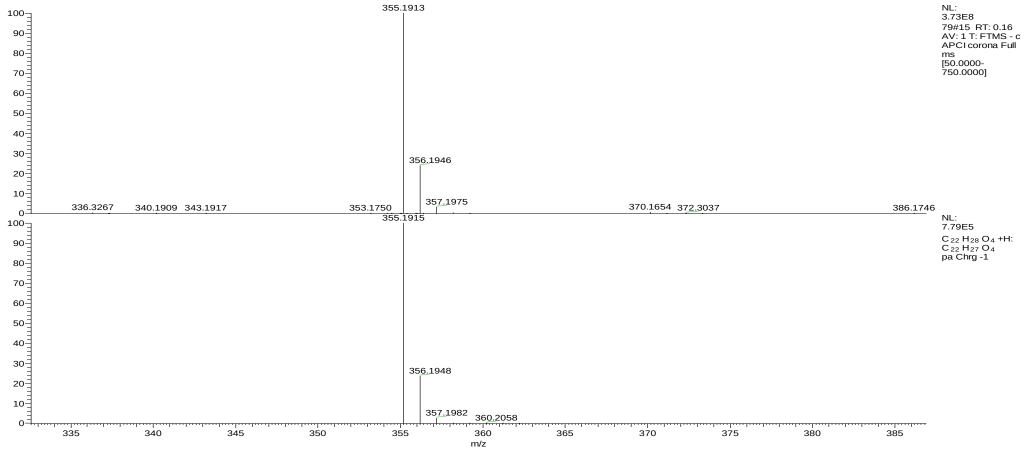

Figure S128. ESI-MS spectrum of **7i**.

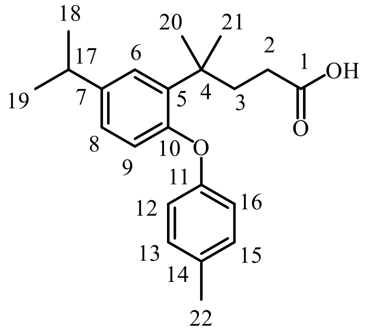

7j, white solid; yield 96.3%; m. p. 113.6– 114.8 °C; FT-IR (KBr, cm^{-1}): 2959, (C-H), 1701 (C=O), 1506, 1488, 1456, 1413, 1381, 1306, 1232, 1210 (Ar-C=C); ^{1}H NMR (600 MHz, $CDCl_3$) δ 7.12 – 6.94 (m, 3H, C_6-H, C_{13}-H, C_{15}-H), 6.89 (d, J = 8.3 Hz, 1H, C_8-H), 6.78 (d, J = 6.6 Hz, 2H, C_{12}-H, C_{16}-H), 6.62 (d, J = 8.3 Hz, 1H, C_9-H), 2.78 (dt, J = 13.8, 6.9 Hz, 1H, C_{17}-H), 2.23 (s, 3H, C_{22}-H), 2.15 – 2.10 (m, 2H, C_2-H), 2.07 – 2.00 (m, 2H, C_3-H), 1.33 (s, 6H, C_{20}-H, C_{21}-H), 1.16 (d, J = 6.9 Hz, 6H, C_{18}-H, C_{19}-H); ^{13}C NMR (151 MHz, $CDCl_3$) δ 180.57, 155.09, 153.96, 143.11, 136.74, 132.11, 130.13, 126.43, 124.88, 119.24, 118.65, 37.68, 35.85, 33.71, 30.61, 28.38, 24.21, 20.64; ESI-MS m/z: 339.1963 [M + H^+].

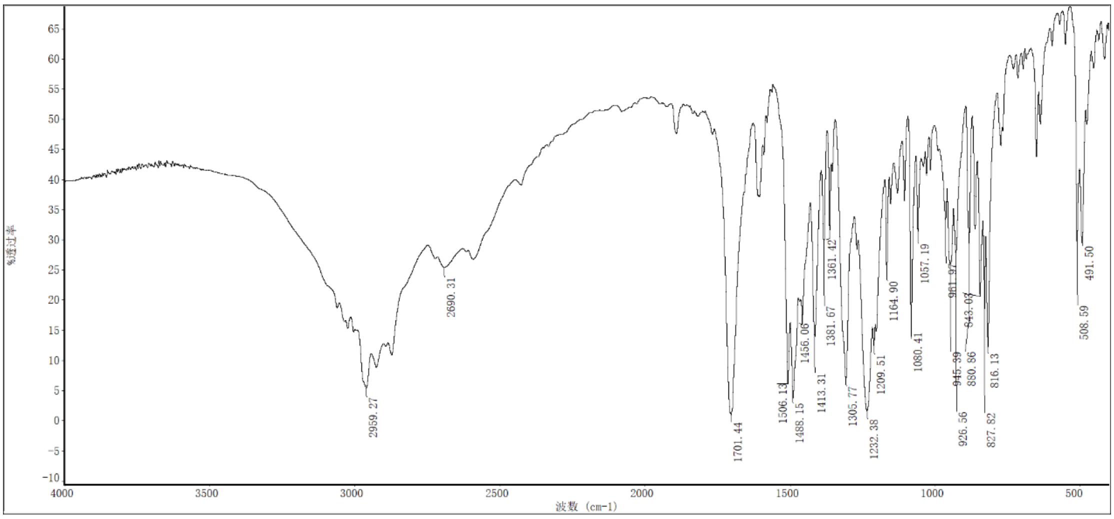

Figure S129. FT-IR spectrum of **7j**.

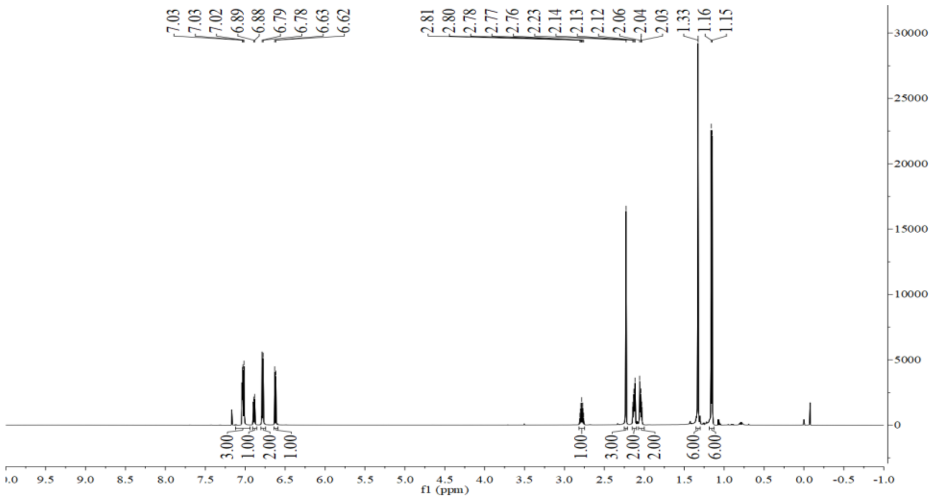

Figure S130. ^{1}H-NMR spectrum of **7j**.

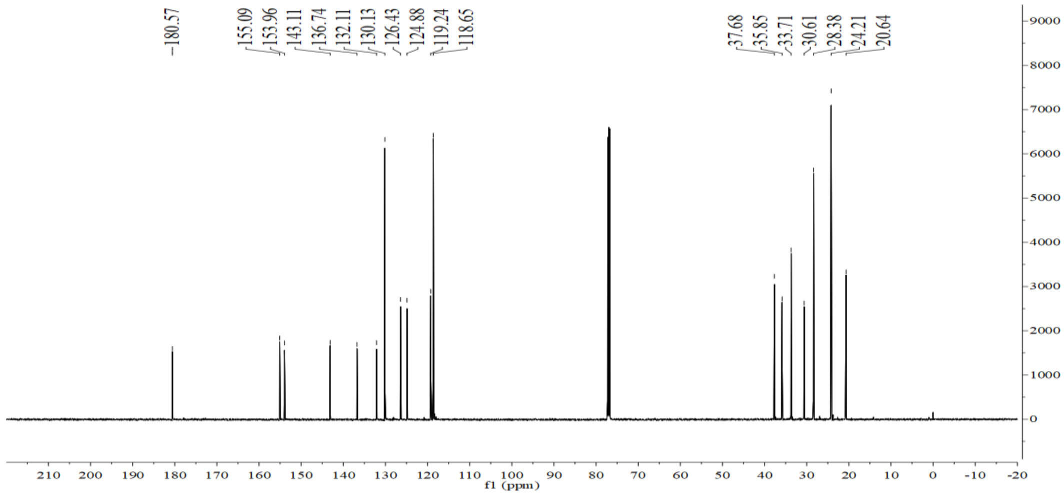

Figure S131. ^{13}C-NMR spectrum of **7j**.

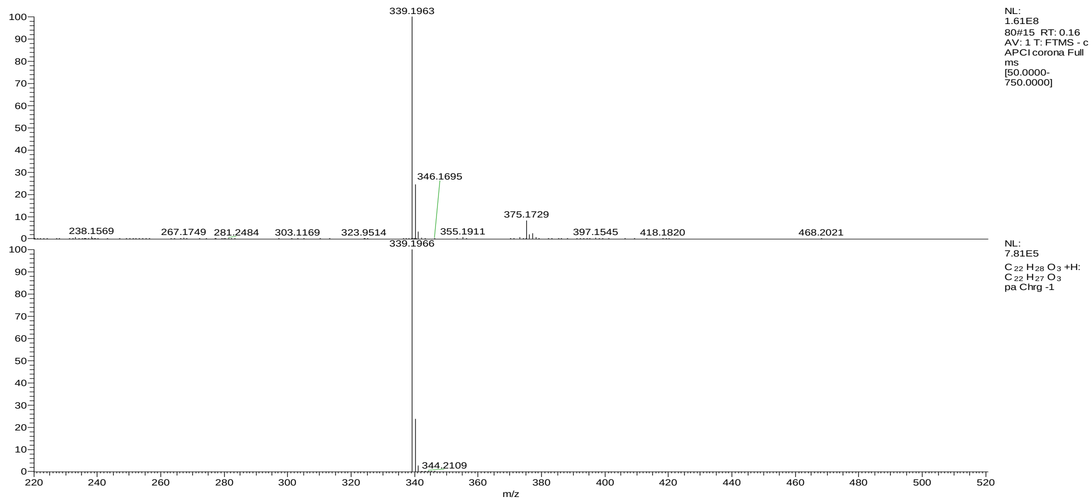

Figure S132. ESI-MS spectrum of **7j**.

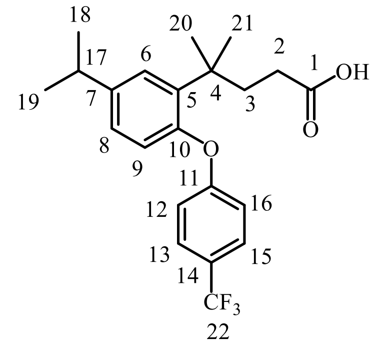

7k, white solid; yield 98.3%; m. p. 134.3 – 136.4 °C; FT-IR (KBr, cm^{-1}): 2966, 2876 (C-H), 1709 (C=O), 1490, 1413, 1322, 1215, 1202, 1215, 1134, 1099, 1064 (Ar-C=C); ^{1}H NMR (600 MHz, $CDCl_3$) δ 7.54 (d, J = 8.6 Hz, 2H, C_{13}-H, C_{15}-H), 7.17 (s, 1H, C_6-H), 7.03 (dd, J = 21.0, 9.4 Hz, 3H, C_8-H, C_{12}-H, C_{16}-H), 6.78 (d, J = 8.3 Hz, 1H, C_9-H), 2.94 – 2.86 (m, 1H, C_{17}-H), 2.17 – 2.07 (m, 4H, C_2-H, C_3-H), 1.37 (s, 6H, C_{20}-H, C_{21}-H), 1.25 (d, J = 6.9 Hz, 6H, C_{18}-H, C_{19}-H); ^{13}C NMR (151 MHz, $CDCl_3$) δ 180.27, 160.57, 152.03, 144.88, 137.84, 127.40 – 126.82 (m), 125.31, 120.96, 117.68, 37.69, 36.00, 33.83, 30.43, 28.46, 24.15; ESI-MS m/z: 393.1682 [M + H^+].

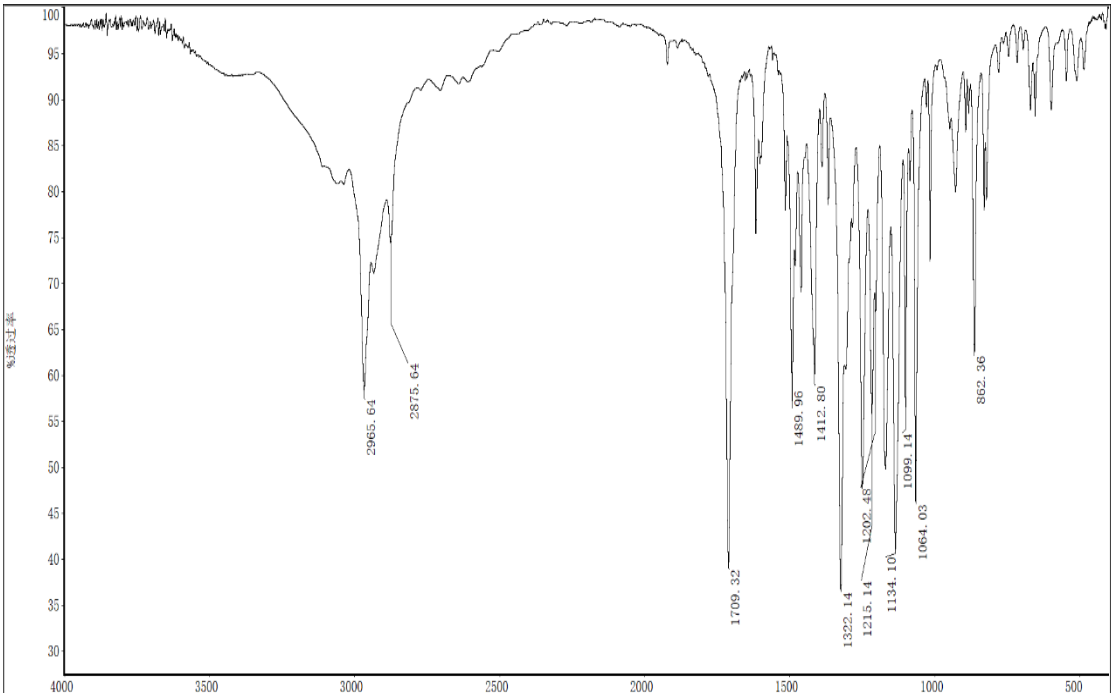

Figure S133. FT-IR spectrum of **7k**.

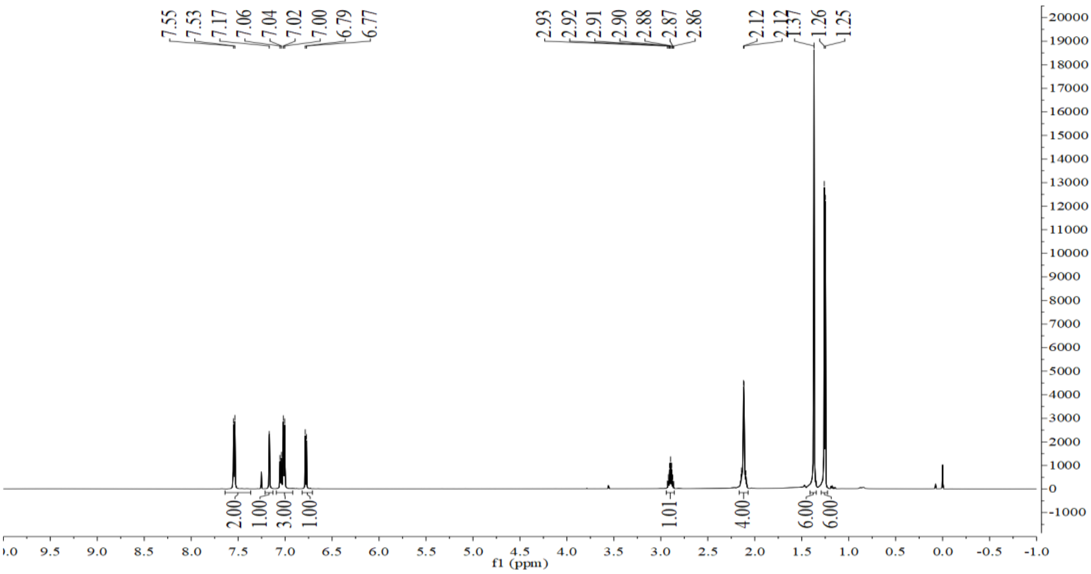

Figure S134. ^{1}H-NMR spectrum of **7k**.

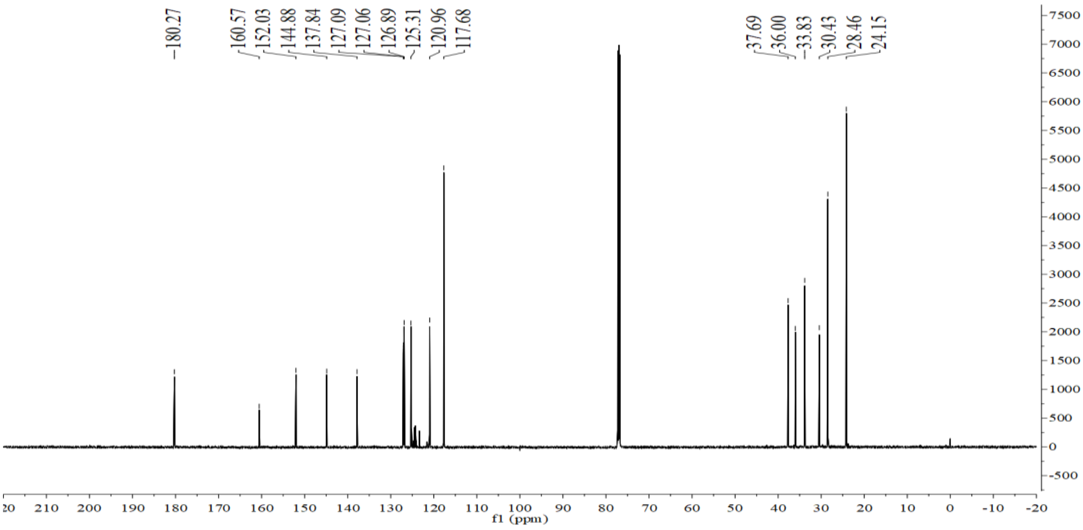

Figure S135. ^{13}C-NMR spectrum of **7k**.

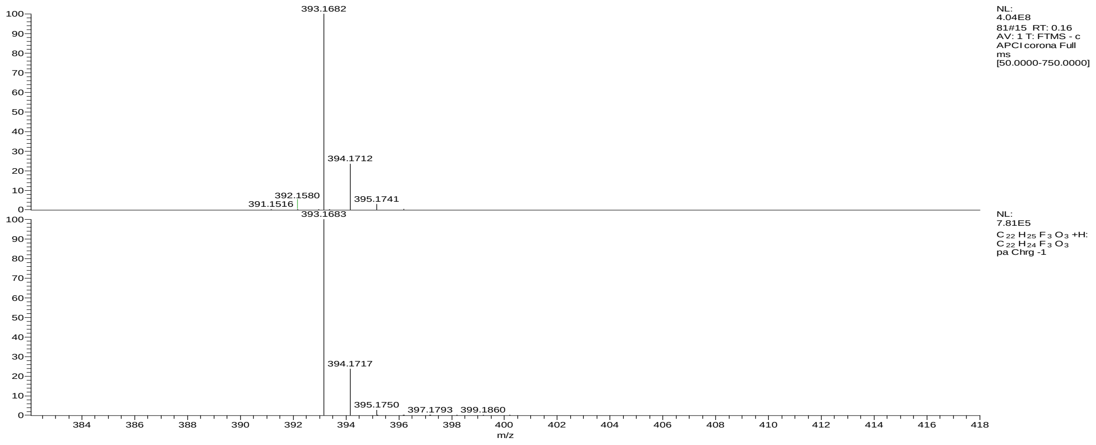

Figure S136. ESI-MS spectrum of **7k**.

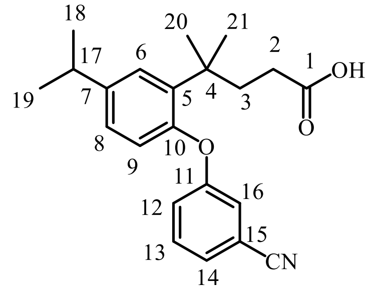

7l , white solid; yield 97.7%; m. p. 115.7 – 117.5 °C; FT-IR (KBr, cm^{-1}): 2965, 2933, 2867 (C-H), 2225 (C≡N), 1709 (C=O), 1586, 1482, 1428, 1412, 1320, 1252, 1225, 1205 (Ar-C=C); ^1H NMR (600 MHz, $CDCl_3$) δ 7.39 (t, J = 7.9 Hz, 1H, C_{14}-H), 7.32 (d, J = 7.3 Hz, 1H, C_{13}-H), 7.20 (dd, J = 16.6, 9.8 Hz, 3H, C_6-H, C_{12}-H, C_{16}-H), 7.05 (d, J = 8.2 Hz, 1H, C_8-H), 6.74 (d, J = 8.2 Hz, 1H, C_9-H), 2.94 – 2.84 (m, 1H, C_{17}-H), 2.12 (s, 4H, C_2-H, C_3-H), 1.37 (s, 6H, C_{20}-H, C_{21}-H), 1.26 (d, J = 6.9 Hz, 6H, C_{18}-H, C_{19}-H); ^{13}C NMR (151 MHz, $CDCl_3$) δ 180.13, 158.20, 151.89, 145.08, 137.82, 130.64, 127.01, 126.01, 125.46, 122.70, 120.88, 120.65, 118.35, 113.48, 37.68, 36.10, 33.82, 30.46, 28.43, 24.14; ESI-MS m/z: 350.1759 [M + H^+].

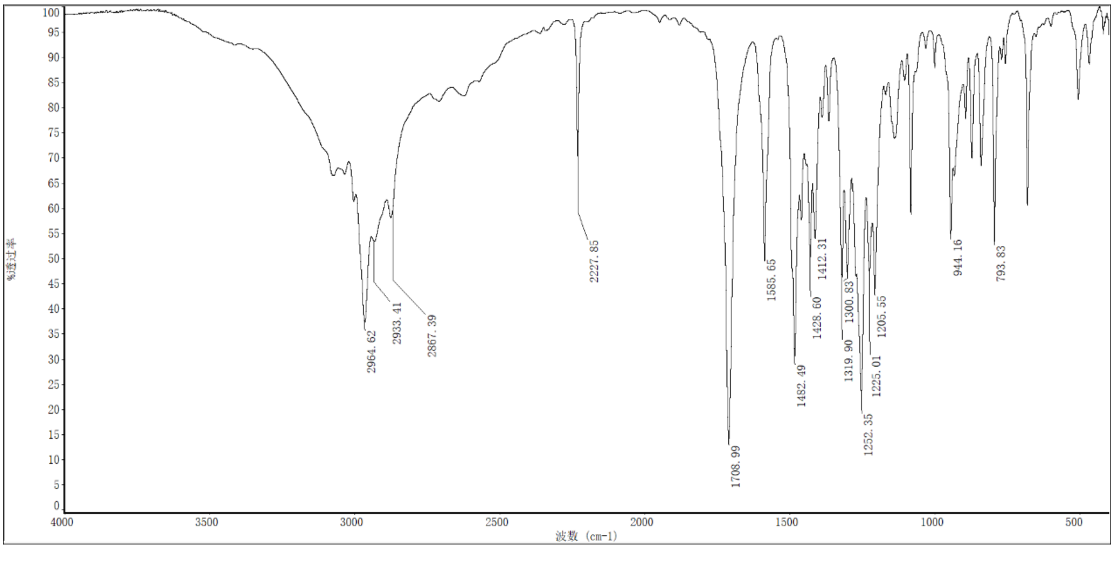

Figure S137. FT-IR spectrum of **7l**.

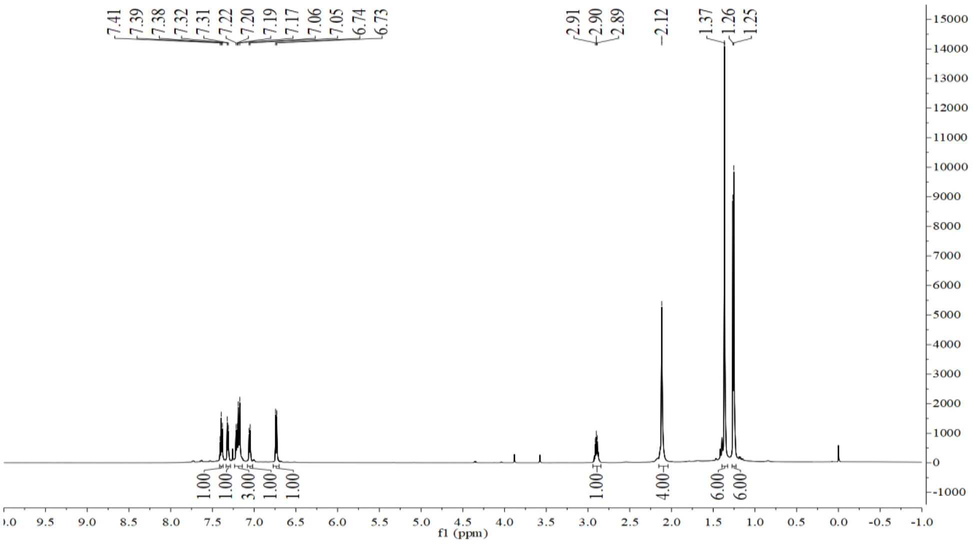

Figure S138. ^{1}H-NMR spectrum of **7l**.

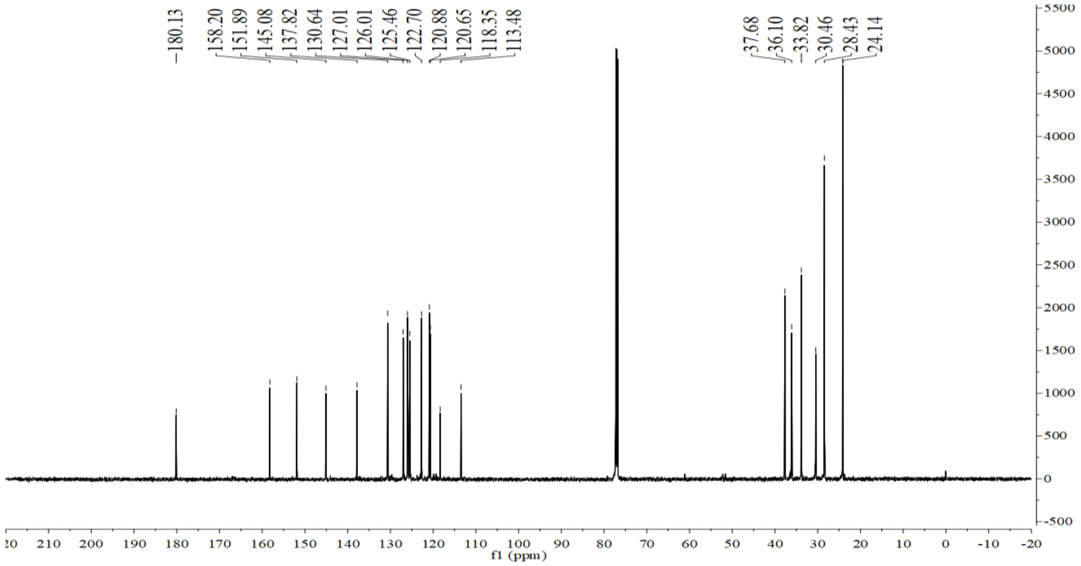

Figure S139. ^{13}C-NMR spectrum of **7l**.

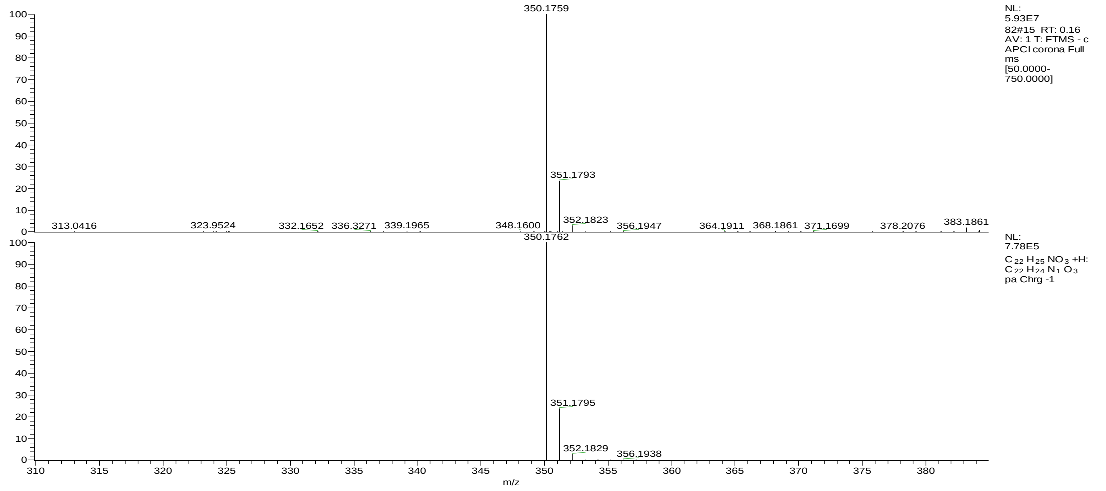

Figure S140. ESI-MS spectrum of **7l**.

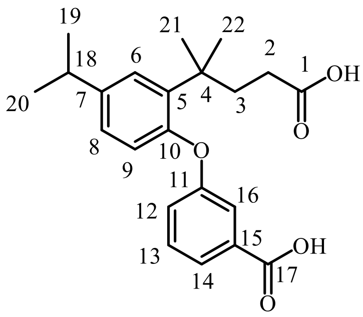

7m, white solid; yield 95.8%; m. p. 152.5 – 155.7 °C; FT-IR (KBr, cm^{-1}): 2965, 2869 (C-H), 1716 (C=O), 1589, 1485, 1446, 1414, 1300, 1281, 1226 (Ar-C=C); ^{1}H NMR (600 MHz, $CDCl_3$) δ 7.75 (d, *J* = 7.7 Hz, 1H, C_{14}-H), 7.46 (d, *J* = 37.9 Hz, 1H, C_{16}-H), 7.42 (t, *J* = 7.9 Hz, 1H, C_{13}-H), 7.31 (d, *J* = 9.9 Hz, 1H, C_8-H), 7.15 (d, *J* = 1.9 Hz, 1H, C_6-H), 7.02 (d, *J* = 10.3 Hz, 1H, C_{12}-H), 6.71 (d, *J* = 8.2 Hz, 1H, C_9-H), 2.89 (dt, *J* = 13.8, 6.9 Hz, 1H, C_{18}-H), 2.14 (d, *J* = 7.0 Hz, 2H, C_2-H), 2.10 (d, *J* = 9.7 Hz, 2H, C_3-H), 1.41 (d, *J* = 25.1 Hz, 6H, C_{21}-H, C_{22}-H), 1.26 (d, *J* = 6.9 Hz, 6H, C_{19}-H, C_{20}-H); ^{13}C NMR (151 MHz, $CDCl_3$) δ 180.93, 171.77, 157.66, 152.87, 144.21, 137.09, 131.11, 129.81, 127.04, 125.41, 124.43, 120.01, 119.04, 37.80, 35.47, 33.76, 30.46, 28.51, 24.16; ESI-MS m/z: 369.1705 [M + H^+].

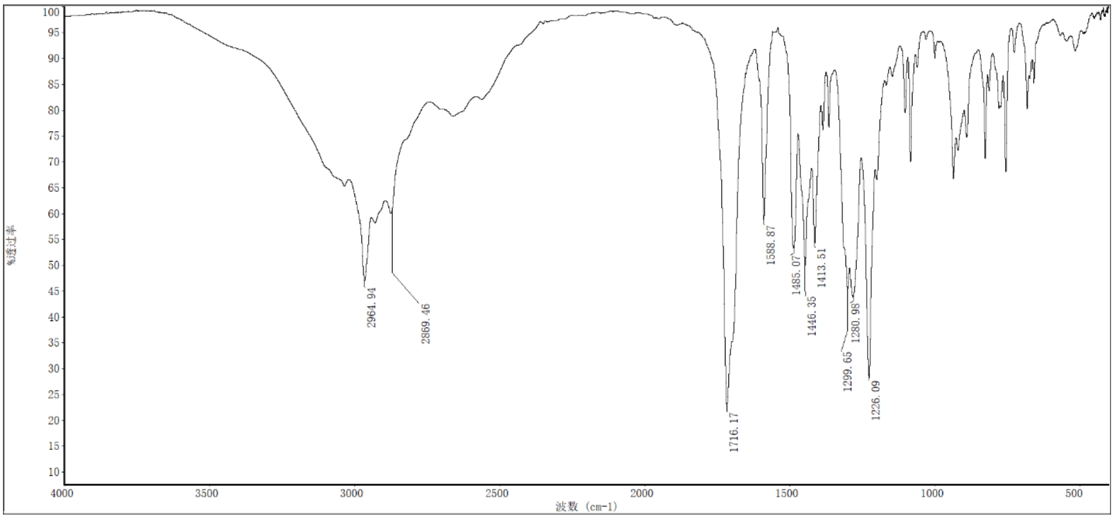

Figure S141. FT-IR spectrum of **7m**.

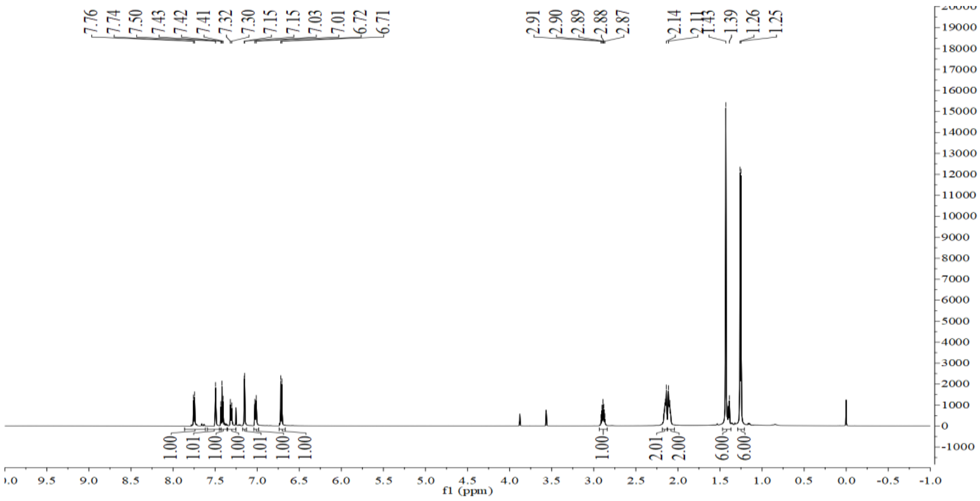

Figure S142. ^{1}H-NMR spectrum of **7m**.

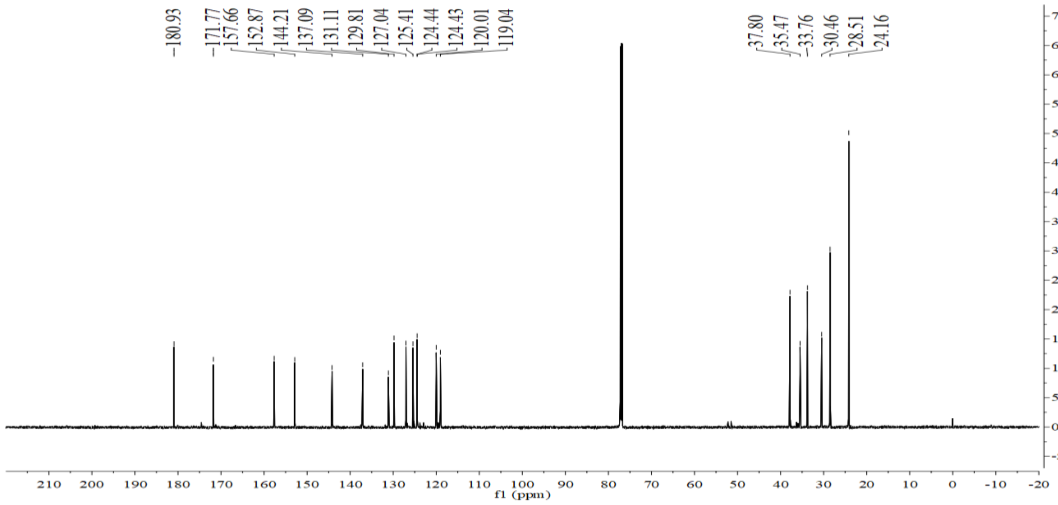

Figure S143. ^{13}C-NMR spectrum of **7m**.

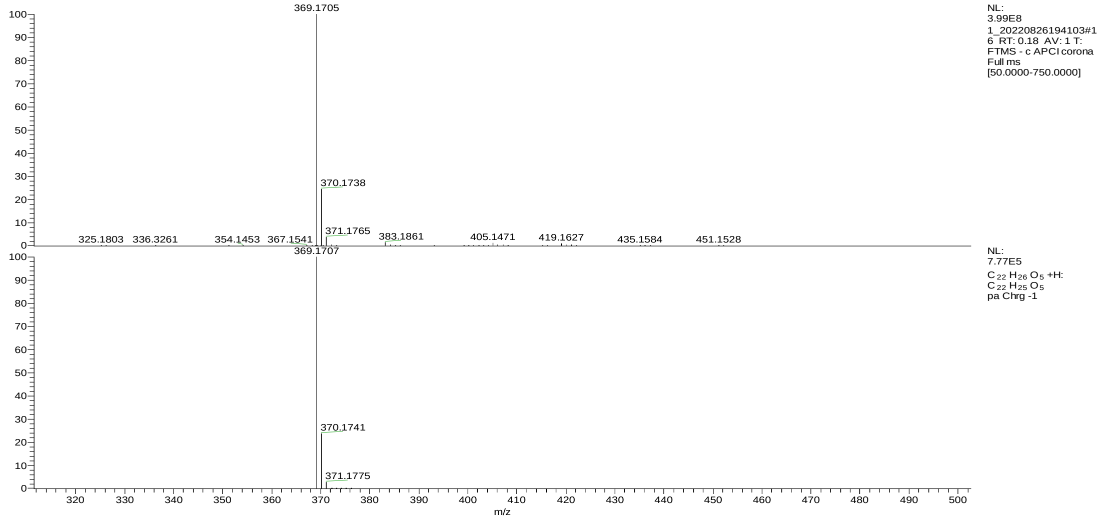

Figure S144. ESI-MS spectrum of **7m**.

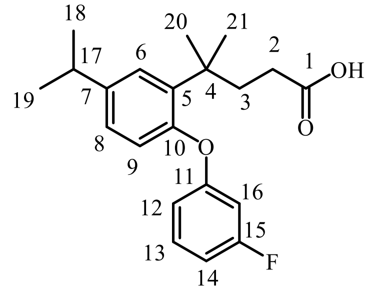

7n, white solid; yield 97.4%; m. p. 112.7– 113.9 °C; FT-IR (KBr, cm^{-1}): 2962, 2929, 2876 (C-H), 1704 (C=O), 1597, 1482, 1457, 1413, 1305, 1211, 1119 (Ar-C=C); ^{1}H NMR (600 MHz, $CDCl_3$) δ 7.25 – 7.21 (m, 1H, C_{13}-H), 7.14 (d, J = 2.2 Hz, 1H, C_6-H), 7.03 (d, J = 6.1 Hz, 1H, C_8-H), 6.78 (d, J = 8.3 Hz, 1H, C_9-H), 6.73 (td, J = 7.9, 2.3 Hz, 2H, C_{14}-H, C_{16}-H), 6.66 (dt, J = 10.4, 2.3 Hz, 1H, C_{12}-H), 2.88 (dt, J = 13.8, 6.9 Hz, 1H, C_{17}-H), 2.12 (dt, J = 18.3, 9.1 Hz, 4H, C_2-H, C_3-H), 1.38 (s, 6H, C_{20}-H, C_{21}-H), 1.25 (d, J = 6.9 Hz, 6H, C_{18}-H, C_{19}-H); ^{13}C NMR (151 MHz, $CDCl_3$) δ 180.22, 164.38, 162.75, 152.53, 144.37, 137.50, 130.39 (d, J = 9.8 Hz), 126.71, 125.17, 120.57, 113.71 (d, J = 2.9 Hz), 109.34 , 109.20, 105.77, 105.61, 37.68, 36.00, 33.79, 30.48, 28.43, 24.17; ESI-MS m/z: 343.1712 [M + H^{+}].

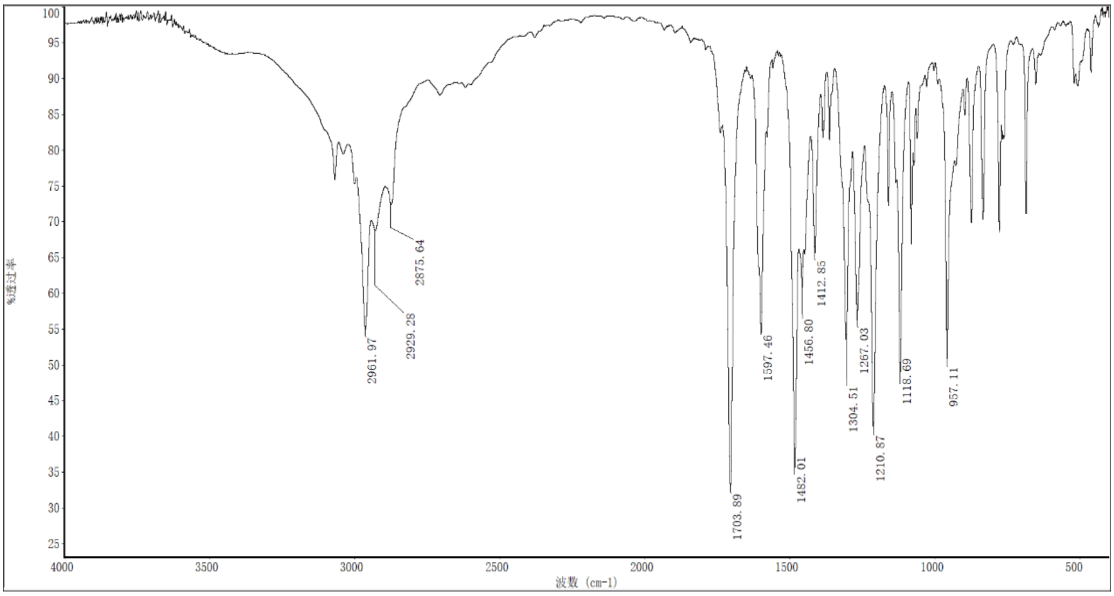

Figure S145. FT-IR spectrum of **7n**.

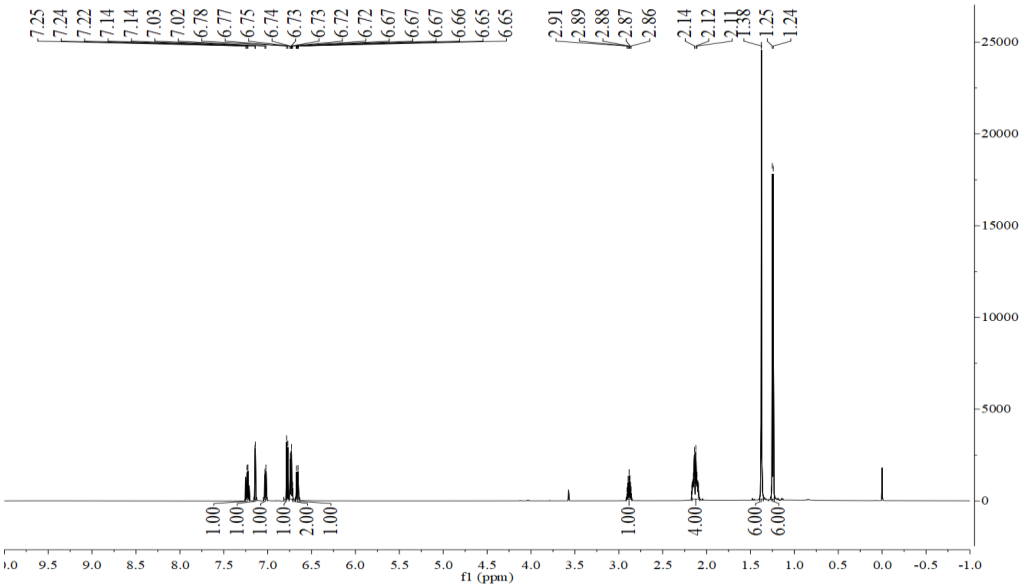

Figure S146. ^{1}H-NMR spectrum of **7n**.

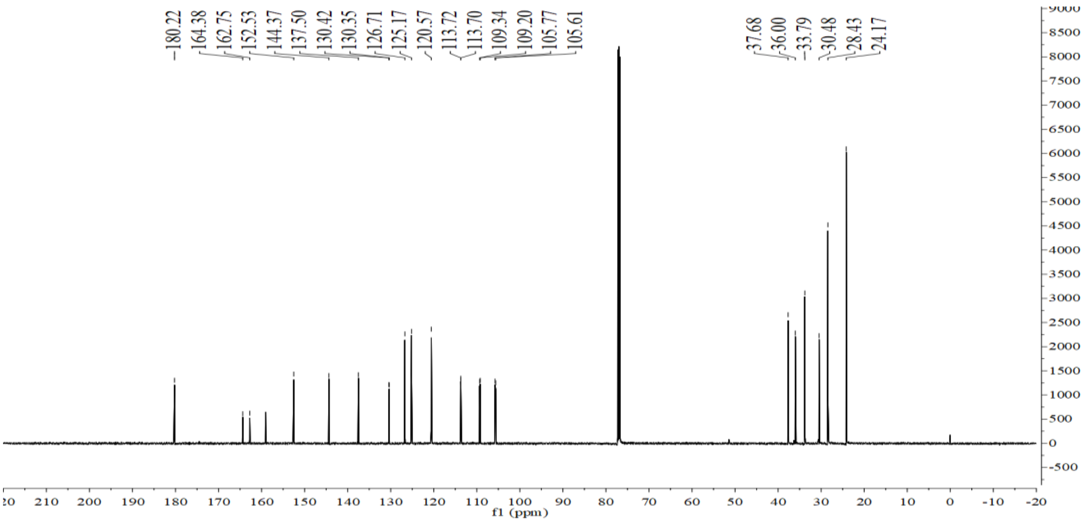

Figure S147. ^{13}C-NMR spectrum of **7n**.

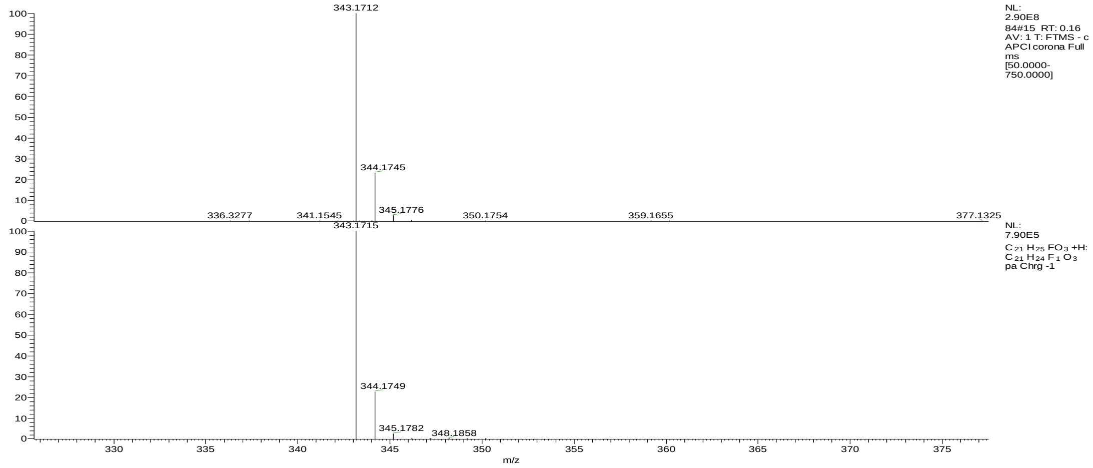

Figure S148. ESI-MS spectrum of **7n**.

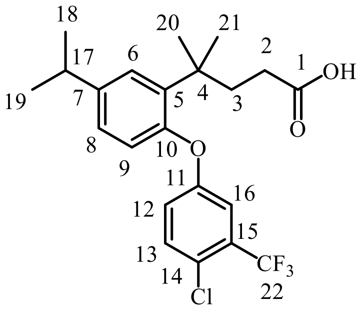

7o, white solid; yield 96.7%; m. p. 113.0– 114.1 °C; FT-IR (KBr, cm^{-1}): 2962, 2927 (C-H), 1711 (C=O), 1477, 1427, 1328, 1164, 1126, 1108, 1082 (Ar-C=C); ^1H NMR (600 MHz, $CDCl_3$) δ 7.39 (d, J = 8.8 Hz, 1H, C_{13}-H), 7.31 (d, J = 2.9 Hz, 1H, C_{16}-H), 7.16 (d, J = 2.1 Hz, 1H, C_6-H), 7.05 – 7.01 (m, 2H, C_8-H, C_{12}-H), 6.72 (d, J = 8.3 Hz, 1H, C_9-H), 2.89 (dt, J = 13.8, 6.9 Hz, 1H, C_{17}-H), 2.15 – 2.09 (m, 4H, C_2-H, C_3-H), 1.37 (s, 6H, C_{20}-H, C_{21}-H), 1.25 (d, J = 6.9 Hz, 6H, C_{18}-H, C_{19}-H); ^{13}C NMR (151 MHz, $CDCl_3$) δ 180.03, 156.26, 152.19, 144.89, 137.57, 132.57, 126.97, 125.41, 121.95, 120.18, 117.46 (d, J = 5.4 Hz), 37.67, 35.99, 33.77, 30.39, 28.38, 24.10; ESI-MS m/z: 427.1289 [M + H^+].

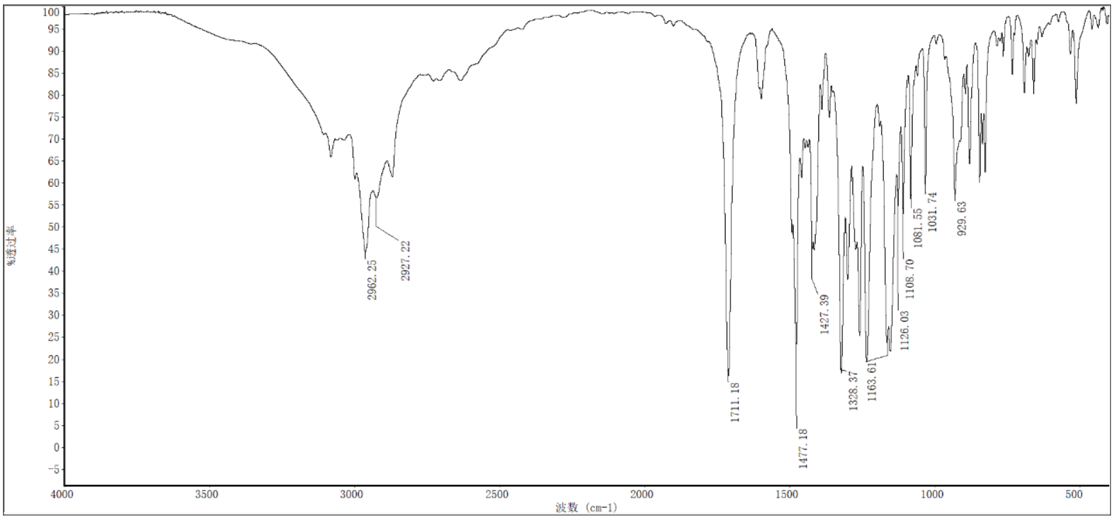

Figure S149. FT-IR spectrum of **7o**.

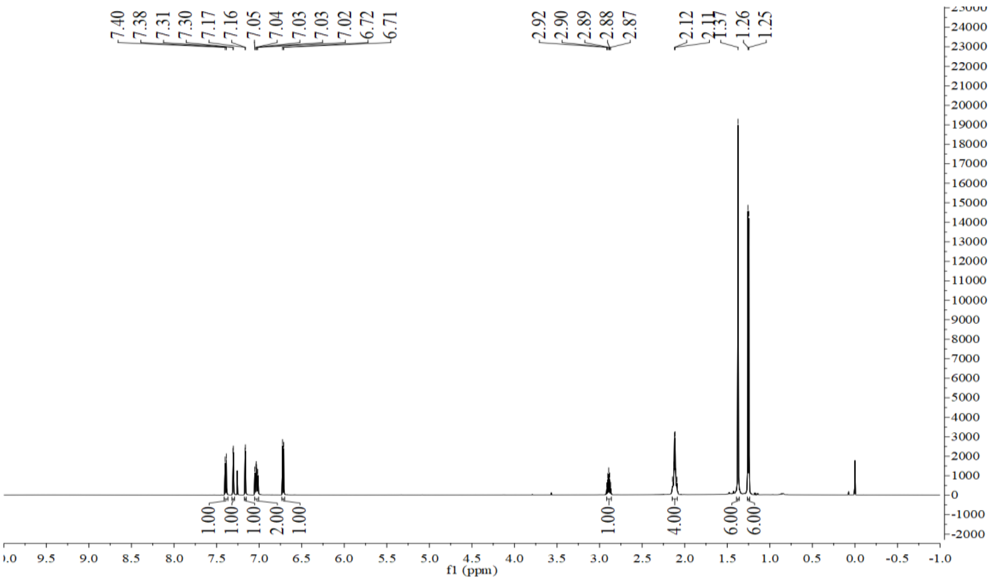

Figure S150. ^{1}H-NMR spectrum of **7o**.

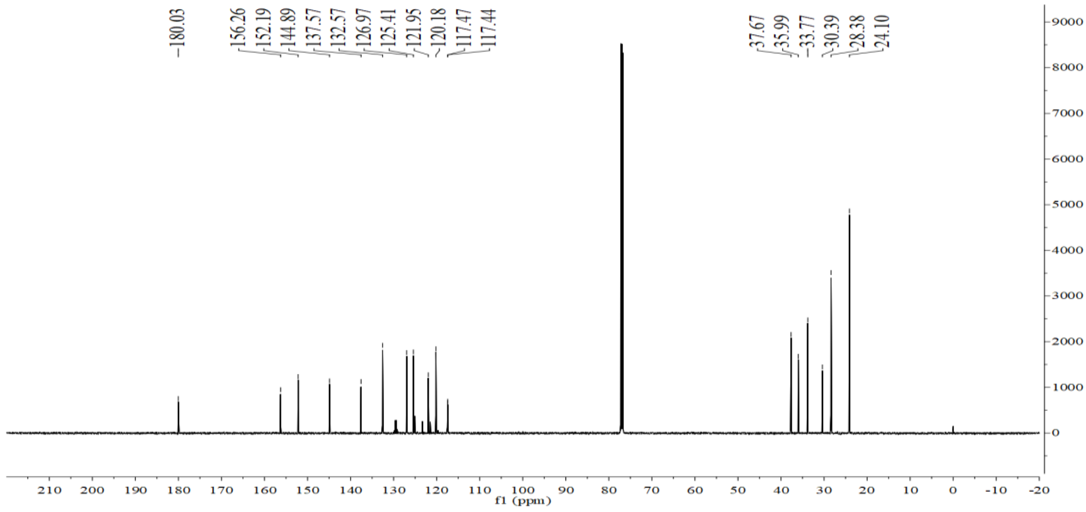

Figure S151. ^{13}C-NMR spectrum of **7o**.

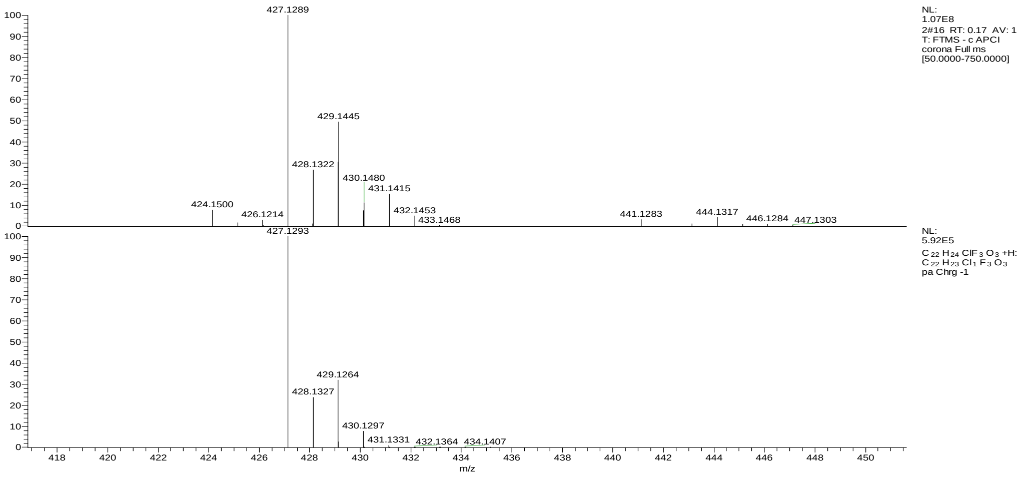

Figure S152. ESI-MS spectrum of **7o**.

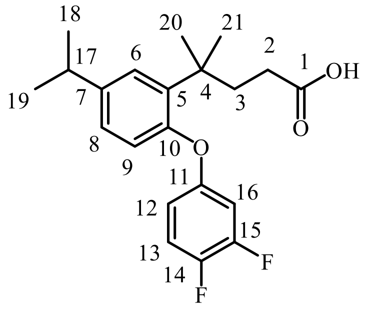

7p, white solid; yield 98.8%; m. p. 106.4– 108.2 °C; FT-IR (KBr, cm^{-1}): 3061 (Ar-H), 2962, 2929, 2874 (C-H), 1702 (C=O), 1513, 1489, 1456, 1412, 1303, 1249, 1213, 1145, 1102 (Ar-C=C); ^{1}H NMR (600 MHz, $CDCl_3$) δ 7.14 (d, J = 2.2 Hz, 1H, C_{13}-H), 7.10 – 7.05 (m, 1H, C_6-H), 7.02 (d, J = 8.3 Hz, 1H, C_8-H), 6.79 (dd, J = 11.5, 6.7 Hz, 1H, C_9-H), 6.72 (d, J = 8.3 Hz, 1H, C_{16}-H), 6.69 – 6.65 (m, 1H, C_{12}-H), 2.87 (dd, J = 13.8, 6.9 Hz, 1H, C_{17}-H), 2.14 (d, J = 12.1 Hz, 2H, C_2-H), 2.12 – 2.08 (m, 2H, C_3-H), 1.38 (s, 6H, C_{20}-H, C_{21}-H), 1.24 (d, J = 6.9 Hz, 6H, C_{18}-H, C_{19}-H; ^{13}C NMR (151 MHz, $CDCl_3$) δ 180.06, 152.89, 144.35, 137.27, 126.79, 125.23, 119.95, 117.44 (d, J = 19.6 Hz), 113.77 (dd, J = 5.8, 3.5 Hz), 107.87, 107.74, 37.69, 35.97, 33.77, 30.45, 28.39, 24.16; ESI-MS m/z: 361.1619 [M + H^+].

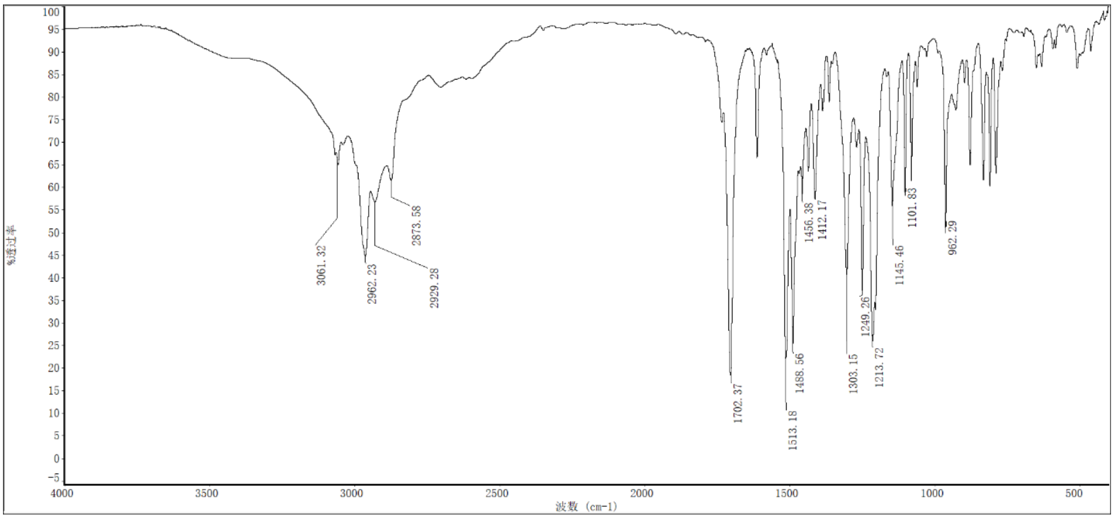

Figure S153. FT-IR spectrum of **7p**.

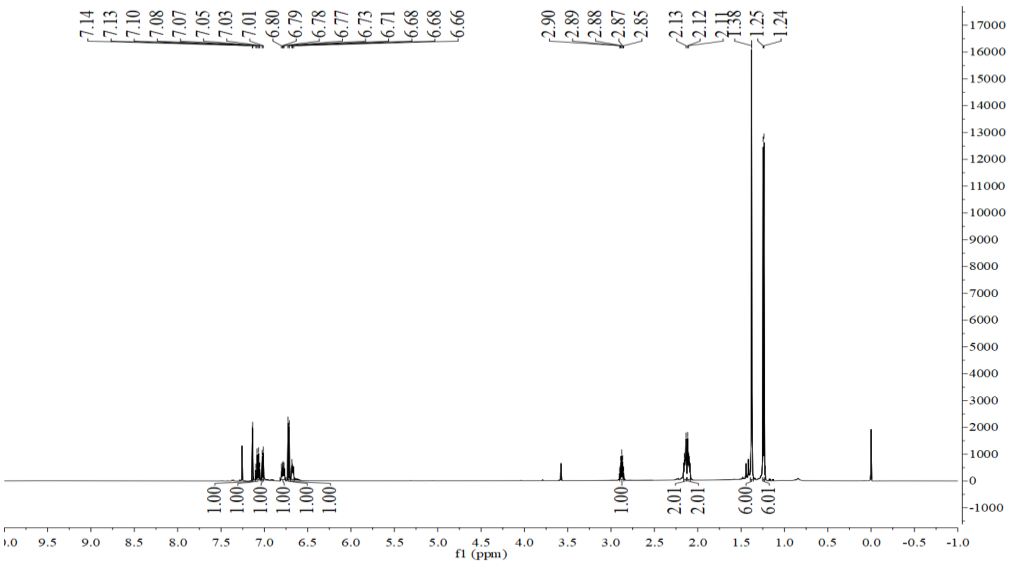

Figure S154. ^{1}H-NMR spectrum of **7p**.

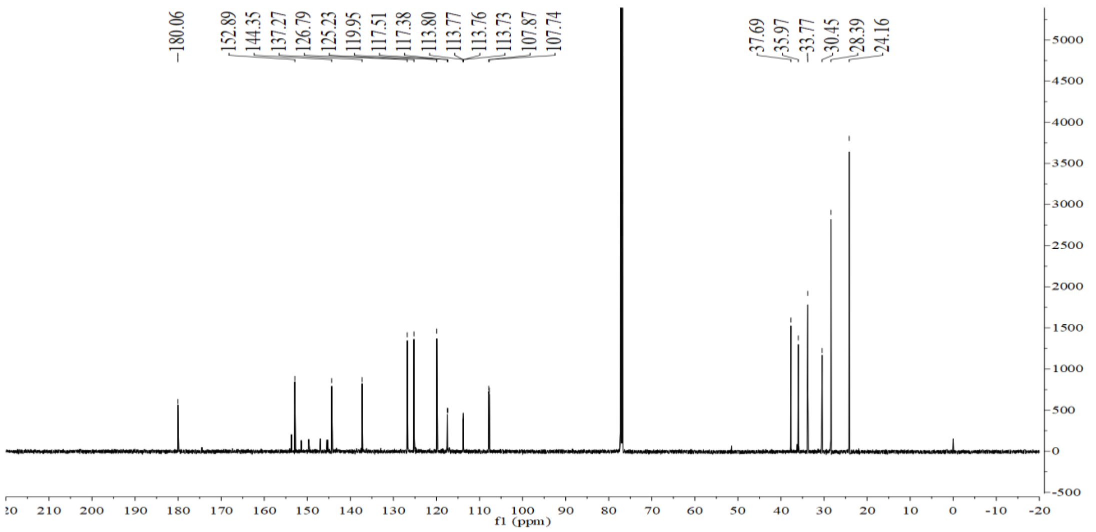

Figure S155. ^{13}C-NMR spectrum of **7p**.

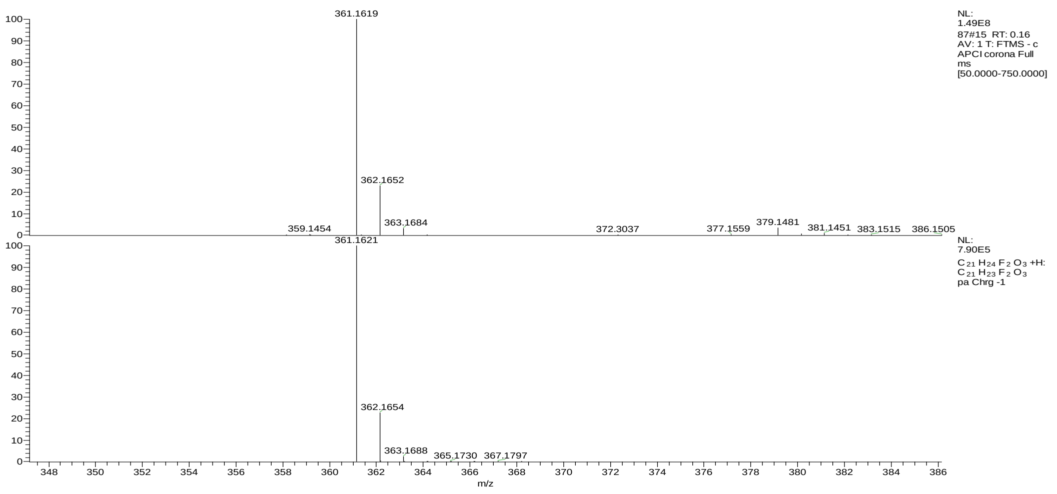

Figure S156. ESI-MS spectrum of **7p**.

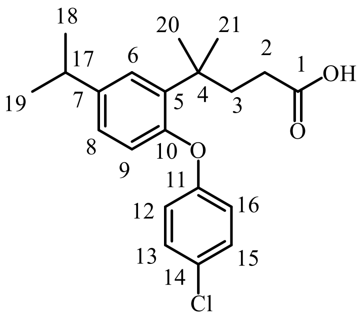

7q, white solid; yield 90.8%; m. p. 121.5– 123.8 °C; FT-IR (KBr, cm^{-1}): 2956, 2871 (C-H), 1709 (C=O), 1483, 1456, 1411, 1291, 1244, 1197, 1082 (Ar-C=C); ^{1}H NMR (600 MHz, $CDCl_3$) δ 7.25 (d, J = 9.1 Hz, 2H, C_{13}-H, C_{15}-H), 7.13 (d, J = 2.2 Hz, 1H, C_6-H), 7.00 (d, J = 6.2 Hz, 1H, C_8-H), 6.89 (d, J = 8.9 Hz, 2H, C_{12}-H, C_{16}-H), 6.71 (d, J = 8.3 Hz, 1H, C_9-H), 2.87 (dt, J = 13.8, 6.9 Hz, 1H, C_{17}-H), 2.20 – 2.14 (m, 2H, C_2-H), 2.13 – 2.08 (m, 2H, C_3-H), 1.38 (s, 6H, C_{20}-H, C_{21}-H), 1.24 (d, J = 6.9 Hz, 6H, C_{18}-H, C_{19}-H); ^{13}C NMR (151 MHz, $CDCl_3$) δ 180.00, 156.22, 153.05, 144.04, 137.25 , 129.60 (d, J = 4.9 Hz), 127.53, 126.68, 125.12, 119.93, 119.59, 37.68, 35.93, 33.76, 30.46, 28.40, 24.17; ESI-MS m/z: 359.1418 [M + H^{+}].

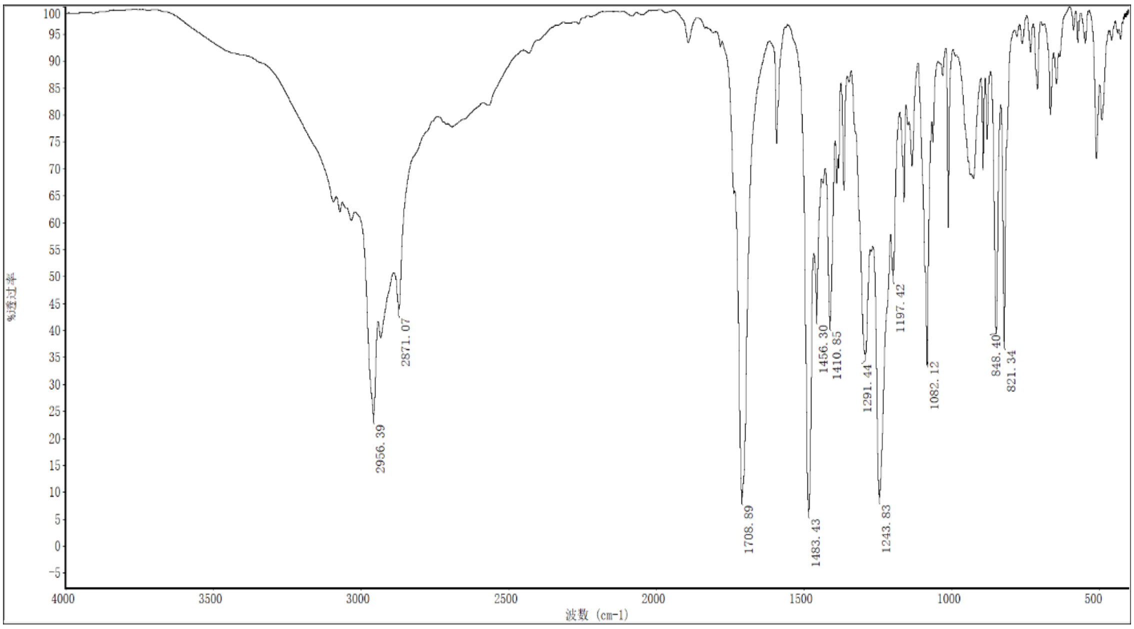

Figure S157. FT-IR spectrum of **7q**.

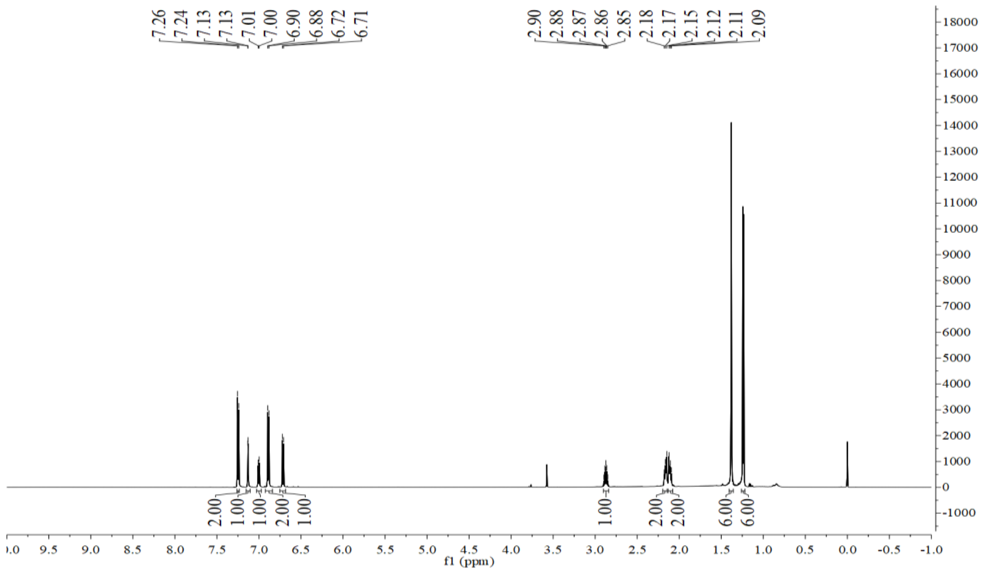

Figure S158. ^{1}H-NMR spectrum of **7q**.

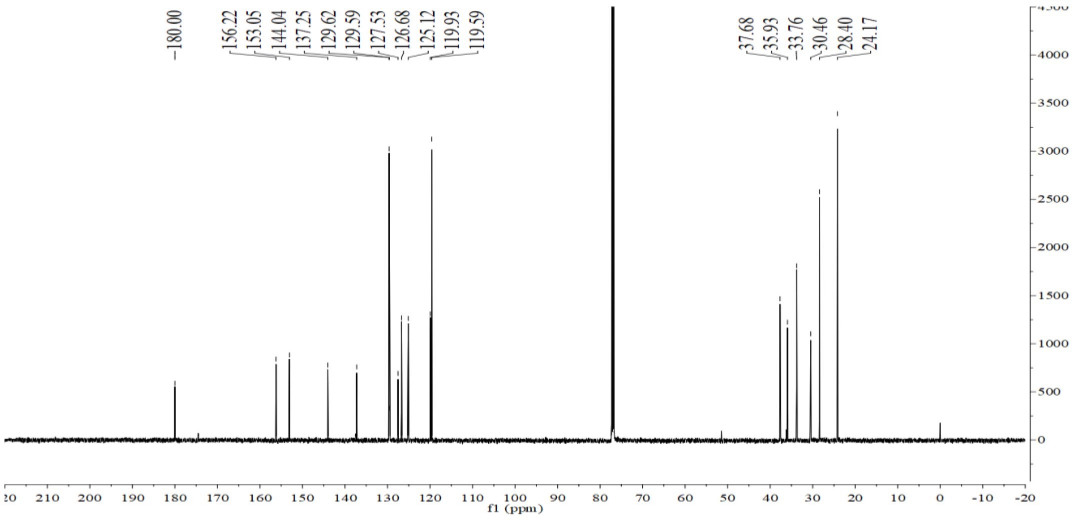

Figure S159. ^{13}C-NMR spectrum of **7q**.

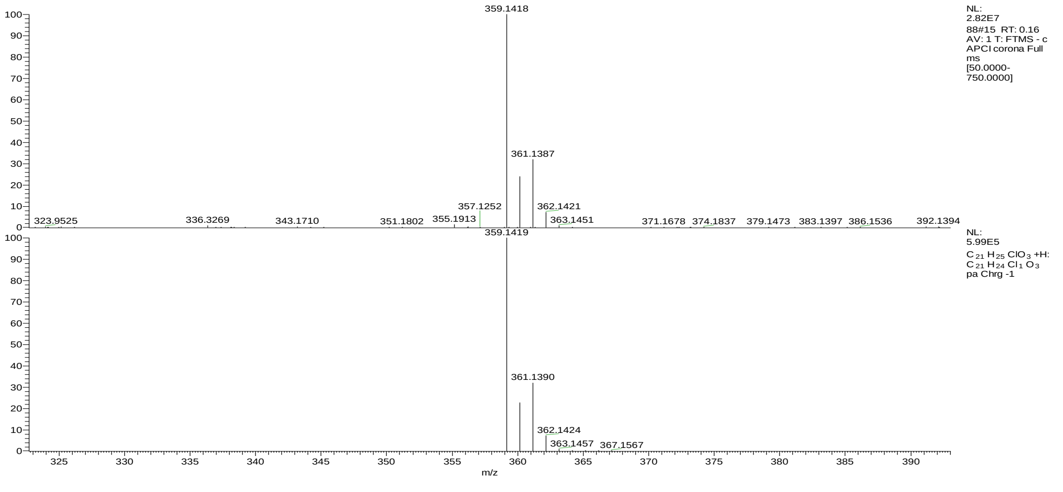

Figure S160. ESI-MS spectrum of **7q**.

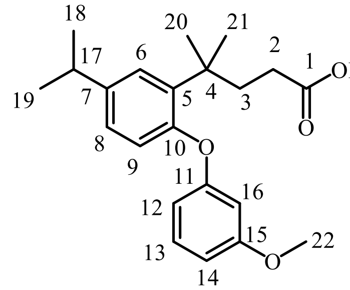

7r, white solid; yield 94.2%; m. p. 125.6– 126.9 °C; FT-IR (KBr, cm^{-1}): 2959, 2874 (C-H), 1708 (C=O), 1603, 1486, 1454, 1412, 1386, 1282, 1214, 1162, 1141, 1083, 1043 (Ar-C=C); ^{1}H NMR (600 MHz, $CDCl_3$) δ 7.17 (t, J = 8.1 Hz, 1H, C_{13}-H), 7.13 (s, 1H, C_6-H), 6.99 (d, J = 8.3 Hz, 1H, C_8-H), 6.77 (d, J = 8.3 Hz, 1H, C_9-H), 6.59 (d, J = 8.7 Hz, 1H, C_{16}-H), 6.53 (d, J = 8.7 Hz, 2H, C_{12}-H, C_{14}-H), 3.75 (s, 3H, C_{22}-H), 2.90 – 2.84 (m, 1H, C_{17}-H), 2.22 – 2.17 (m, 2H, C_2-H), 2.12 (dd, J = 9.1, 7.3 Hz, 2H, C_3-H), 1.40 (s, 6H, C_{20}-H, C_{21}-H), 1.24 (d, J = 7.0 Hz, 6H, C_{18}-H, C_{19}-H); ^{13}C NMR (151 MHz, $CDCl_3$) δ 180.83, 160.92, 158.76, 153.18, 143.66, 137.11, 130.02, 126.51, 125.00, 120.14, 110.67, 108.30, 104.54, 60.45, 55.27, 38.10, 35.94, 33.95 – 33.56 , 30.64, 28.43, 24.13, 14.15; ESI-MS m/z: 355.1912 [M + H^+].

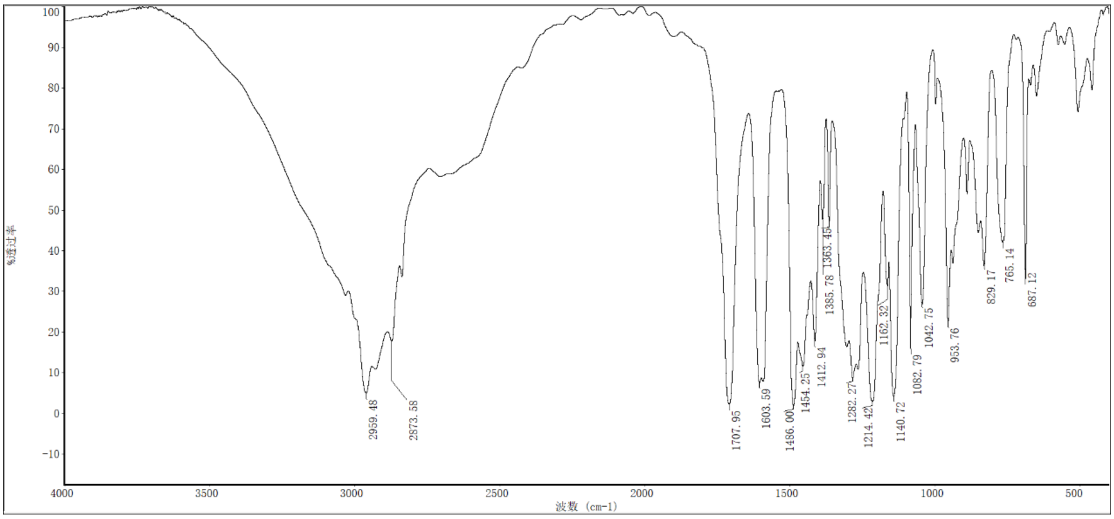

Figure S161. FT-IR spectrum of **7r**.

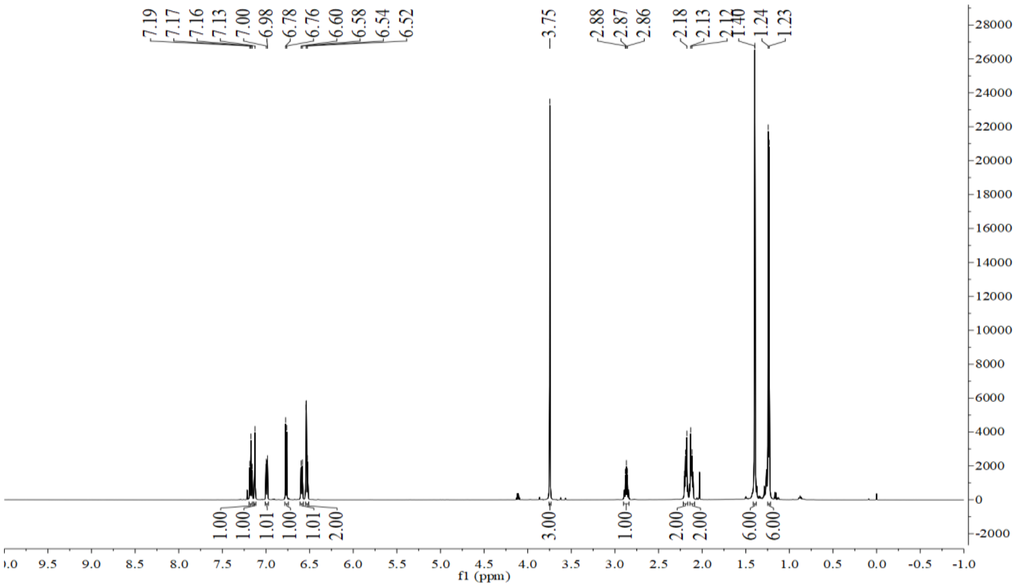

Figure S162. ^{1}H-NMR spectrum of **7r**.

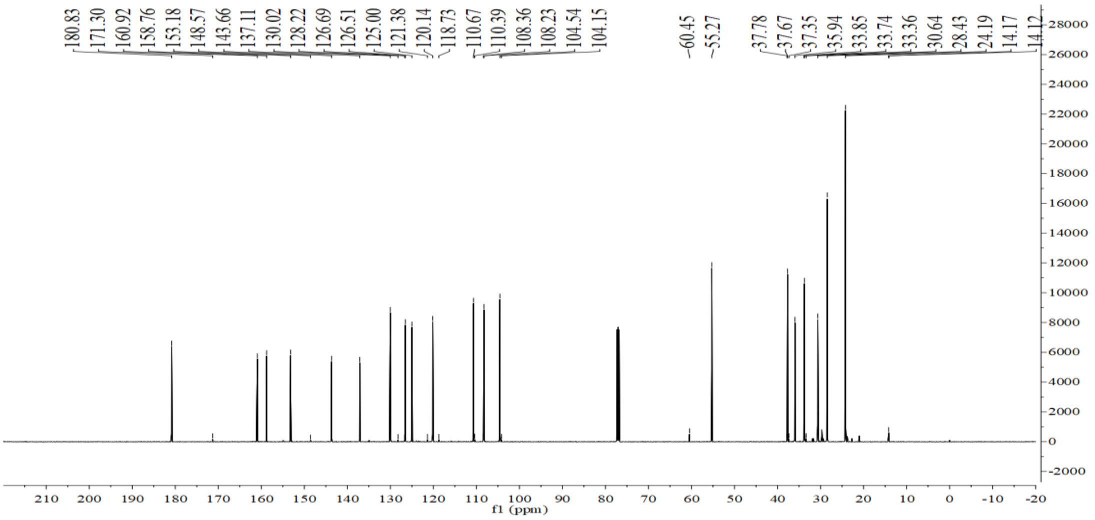

Figure S163. ^{13}C-NMR spectrum of **7r**.

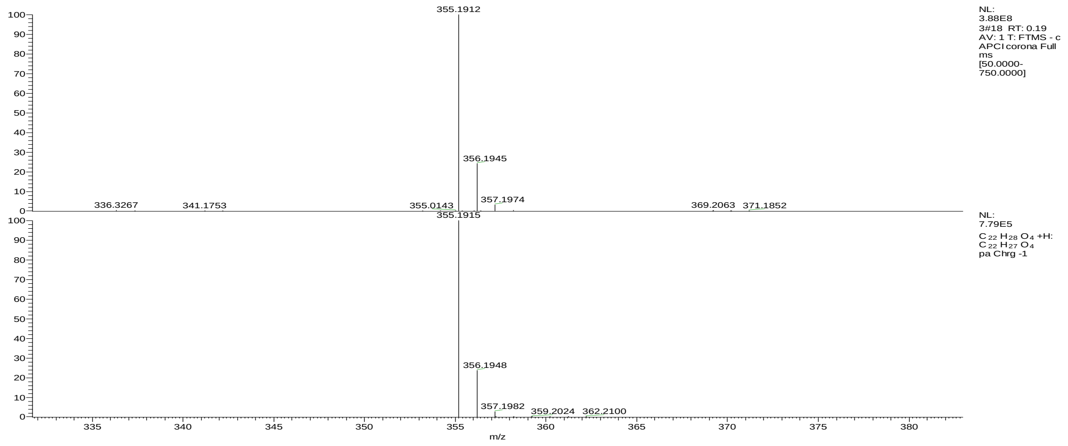

Figure S164. ESI-MS spectrum of **7r**.

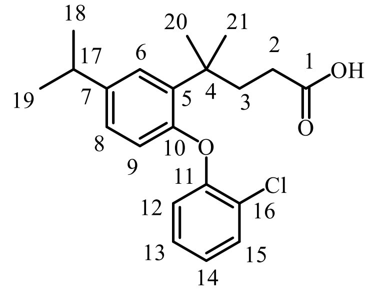

7s, white solid; yield 94.9%; m. p. 124.1– 125.8 °C; FT-IR (KBr, cm^{-1}): 3432 (Ar-H), 2959, 2925, 2870 (C-H), 1708 (C=O), 1475, 1446, 1412, 1263, 1238, 1200, 1082, 1059 (Ar-C=C); ^{1}H NMR (600 MHz, $CDCl_3$) δ 7.44 (d, J = 6.4 Hz, 1H, C_{15}-H), 7.18 – 7.12 (m, 2H, C_6-H, C_{13}-H), 7.02 (t, J = 7.7 Hz, 1H, C_{14}-H), 6.98 (d, J = 8.3 Hz, 1H, C_8-H), 6.91 (d, J = 9.6 Hz, 1H, C_{12}-H), 6.59 (d, J = 8.3 Hz, 1H, C_9-H), 2.87 (dt, J = 13.8, 6.9 Hz, 1H, C_{17}-H), 2.26 – 2.21 (m, 2H, C_2-H), 2.16 – 2.11 (m, 2H, C_3-H), 1.44 (s, 6H, C_{20}-H, C_{21}-H), 1.23 (d, J = 6.9 Hz, 6H, C_{18}-H, C_{19}-H); ^{13}C NMR (151 MHz, $CDCl_3$) δ 180.83, 160.92, 158.76, 153.18, 143.66, 137.11, 130.02, 126.51, 125.00, 120.14, 110.67, 108.23, 104.54, 55.27, 37.67, 35.94, 33.74, 30.64, 28.43, 24.19; ESI-MS m/z: 359.1418 [M + H^+].

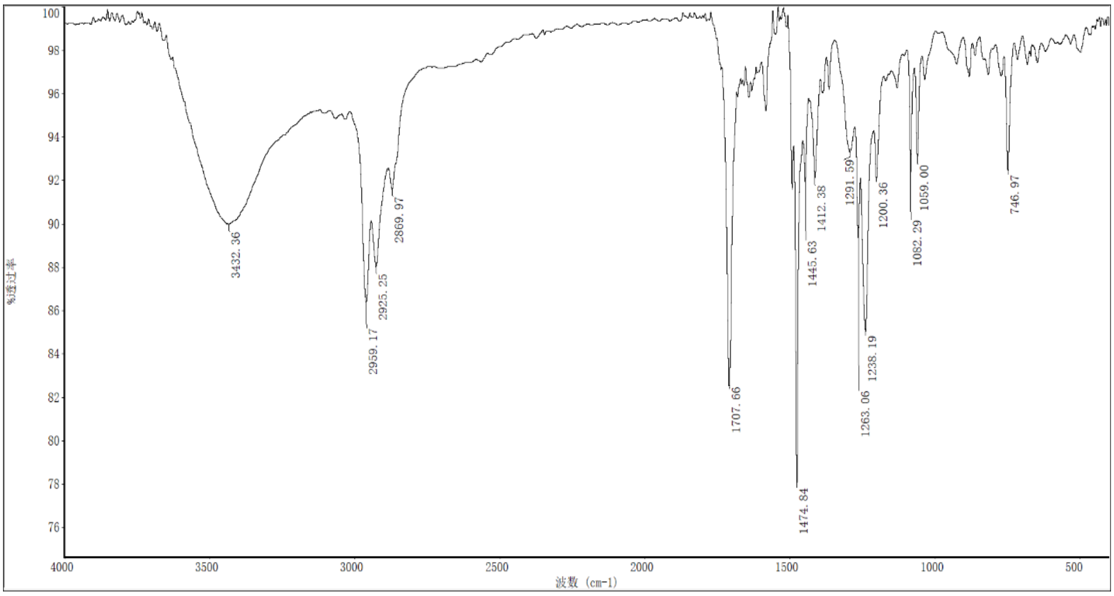

Figure S165. FT-IR spectrum of **7s**.

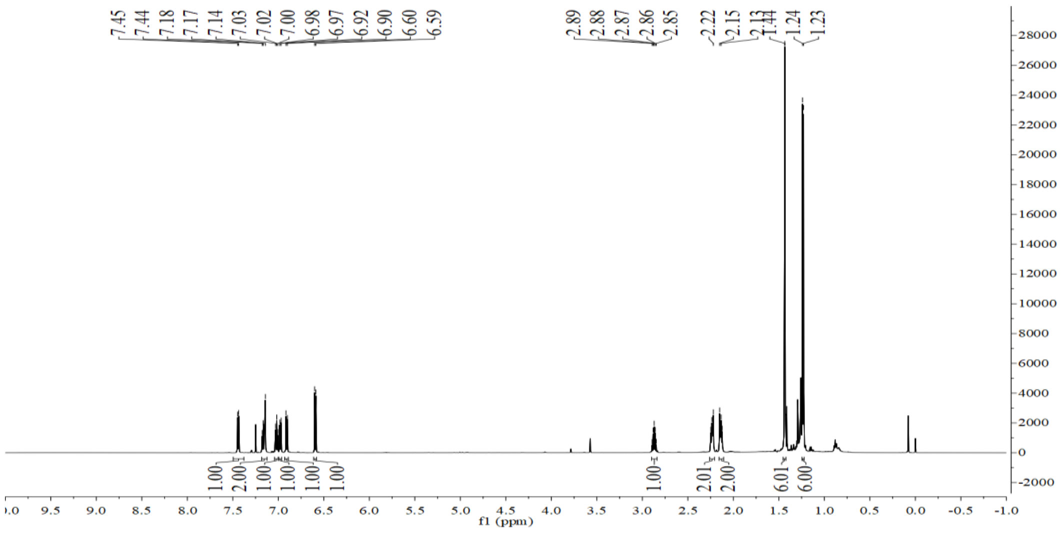

Figure S166. ^{1}H-NMR spectrum of **7s**.

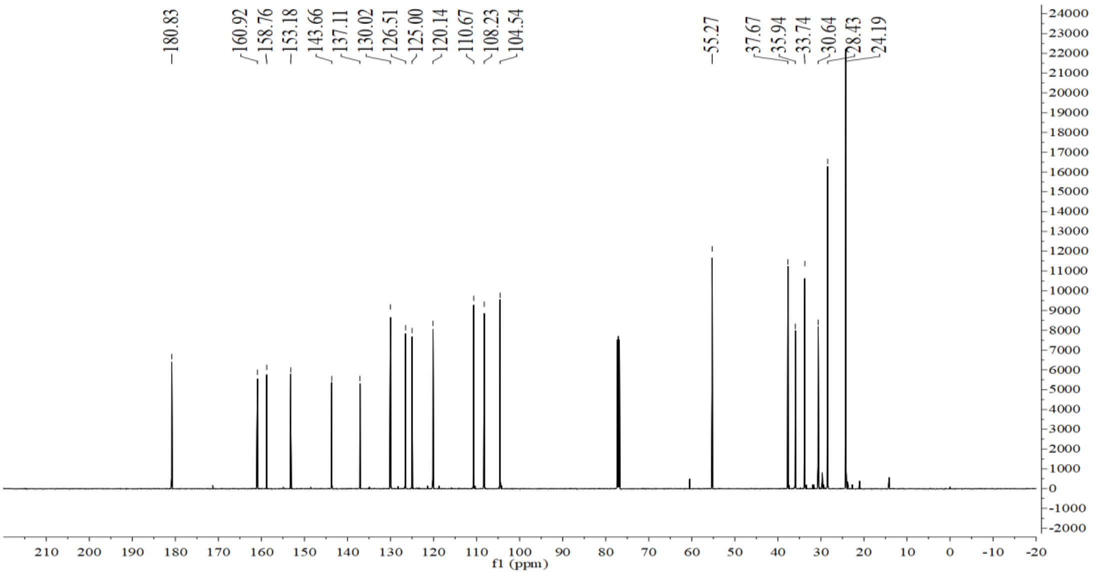

Figure S167. ^{13}C-NMR spectrum of **7s**.

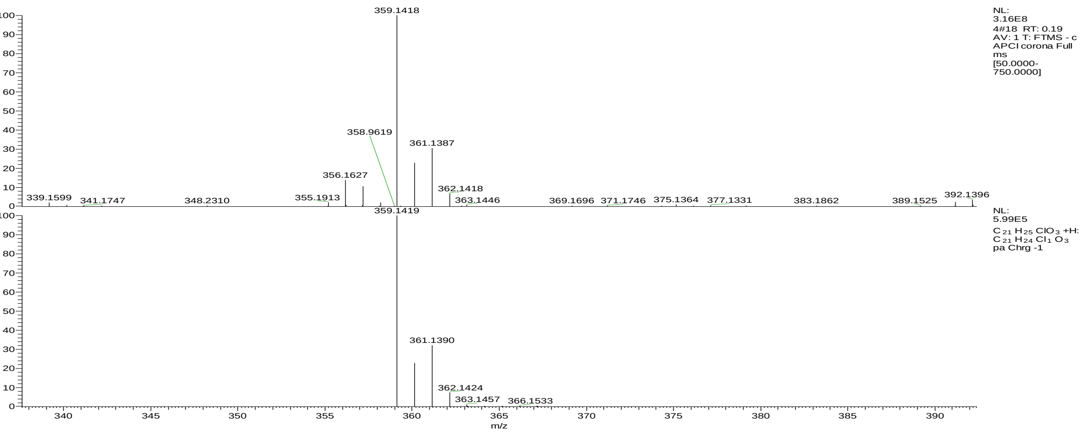

Figure S168. ESI-MS spectrum of **7s**.

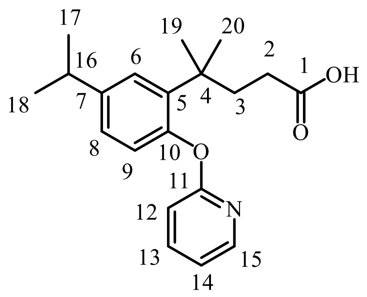

7t, white solid; yield 97.6%; m. p. 133.2– 134.9 °C; FT-IR (KBr, cm^{-1}): 2960, 2926, 2871 (C-H), 1710 (C=O), 1574, 1490, 1476, 1427, 1245, 1200, 1081 (Ar-C=C); ^1H NMR (600 MHz, $CDCl_3$) δ 8.20 (d, J = 6.4 Hz, 1H, C_{15}-H), 7.65 (t, J = 7.8 Hz, 1H, C_{13}-H), 7.16 (s, 1H, C_6-H), 7.06 (d, J = 10.3 Hz, 1H, C_8-H), 6.96 – 6.93 (m, 1H, C_9-H), 6.88 – 6.84 (m, 2H, C_{12}-H, C_{14}-H), 2.89 (dt, J = 13.8, 6.9 Hz, 1H, C_{16}-H), 2.11 (s, 4H, C_2-H, C_3-H), 1.37 (s, 6H, C_{19}-H, C_{20}-H), 1.25 (d, J = 7.0 Hz, 6H, C_{17}-H, C_{18}-H); ^{13}C NMR (151 MHz, $CDCl_3$) δ 179.64, 163.70, 150.62, 147.75, 144.80, 139.56, 137.90, 126.67, 125.07, 122.52, 118.27, 111.71, 37.63, 36.33, 33.83, 30.53, 28.58, 24.16; ESI-MS m/z: 326.1759 [M + H^+].

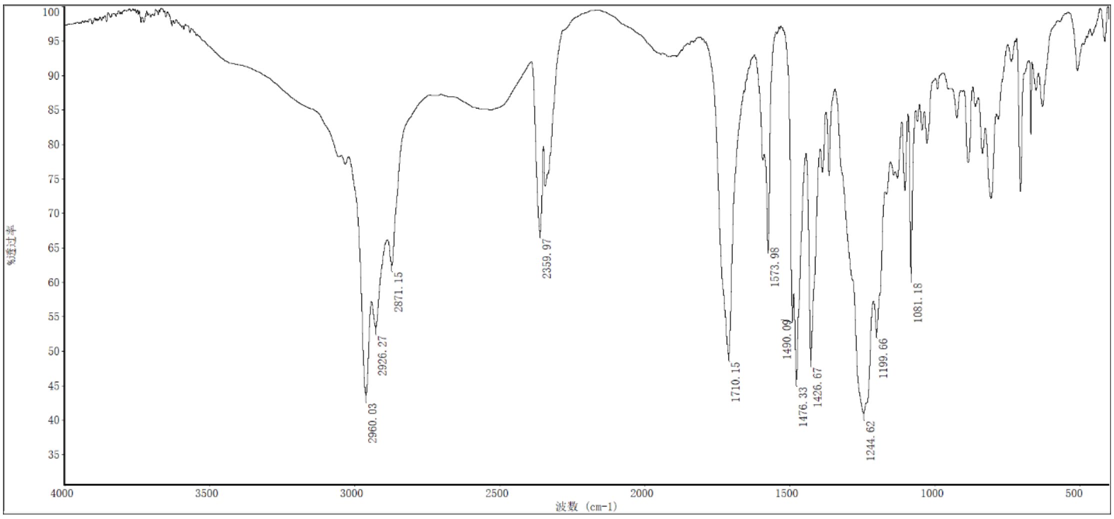

Figure S169. FT-IR spectrum of **7t**.

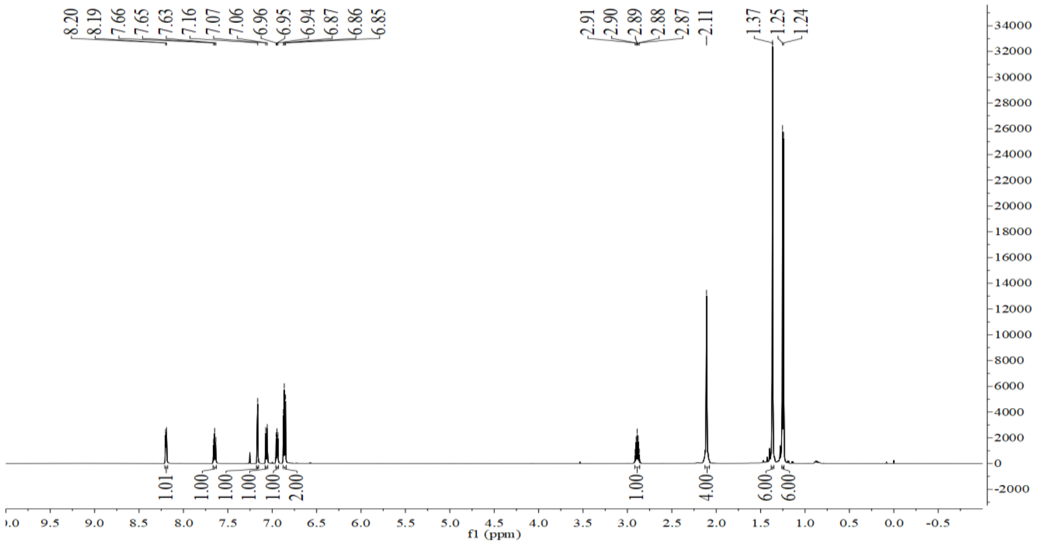

Figure S170. ^{1}H-NMR spectrum of **7t**.

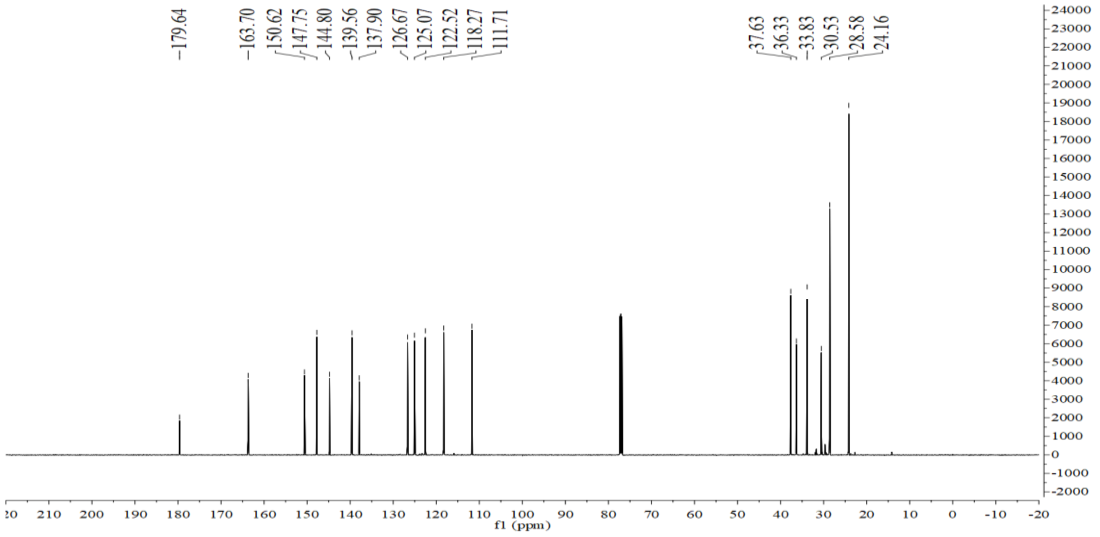

Figure S171. ^{13}C-NMR spectrum of **7t**.

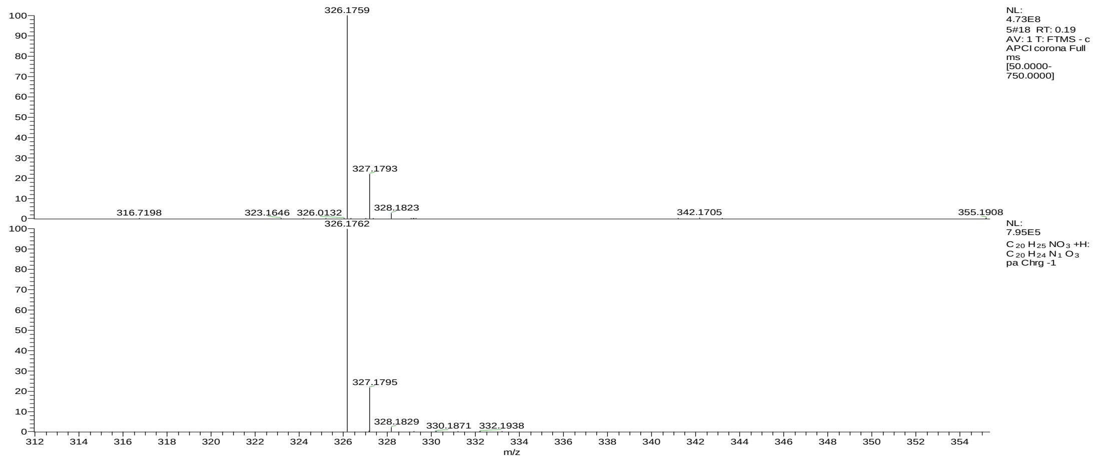

Figure S172. ESI-MS spectrum of **7t**.

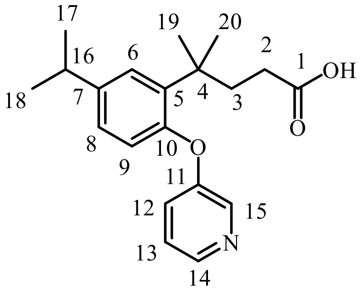

7u, white solid; yield 98.2%; m. p. 131.5– 132.6 °C; FT-IR (KBr, cm^{-1}): 2960, 2926, 2871 (C-H), 1710 (C=O), 1574, 1490, 1476, 1427, 1245, 1200, 1081 (Ar-C=C); ^{1}H NMR (600 MHz, $CDCl_3$) δ 8.31 (d, J = 23.2 Hz, 2H, C_{14}-H, C_{15}-H), 7.33 (s, 1H, C_{12}-H), 7.29 (s, 1H, C_6-H), 7.17 (s, 1H, C_{13}-H), 7.02 (d, J = 10.2 Hz, 1H, C_8-H), 6.71 (d, J = 8.3 Hz, 1H, C_9-H), 2.92 – 2.85 (m, 1H, C_{16}-H), 2.13 (dd, J = 26.7, 10.2 Hz, 4H, C_2-H, C_3-H), 1.40 (s, 6H, C_{19}-H, C_{20}-H), 1.24 (d, J = 6.9 Hz, 6H, C_{17}-H, C_{18}-H); ^{13}C NMR (151 MHz, $CDCl_3$) δ 178.01, 152.47, 144.67, 142.74, 139.96, 137.68, 127.03, 126.08, 125.27, 124.45, 119.79, 37.82, 36.32, 33.79, 30.74, 29.70, 28.44, 24.16; ESI-MS m/z: 326.1760 [M + H^+].

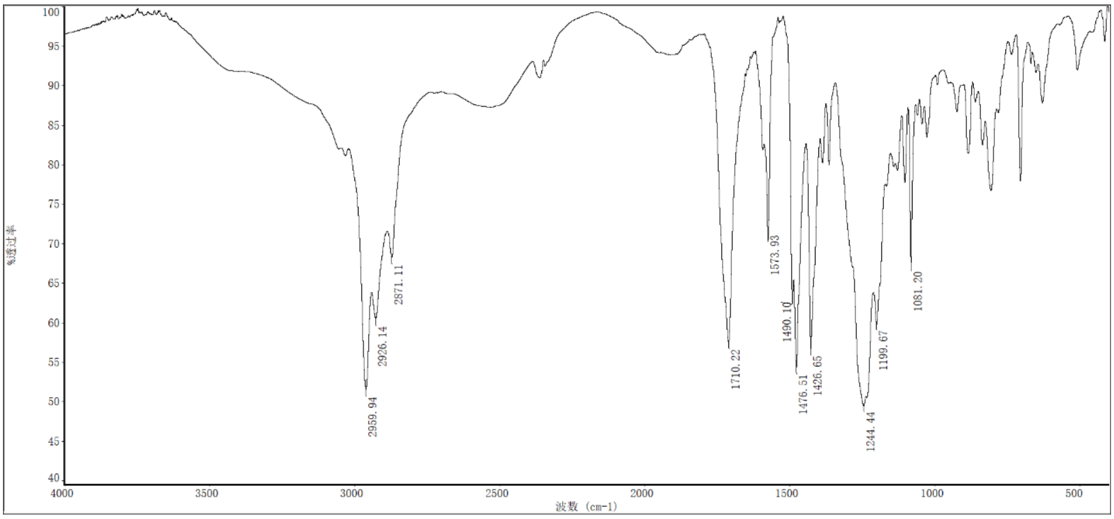

Figure S173. FT-IR spectrum of **7u**.

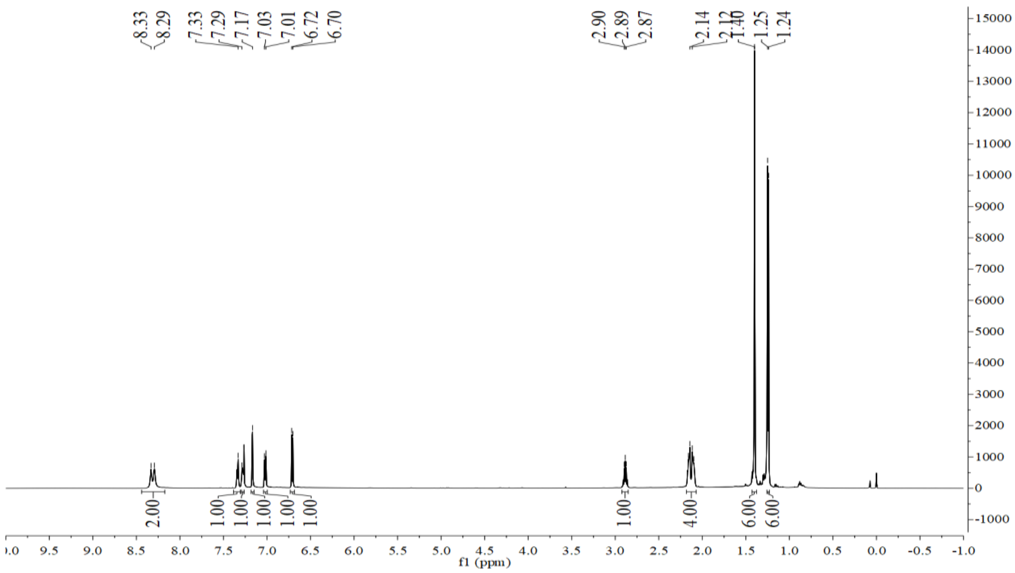

Figure S174. ^{1}H-NMR spectrum of **7u**.

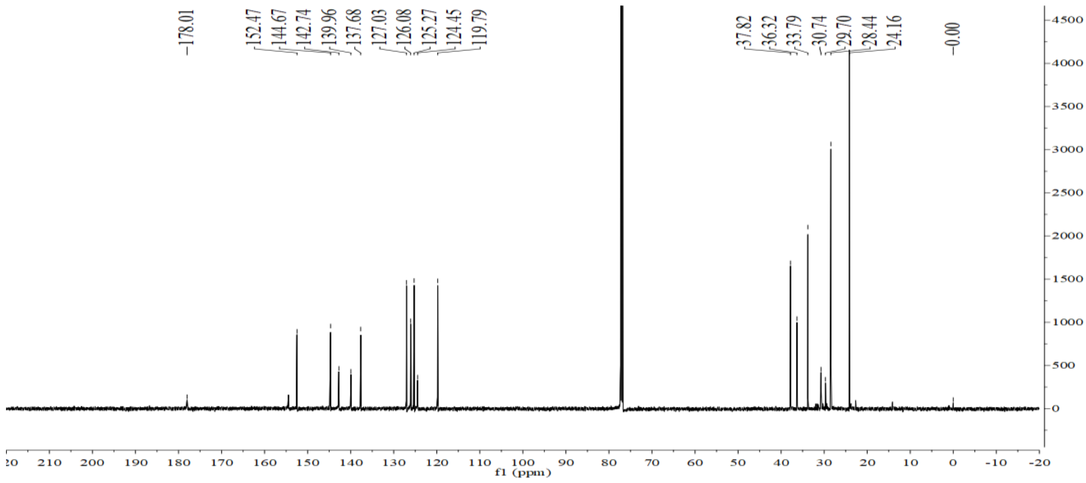

Figure S175. ^{13}C-NMR spectrum of **7u**.

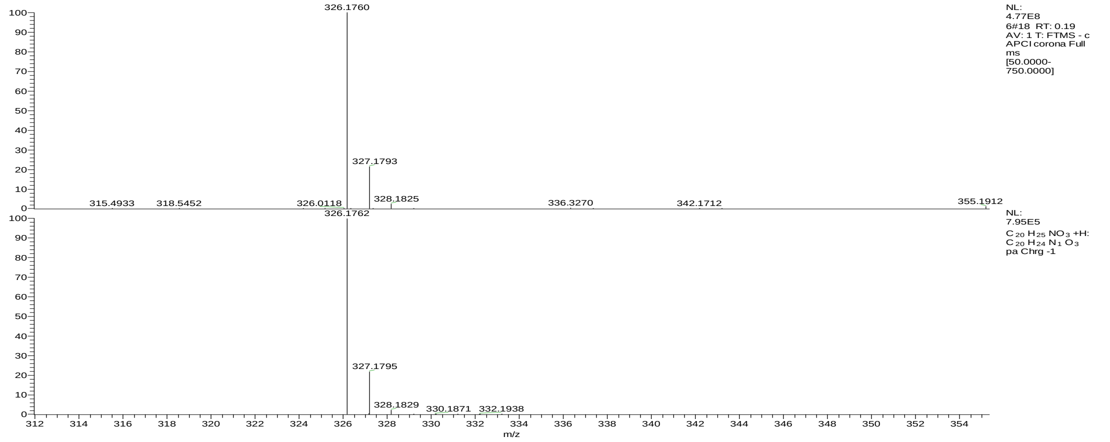

Figure S176. ESI-MS spectrum of **7u**.

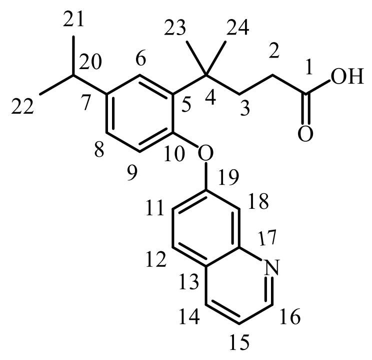

7v, white solid; yield 97.4%; m. p. 136.8– 137.2 °C; FT-IR (KBr, cm^{-1}): 2960, 2871 (C-H), 1710 (C=O), 1504, 1489, 1463, 1324, 1259, 1225, 1204, 1081 (Ar-C=C); ^{1}H NMR (600 MHz, $CDCl_3$) δ 8.62 (s, 1H, C_{16}-H), 8.01 (t, J = 7.9 Hz, 2H, C_{12}-H, C_{14}-H), 7.50 (dd, J = 9.2, 1.7 Hz, 1H, C_{18}-H), 7.28 – 7.26 (m, 1H, C_{15}-H), 7.21 (d, J = 12.4 Hz, 2H, C_6-H, C_{11}-H), 7.06 (d, J = 8.3 Hz, 1H, C_8-H), 6.84 (d, J = 8.1 Hz, 1H, C_9-H), 2.96 – 2.90 (m, 1H, C_{20}-H), 2.23 – 2.19 (m, 2H, C_2-H), 2.18 – 2.12 (m, 2H, C_3-H), 1.45 (s, 6H, C_{23}-H, C_{24}-H), 1.28 (d, J = 6.9 Hz, 6H, C_{21}-H, C_{22}-H); ^{13}C NMR (151 MHz, $CDCl_3$) δ 177.98, 156.11, 152.48, 147.66, 144.60, 143.60, 138.00, 136.01, 129.98, 129.31, 127.06, 125.1, 123.56, 121.34, 120.83, 112.02, 37.91, 36.35, 33.84, 30.94, 28.56, 24.21; ESI-MS m/z: 376.1916 [M + H^+].

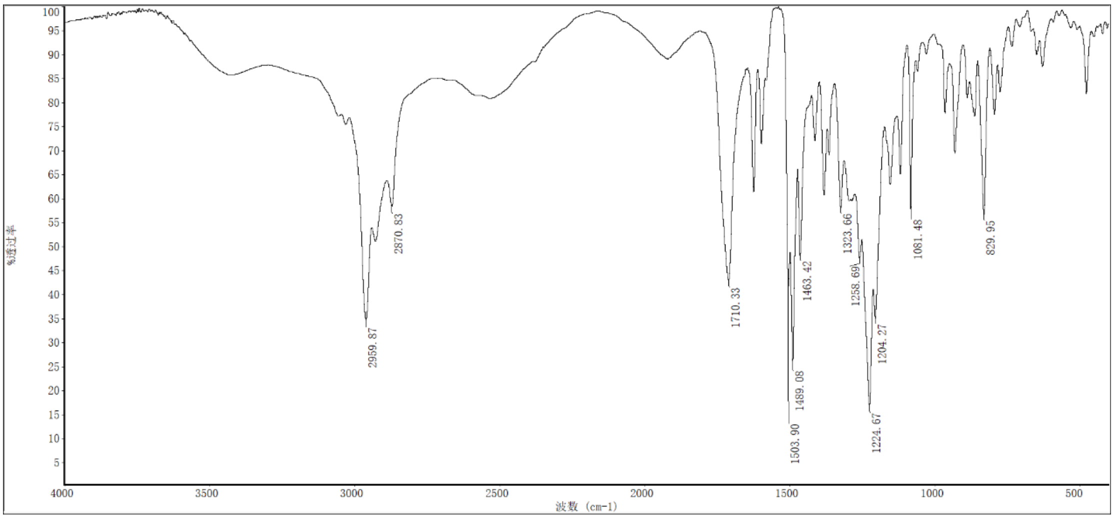

Figure S177. FT-IR spectrum of **7v**.

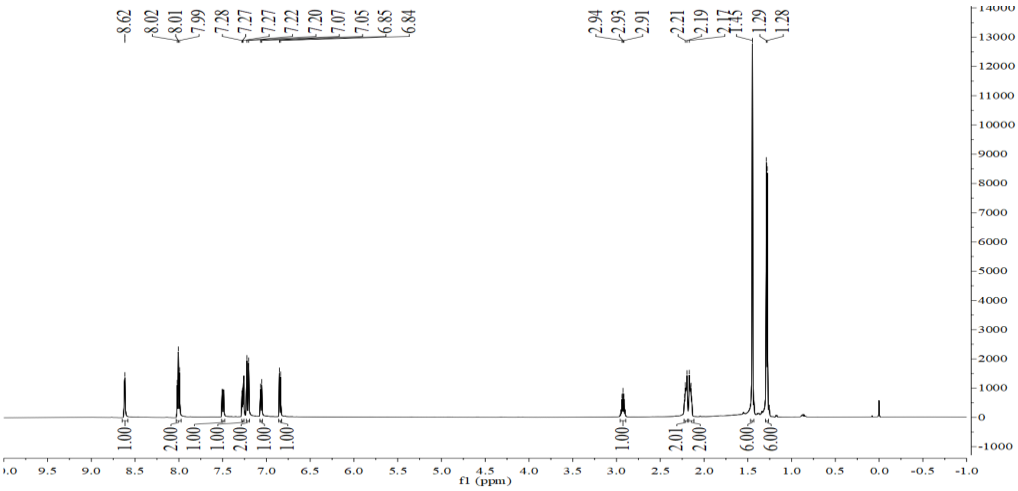

Figure S178. ^{1}H-NMR spectrum of **7v**.

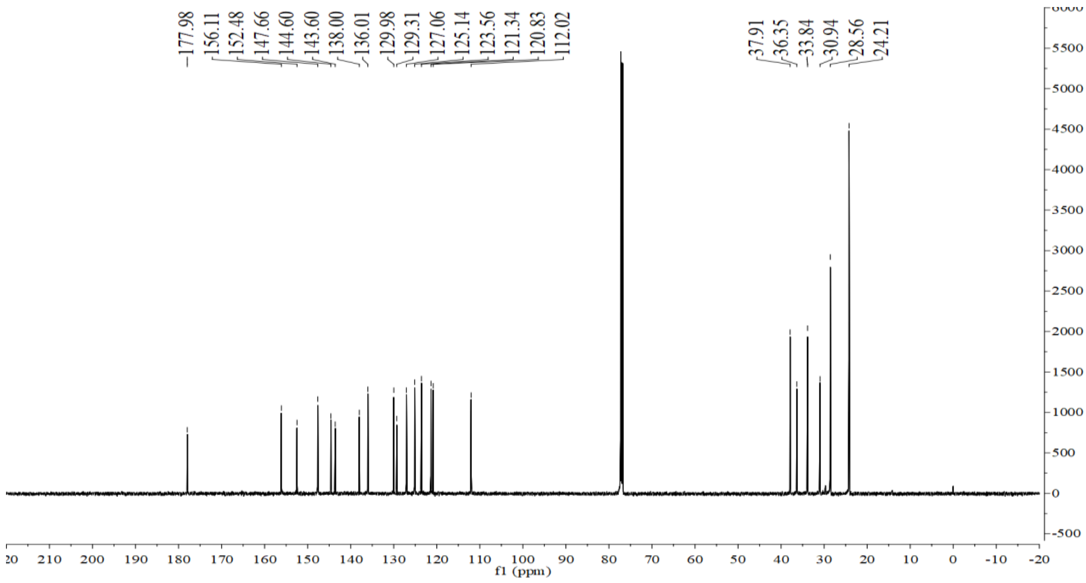

Figure S179. ^{13}C-NMR spectrum of **7v**.

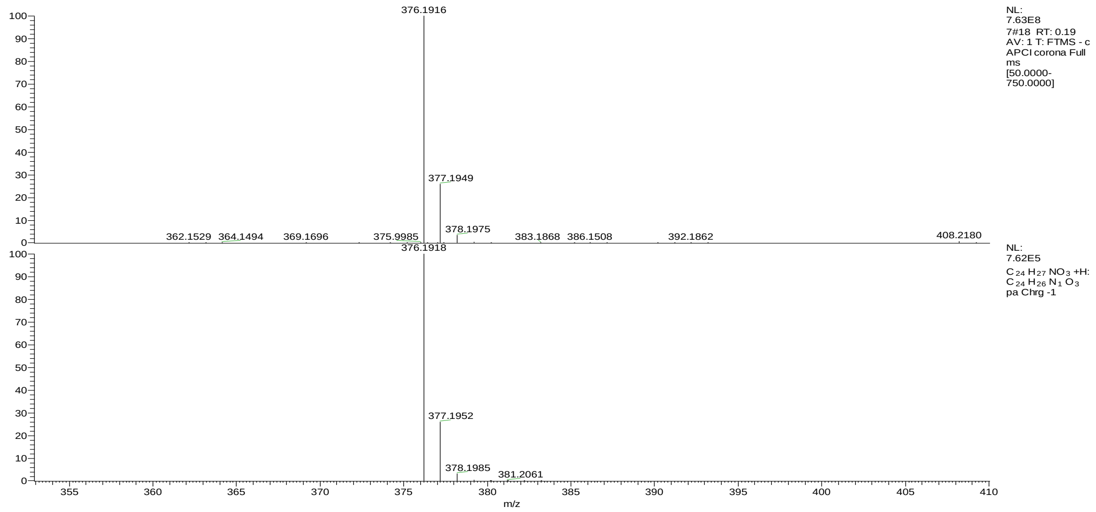

Figure S180. ESI-MS spectrum of **7v**.
